# Directed Molecular Stacking for Engineered Fluorescent Three‐Dimensional Reduced Graphene Oxide and Coronene Frameworks

**DOI:** 10.1002/open.201900310

**Published:** 2019-12-04

**Authors:** Boyang Mao, Fernando Cortezon‐Tamarit, Haobo Ge, Navaratnarajah Kuganathan, Vincenzo Mirabello, Francisco J. Palomares, Gabriele Kociok‐Köhn, Stanley W. Botchway, David G. Calatayud, Sofia I. Pascu

**Affiliations:** ^1^ Department of Chemistry University of Bath Claverton Down Bath BA2 7AY UK; ^2^ National Graphene Institute University of Manchester Booth Street East Manchester M13 9PL United Kingdom; ^3^ Faculty of Engineering, Environment and Computing Coventry University Priory Street Coventry CV1 5FB United Kingdom; ^4^ Department of Nanostructures and Surfaces Instituto de Ciencia de Materiales de Madrid – CSIC Sor Juana Inés de la Cruz 3, Campus de Cantoblanco Madrid 28049 Spain; ^5^ Department of Electroceramics Instituto de Cerámica y Vidrio – CSIC Kelsen 5, Campus de Cantoblanco Madrid 28049 Spain; ^6^ Central Laser Facility, Rutherford Appleton Laboratory, Research Complex at Harwell, STFC Didcot OX11 0QX United Kingdom; ^7^ Current address: Department of Engineering, Cambridge Graphen Centre University of Cambridge

**Keywords:** fluorescent graphene hybrids, fine control pore size, 3D hybrid materials frameworks, tuneable molecular self-assembly

## Abstract

Three‐dimensional fluorescent graphene frameworks with controlled porous morphologies are of significant importance for practical applications reliant on controlled structural and electronic properties, such as organic electronics and photochemistry. Here we report a synthetically accessible approach concerning directed aromatic stacking interactions to give rise to new fluorogenic 3D frameworks with tuneable porosities achieved through molecular variations. The binding interactions between the graphene‐like domains present in the *in situ‐*formed reduced graphene oxide (rGO) with functional porphyrin molecules lead to new hybrids *via* an unprecedented solvothermal reaction. Functional free‐base porphyrins featuring perfluorinated aryl groups or hexyl chains at their *meso*‐ and *β*‐positions were employed in turn to act as directing entities for the assembly of new graphene‐based and foam‐like frameworks and of their corresponding coronene‐based hybrids. Investigations in the dispersed phase and in thin‐film by XPS, SEM and FLIM shed light onto the nature of the aromatic stacking within functional rGO frameworks (denoted rGOFs) which was then modelled semi‐empirically and by DFT calculations. The pore sizes of the new emerging reduced graphene oxide hybrids are tuneable at the molecular level and mediated by the bonding forces with the functional porphyrins acting as the “molecular glue”. Single crystal X‐ray crystallography described the stacking of a perfluorinated porphyrin with coronene, which can be employed as a molecular model for understanding the local aromatic stacking order and charge transfer interactions within these rGOFs for the first time. This opens up a new route to controllable 3D framework morphologies and pore size from the Ångstrom to the micrometre scale. Theoretical modelling showed that the porosity of these materials is mainly due to the controlled inter‐planar distance between the rGO, coronene or graphene sheets. The host‐guest chemistry involves the porphyrins acting as guests held through π‐π stacking, as demonstrated by XPS. The objective of this study is also to shed light into the fundamental localised electronic and energy transfer properties in these new molecularly engineered porous and fluorogenic architectures, aiming in turn to understand how functional porphyrins may exert stacking control over the notoriously disordered local structure present in porous reduced graphene oxide fragments. By tuning the porosity and the distance between the graphene sheets using aromatic stacking with porphyrins, it is also possible to tune the electronic structure of the final nanohybrid material, as indicated by FLIM experiments on thin films. Such nanohybrids with highly controlled pores dimensions and morphologies open the way to new design and assembly of storage devices and applications incorporating π‐conjugated molecules and materials and their π‐stacks may be relevant towards selective separation membranes, water purification and biosensing applications.

## Graphical abstract

**By introducing a panel** of functional porphyrin molecules, new 3D hierarchical structures incorporating reduced graphene oxide can be achieved. The pore size of the framework can be finely tuned and reduced to a sub‐micron scale for the first time by careful selection of the porphyrin. Experimental and theoretical studies proposed that the pore size diminishing was due to strong local interactions between the designed porphyrin and rGO, mediated by donor‐acceptor interactions, which are maintained in the solution and in thin film.

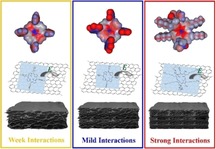

## 1 Introduction

Graphene foams with tailored functionalities are holy‐grail in the design and synthesis of new classes of organic materials due to their applications in energy storage, energy production and energy‐efficient storage and separations. The self‐ and directed‐assembly of graphene foams onto thin films are promising as they could enable a wide range of technological breakthroughs in areas such as the manufacturing of electronic semiconductor devices, optical materials, energy generation (e. g. thin film solar cells) and storage (thin‐film batteries). Such thin films also play an important role in the understanding, development and study of functional materials with new and unique properties. The limitations are due to the fact that to date wide access to such functional materials is limited by the currently available methods of production, which generally involve high temperature energy consuming processes, such as CVD or electrochemical depositions. Highly porous three dimensional (3D) frameworks stand out due to their wide applications in energy storage,^1^ flexible electronics,^2^ catalysis,^3^, ^4^, ^5^ biomedical, membrane technologies and environment‐related areas,^6^, ^7^ and there has been recent interest in the use of 3D graphene materials and water filtration /purification and biosensing applications. The properties of porous hierarchical structures not only depend on the nature of their basic building blocks, but also on the pore size and the specific surface area of the complex architecture, which determine the bulk physical‐chemical properties as well as their local electronic properties.^8^, ^9^, ^10^

Metal organic frameworks (MOFs), covalent organic frameworks (COFs) and zeolites have dominated the research field of self‐assembled nanomaterials due to the synthetic accessibility and the ability to control the pore size and their morphologies on practical laboratory as well as industrial scales. Recently, an alternative technique to generate functional porous structures was achieved through the use of self‐assembled two‐dimensional (2D) material into a 3D structure.^11^ Two dimensional materials endowed with unique ultrathin planar and well‐ordered architectures on an atomic level, in structures that show a broad range of attractive properties that dramatically different from their bulk phases. Graphene oxides (GO) and thermally reduced graphene oxides (rGO) have not yet been fully explored as supramolecular building blocks for 3D frameworks with controlled structural characteristics emerging from the organised assembly of 2D materials: this area of investigation constitutes a promising alternative to obtain porous 3D materials with well‐defined local properties.^12^, ^13^

Since the discovery of graphene by Geim and Novoselov^14^ this material has rapidly become the most intensively studied 2D structure due to its extraordinary mechanical, optical, thermal and electrical properties. Functional derivatives of graphene such as graphene oxide (GO) and reduced graphene oxide (rGO) have emerged as processable alternatives as they are synthetically accessible and manipulated using practical synthetic chemistry protocols on laboratory scale. The availability of functional rGO frameworks, which can combine micro‐, meso‐ and macropores on a scale ranging from Ångstrom to micrometres, remains rare.^9^ Access to such materials is, to date, rather scarce.^11^, ^15^ Their micro‐ and meso‐porosities endow high specific surface areas, while the macroporosity guarantees accessibility to this surface and renders their surface chemically reactive in a variety of applications. The elastic graphene matrix prevents the aggregation of active materials supported by 3D graphene and avoids the collapse, shrinkage, or decomposition of the backbone in the application process.

However, gaining pore size control in carbon‐based nanohybrids through a tight structural manipulation, and the factors influencing this self‐assembly, remain elusive. This limits further practical applications of functional graphene‐based materials. Current approaches to control the porosity of 3D graphene‐based framework rely mainly on the use of energy‐driven techniques such as high vacuum technologies.^16^, ^17^ Thus far, the porosity and the thickness of the stacked graphene layers have not yet been finely tuned and controlled on a molecular level by such techniques but the directed assembly of flat and aromatic molecules offers an alternative synthetic protocol.^18^ Chemical modifications of GO or rGO with organic molecules capable of ordered self‐assembly onto their surfaces allow for the spontaneous formation of new hybrid macrostructures and this directed assembly can influence their spectroscopic properties.

Current research by us, and others, aims to shed light on the fundamental chemical mechanisms that control the assembly of these hybrid structures and lead on the development of 3D hybrid (reduced) graphene oxides based frameworks, denoted functional rGOFs.^19^, ^20^, ^21^, ^22^ By accessing the advantages of non‐covalent interactions such as aromatic stacking, the top‐down exfoliation efficiency to fabricate graphene from graphite has been demonstrated.^23^, ^24^ To the best of our knowledge, there are only limited investigations into bottom‐up approaches to functional rGOF formations using the aromatic stacking of molecules and their rapid conversion to new 3D architectures with intrinsic porosity.

A deliberate choice of molecules capable to engage in non‐covalent interactions, for example porphyrins or phthalocyanines^25^ and perylene‐3,4,9,10‐tetracarboxylic dianhydride,^26^ allows the self‐assembling of such molecules onto 2D material surface, leading to a precise structural control, generating structural complexity.^27^, ^28^, ^29^, ^30^, ^31^, ^32^, ^33^ Tailored‐made functional porphyrins can direct the formation of complex structures when self‐assembled onto the graphene oxide surfaces and exhibit optoelectronic properties that retain the characteristic of the original porphyrin in terms of emission properties. These systems can also sense the local environment and can become a probe for local inhomogeneity in electronic properties, as evidenced by subtle changes in the fluorescence lifetime elucidated by imaging investigations in thin film.^34^ The directed‐ and self‐assembly mechanisms appeared to impart accurate thermodynamic control over the nature of supramolecular polymerisation of Zn(II)‐derivatised porphyrins onto graphene oxides or carbon nanotubes graphitic surfaces and the morphologies of the hybrids formed appear to be governed by a subtle balance between molecule‐graphene, molecule‐molecule, solvent‐graphene and molecule‐solvent interactions.^31^

**Figure 1:**
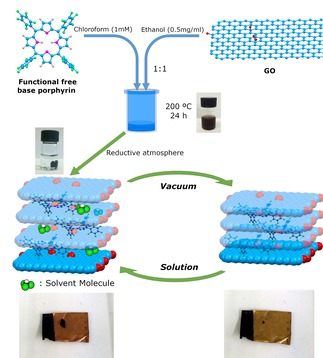
Schematic representation of the formation and foam behaviour of the free base porphyrin@rGO frameworks for porphyrins **1**, **2** and **3**. The represented porphyrin structure corresponds to the perfluorinated porphyrin **2**, as an example. Control experiments involving porphyrins alone did not indicate significant alterations in the nature of the porphyrin cores. The digital photographs show the formation of a 3D ordered framework after the solvothermal reaction and the foam behaviour in presence of solvent of porphyrin **2**@rGO material (CHCl_3_). The consistency of the reversibility of the process was tested by specific surface area determination by BET and repeated 5 times.

By using synthetic scaffolds such as GO, rGO or coronene (as a molecular building block modelling the local structure of a graphene fragment) new 3D porous structures were generated *via* a solvothermal conversion in the presence of porphyrins and the products were structurally investigated (*vide infra*). The main objective of the study is a facile one‐step method to generate new 3D rGO hybrid frameworks from functional free base porphyrins and corresponding graphene oxide precursors *via* a facile *in situ* solvothermal reaction (Figure 1).

We investigated hereby a facile route to control the porosity of new 3D structures by molecular level stacking interactions involving aromatic interactions as well as likely H‐bonds between free base porphyrins and the graphitic sheets. The synthesis involved a supramolecular polymerisation process first, followed by hydrothermal annealing of *in‐situ* pre‐formed 2D porphyrin‐graphene oxide nanohybrids to give the new 3D hybrids. This approach can provide a scalable synthetic scaffold to obtain mixed organic/inorganic nanohybrids with similar functions to MOFs, COFs or zeolites.

## 2 Results and Discussion

This synthetic approach relies on a supramolecular polymerisation route followed by hydrothermal annealing of *in situ* pre‐formed porphyrin‐graphene oxide hybrids. We report hereby a facile, all‐in‐one, single‐step solvothermal protocol to obtain a new family of three‐dimensional fluorescent and porous porphyrin@rGO frameworks. New and synthetically‐ accessible foam‐like three dimensional (3D) fluorogenic frameworks, incorporating reduced graphene oxide (rGO, formed *in situ* from its GO precursor) or coronene (as a molecular model for a graphene fragment) were synthesised. A new *in situ* solvothermal protocol reliant on molecular self‐assembly was devised to give rise to new 3D nanohybrids. The directed self‐assembly of functional porphyrins with reduced graphene oxide layers, as well as, coronene for the formation of new nanohybrids was investigated in solution and in thin film.

**Figure 2:**
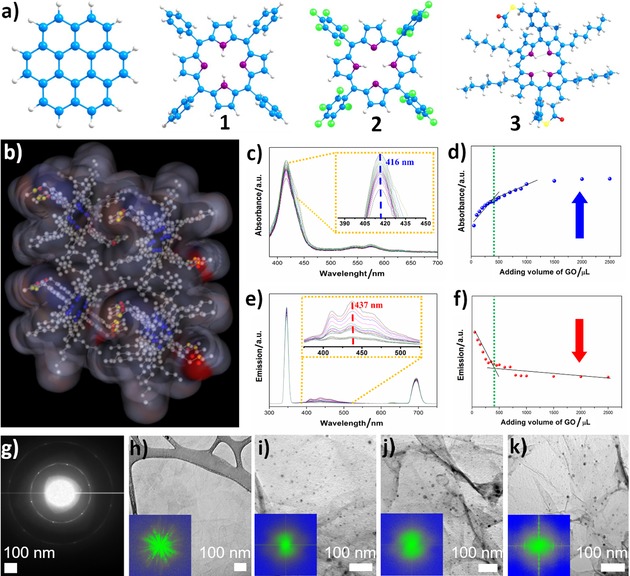
(a) DFT simulated relaxed molecular structure of: coronene, *meso*‐tetraphenylporphyrin, **1**; 5,10,15,20‐tetrakis(pentafluorophenyl)porphyrin, **2**; 5,15‐di(3‐acetylthiomethylphenyl)‐2,8,12,18‐tetra‐n‐hexyl‐3,7,13,17‐tetramethyl‐porphyrin, **3**. (b) Solvent accessible surface electrostatics potential of a fragment of a unit cell of **3**; (c) UV‐visible absorption enhancement behaviour in the spectroscopy of **3**@GO; (d) UV‐visible absorption normalised intensity with respect to added volume of the graphene oxide; (e) Fluorescence emission quenching behaviour in **3**@GO; (f) Fluorescence emission normalised intensity with respect to added volume of the graphene oxide; Representative images showing: (g) SAED of graphene oxide starting materials; Representative images showing TEM micrographs and insert FFT patterns of (h) graphene oxide; (i) **1**@rGO complex framework; (j) **2**@rGO complex framework; (k) compound **3**@rGO complex framework.

A panel of porphyrins with well‐understood structural characteristics (Figure 2a), ranging from simple to more complex (**1**–**3**), were employed in the synthesis of nanohybrids to act as the ‘molecular glue’ capable to mediate the bonding force between rGO sheets. The porphyrin systems of choice were: meso‐tetraphenylporphyrin, **1**, 5,10,15,20‐tetrakis(pentafluorophenyl)porphyrin, **2**, a perfluorinated derivative of **1**, as well as 5,15‐di(3‐acetylthiomethylphenyl)‐2,8,12,18‐tetra‐n‐hexyl‐3,7,13,17‐tetramethylporphyrin, a bulky derivative denoted **3**. The bulky porphyrin **3** has been previously synthesised on a laboratory scale^34^ and employed hereby for the *in situ* transformation because, unlike porphyrins **1** and **2**, this compound exhibits two aryl thioacetate‐functionalised side groups at the *meso*‐positions and four hexyl chains at the *β*‐position which increase the overall hydrophobicity and solubility in most common organic solvents.^27^, ^34^ For porphyrin **3**, the single crystal X‐ray diffraction studies showed that molecules are oriented face‐on and show two hexyl chain groups placed anti with respect to the aromatic core.^27^, ^34^ This free base porphyrin incorporating functionalised hexyl chains was selected as these aliphatic groups were deemed able to separate individual porphyrins from other molecular species. This molecular design is advantageous here as minimises the porphyrin‐porphyrin core self‐stacking, thus facilitating the interactions between porphyrins and electron acceptors. Furthermore, the inclusion of organic linkers at the *meso*‐ or *β*‐positions was highlighted to dramatically change/enhance the electron movements or injection ability from **3** to the electron acceptor, and such phenomena are observable in thin film measurements by fluorescence lifetime imaging microscopy (*vide infra*).

Titrations of coronene with porphyrins **1**–**3** (Figures S2–4) as well as the analogous titrations of porphyrins **1**–**3** with GO dispersions were carried out first, using optical absorption and fluorescence emission spectroscopies. In all cases, binding isotherms were fitted for the systems described here using an analogous approach to that described for the binding between **3** and coronene or graphene fragments. Figure 2c shows the optical absorption spectra of compound **3** dispersed in ethanol (1×10^−6^ M) and then titrated with a GO solution in ethanol (0.05 mg mL^−1^). The main absorption peak of compound **3** at 416 nm was enhanced when the GO solution was added. Figure 2d shows the absorption spectroscopy of compound **3** upon addition of GO at λ=416 nm. The same experimental conditions were applied to compound **3** and GO in the fluorescence emission spectroscopy measurements. By exciting at 346 nm, the emission peak observed at 437 nm was found to decrease in intensity after aliquots of GO dispersion were added (Figure 2e). Figure 2f shows the normalised emission intensity with respect to the added volume of the rGO dispersion and relevant quenching phenomena observed. Both UV‐vis and fluorescence spectroscopy exhibit a critical point of about 398 μL (0.195 mg of rGO were added), which may be due to a cooperative supramolecular aggregation model.^35^ The enhanced UV‐vis absorption together with the general fluorescence emission suggest the formation of electronic interactions between rGO and compound **3** in its ground and photoexcited state, indicating the formation of the **3**@rGO complex and the strong bonding behaviour.

Upon mixing of the building blocks (functional porphyrins **1**–**3** with the carbon supports, either graphene oxide or coronene), the dispersion became an intense purple colour, and these dispersions were characterised spectroscopically (ESI) and the binding interactions evaluated by fluorescence titrations and corresponding binding isotherms (ESI). Solvothermal synthesis for the *in situ* formation of porphyrins hybrids directly from the GO complexing denoted **1**–**3**@GO was performed on a laboratory scale. This provided an efficient and low temperature route to reduce GO *in situ* to give the corresponding **1**–**3**@rGO hybrids. This method can promote graphitisation of amorphous carbon, initiate dihydroxylation on the edge or basal planes of GO and induce π‐conjugation recovery.^36^, ^37^, ^38^ To minimise the possible variations originating from the experimental parameters, all 3D hybrids were synthesised under the same experimental conditions, whilst varying only the nature of the initial functional porphyrin applied. After the solvothermal annealing reaction of hybrid dispersions of **1**–**3**@GO, whereby the GO simultaneously was reduced to rGO *in situ* to give rise to the corresponding **1**–**3**@rGO hybrids, the formation of black solids was observed in each case. These were collected by mechanical separation and fully characterised spectroscopically and by a range of microscopy techniques (ESI). The nature of the interactions was studied by X‐ray Photoelectron (XPS) and Raman Spectroscopies (ESI).

Theoretical investigations by semiempirical and Density Functional Theory (DFT) level calculations (ESI) probed the hypothesis that subtle changes in the peripheral structure of the porphyrin guests and corresponding electron density, as modulated by peripheral substituents, can affect the aromatic interaction with rGO and coronene (a molecular‐level model for the rGO), thus affecting the nature and the fluorescence properties of 3D nanohybrids. The solvent accessible surface electrostatics potential of packed cell structure of these molecules was estimated by semi‐empirical calculations. The calculated partial charge levels are represented in Figure 2b as spheres, indicating that compound **3** is capable of interacting with approaching protons or positively charged species. The red colour in Figure 2b is used to represent the negative electrostatic potential, which corresponds to the attraction of the proton by the concentrated electron density. The blue colour was used to exhibit the positive electrostatic potential agreeing with the repulsion of the proton by the atomic nuclei in the regions where lower electron density exists and the nuclear charge is incompletely shielded. The calculated electrostatic potentials suggested that compound **3** allows more non‐polar solvent to gain access to its surface. The nature of the host‐guest interactions was explored by DFT (See ESI).

Transmission Electron Microscopy (TEM) and High‐Resolution Transmission Electron Microscopy (HR‐TEM) were used to obtain direct morphological information of the GO and the corresponding functionalised rGO structures. (Figures 2h–k). Three different hybrid structures present similar morphology on the nanometre scale and it is suggested that in each case the porphyrin molecules are incorporated onto the rGO surface. The insert FFT patterns in Figures 2h–k indicate that all three composites have an amorphous phase that utterly differs from pristine GO due to the porphyrin decoration. The HR‐TEM (Figure S60–1) and TEM micrographs (Figure 2i) of GO show that the carbonaceous sheets were rotated‐oriented with respect to other flakes and the insert Fast Fourier Transform (FFT) shows that GO was partly crystalline. Selected Area Electron Diffraction (SAED) analysis of the resulting rGO hybrids shows a diffraction pattern with a six‐fold rotational symmetry, which is expected for a diffraction with the beam impacting along the [001] direction (Figure 2h). The innermost diffraction spots are from (100) planes (d‐spacing 0.21 nm) while the outer is from (110) planes (d‐spacing 0.12 nm).

**Figure 3:**
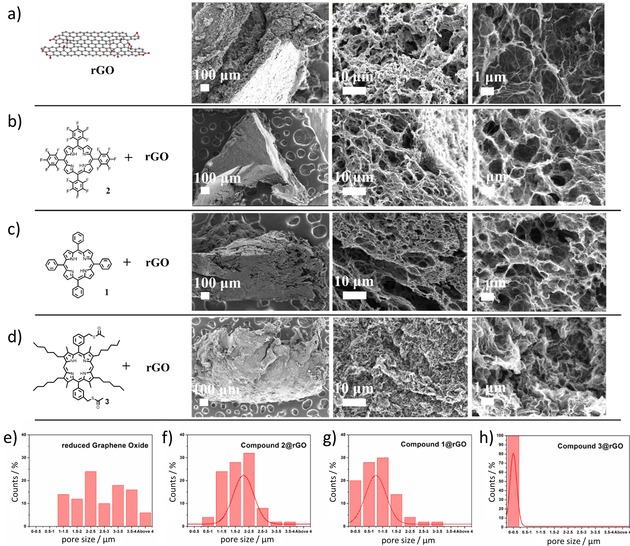
SEM images. Scale bar is 100 μm, 10 μm and 1 μm respectively (a) rGO; (b) **2**@rGO composite; (c) **1**@rGO composite; (d) **3**@rGO composite. Histogram of pore sizes (N=100) as measured on SEM (e) rGO; (f) **2**@rGO composite; (g) **1**@rGO composite; (h) **3**@rGO composite.

Detailed porosity and morphology investigations of porphyrin@rGO frameworks were carried out to predict structure‐function correlations by Scanning Electron Microscopy (SEM) and BET investigations. SEM measurements were carried out aiming to characterise the porphyrin@rGO hybrids morphology. Highly oriented pyrolytic graphite (HOPG) was applied as the supporting substrate to reveal important feature in terms of the pore size. From the micrographs (Figure 3a–d), the pore size of each 3D framework was then measured directly from SEM images using ImageJ 1.52.^39^ The measuring points were randomly chosen and the histograms of the measured results (number of measurements, N=100) were presented in Figures 3e–h. Figure 3e suggests that the pore size of pristine rGO framework varies in the 1–4 μm range with a non‐normal distribution (i. e. which did not fit to a normal distribution, e. g. Gaussian, Gauss or Laplace‐Gauss). Although **2**@rGO and **1**@rGO present a wide distribution of pore size, most of these (over 90 %) of **2**@rGO are in the 1–3 μm range; and it is smaller for **1**@rGO (below 2 μm). For **3**@rGO, the further trend towards a diminishing pore size became more intense. This suggests that the presence of porphyrins in the initial reaction mixture represents the major process triggering the formation of a smooth surface and pores with a specific size. We propose here that the inter‐particle forces involved at the contact points of each 3D framework vary due to the interactions between rGO and porphyrin molecules, thus the local structure may well be altered accordingly on the nanoscale. Previous studies reporting solvothermal/hydrothermal fabricated graphene 3D structures generally led to a relatively large pore sizes of around 5 μm or above.^40^, ^41^, ^42^, ^43^, ^44^, ^45^ There are only very few studies reporting the reduction of the pore size by a pristine supramolecular self‐assembly with small molecules.

The porosities of the nanostructured hybrids were probed by specific surface area measurements (ESI, Table S2). BET measurements revealed that **1**@rGO presents the highest specific surface area (211 m^2^/g); this is probably due to a smaller size of the 3D frameworks than **2**@rGO (46 m^2^/g) and **3**@rGO (58 m^2^/g). The pore structure of the samples was analysed by N_2_ adsorption/desorption measurements, the pore size distribution being calculated by the density functional theory method: **1**@rGO, 33.1 Å; **2**@rGO, 45.7 Å and **3**@rGO, 31.7 Å.

The obtained pore sizes agree with the SEM results. In addition, the volume of the porphyrin functionalised samples dried under vacuum decreased their volume almost 10 fold. When the samples were dispersed again in the same solvent (CHCl_3_, 1 mg/mL) they recovered the original volume maintaining the structure. The volume of the sample also changed when the materials were dispersed in different organic solvents and these expansion/contraction of volumes were measured by BET(ESI). This indicates the flexibility of these 3D structures to expand and contract their volume to accommodate solvent molecules between the rGO planes without losing their local structural integrity and is in agreement with the initial semiempirical predictions (Figure 2).

The appreciation of the nature of the bonding interactions and of the substantial energy transfer between porphyrins and coronene building blocks is paramount to gain an insight into the likely thermodynamically stability of porphyrins‐rGO hybrids. As such, coronene was employed as a molecular model for a small fragment of graphene layer, allowing our insight into the strength of the porphyrin‐coronene interactions and an evaluation of local electronic structures and charge transfer interactions.

**Figure 4:**
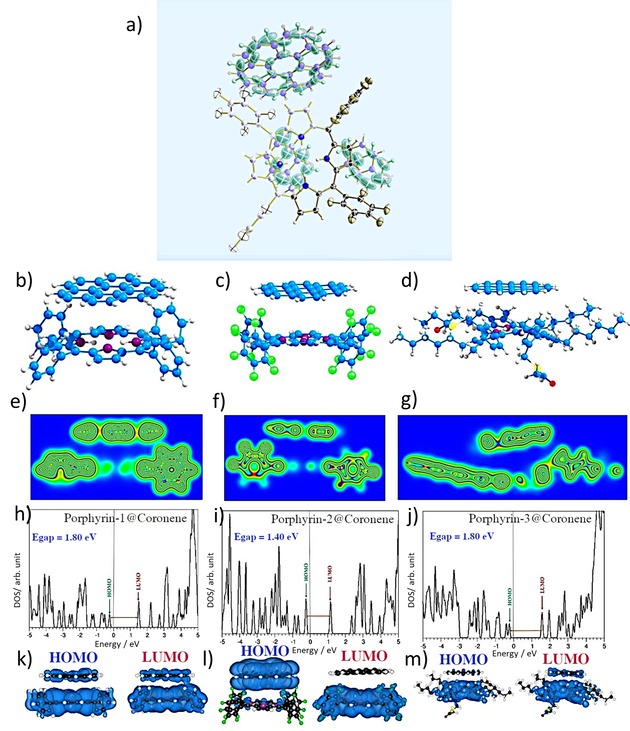
(a) Single crystal X‐ray structure for **2**@Coronene (asymmetric unit, thermal ellipsoids at 50 % probability); (b), (c) and (d) DFT relaxed structures of **1**@coronene, **2**@coronene and **3**@coronene respectively; (e–g) corresponding charge density plots; (h–j) densities of states; (k–m) the corresponding HOMO‐LUMO plots for the DOS.

DFT‐level theoretical modelling was performed on three starting porphyrin structures (**1**–**3**) using the structural information available from single crystal X‐ray diffraction data identical to the used in the solvation calculations, and these were then considered to model the three porphyrins@coronene structures at ab‐initio levels. The relaxed porphyrin@coronene model structures are shown in ESI and Figure 4b–d. The calculated distances at the binding of porphyrins to coronene are given in Table S8. The calculated C−C, C−N and F−H distances are longer than 3.80 Å indicating a non‐covalent interaction. Calculations were performed hereby using dispersion and non‐dispersion considerations to shed light on how van der Waals interactions control the nature of these hybrid materials. Relaxed configurations reveal that aromatic rings and long chain alkyl groups attached to the porphyrins prevent complete binding between the porphyrin and coronene.

Explorations into the electronic properties for the composites were also performed, using dispersion calculations. It was shown that there is a binding interaction in gas phase between porphyrins and coronene in each case. However, the calculated binding interactions are difficult to justify on basis of the structural differences since this may be configuration‐dependent. Interestingly, the charge transfer evaluated by DFT was also very small with respect to the normal values expected/reported to two C−C π systems interacting through aromatic interactions and whilst the geometries available may suggest that the phenyl rings placed almost perpendicular to the porphyrin ring hinder the interactions between the porphyrin plane and the coronene. DFT relaxed structures of **1**@coronene, **2**@coronene and **3**@coronene respectively are all in agreement with the molecular parameters determined. However, the orientation of coronene vs toluene differs greatly in the crystal structure, showing a preference for toluene molecules to stack with porphyrin planes, and of coronene to bind to the electron‐depleted perfluorinated rings in **2**@coronene.

We compared the theoretical structure of **2**@coronene and the molecular structure obtained from single crystal X‐ray diffraction (Figure 4a, Tables S3–5 and Figure S12). In the obtained experimental data, the π‐π interaction is established between the porphyrin core and the toluene molecules forming porphyrin‐toluene stacking columns. Corresponding charge density plots (e–g), densities of states (h–j) and HOMO‐LUMO plots (k–m) are reported hereby. Interestingly, the single crystal X‐ray diffraction data determined for **2**@coronene in single crystals grown from toluene further highlighted an interesting mismatch in the configuration between these planes in the presence of toluene, with respect to the gas‐phase case. This might well explain the rather small charge density exchange and the difficulty in fitting a binding isotherm in the case of **2**@coronene formation in solution monitored by fluorescence titrations (ESI). It also points out to a likely reason why the presence and nature of solvent molecules might well influence the reversibility of porosity of the rGO composites, as consistently indicated by BET measurements.

To elucidate the local electronic properties of the monohybrids **1**@coronene, **2**@coronene and **3**@coronene, the calculated binding energies and the charge transfer that occurs from coronene to porphyrin in corresponding hybrids were evaluated. Data in Table 1 and Table S9 summarise these binding energies. Interestingly, when these were calculated without dispersion forces considerations the values found are almost zero and the differences in the binding energies show the necessity of including dispersion in the models. The negative binding energy values calculated using dispersion indicates that the complexes formed between porphyrins and coronene are thermodynamically stable and exhibit non‐covalent nature. The magnitude of the binding interaction depends on the different structure of the porphyrin. Porphyrin **1** and **3** present stronger bonding force to coronene compare with porphyrin **2**. For the case of **3**@rGO complex, it is likely that the four long hexyl chains present may well have an ability to further interact with the extended hydrophobic rGO surfaces. Thus, the interaction between **3** and rGO 2D sheets is likely to be the most effective, while such bonding between **2** and rGO is the weakest.

**Table 1:** Calculated binding energies of porphyrins interacting coronene using dispersion (DFT+D) and without dispersion (DFT) and the estimated charge transfer using Bader approximation^46^ and the charge transfer occurred from coronene to porphyrin.

Composite	Binding Energy (eV)		Charge transfer	
	DFT	DFT+D	Estimated in this simple model (1 : 1)	Possible model with maximum coverage (4 : 1)
**1**@coronene	−0.02	−0.86	0.0128	0.0512
**2**@coronene	+0.06	−0.52	0.0391	0.1564
**3**@coronene	+0.10	−0.86	0.0105	0.0420

To estimate the charge transfer between porphyrin and coronene, the Bader charge analysis was carried out for the 1 : 1 complexes. Based on the charge transferred between a single porphyrin and coronene, we estimated the charge transfer is indicative of a possible 4 : 1 complex in each case. This explains the complexity in data fitting for the solution formation of porphyrin coronene and the nature of the host‐guest chemistry involved had to be elucidated using the information from single crystals X‐ray diffraction. In all cases, a small amount of charge transfer was also observed to occur from coronene to porphyrin confirming the non‐covalent binding nature.

To investigate the effect of non‐covalent interactions and their cooperativity on the hybrid formation and elucidate how the different π‐π stacking forces may affect the interaction between porphyrins and rGO (which in turn affects the composite pore size) we adopted the simple Hunter and Sanders^47^, ^48^ concept, which has been applied widely upon describing aromatic stacking model in fundamental supramolecular chemistry.

**Figure 5:**
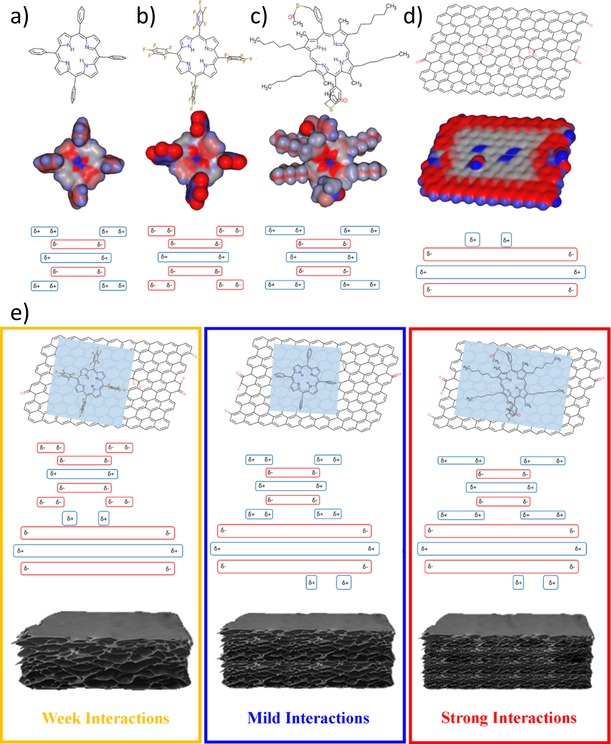
Semi‐empirical level optimised geometries of: (a) compound **1**; (b) compound **2**; (c) compound **3** and (d) GO (red denotes areas of relatively high electron density, blue denotes electron deficient areas); (e) proposed stacking arrangements between compounds and GO and schemes show the formed 3D structures.

These explorations were expanded upon by theoretical calculations of electrostatic surface potentials using the optimised structure of compounds **1**–**3** and a fragment of rGO as a model (Figure 2b and Figures 5a–d). The structure optimisation was carried out using an MMFF94 Force Field in Avogadro software. The aromatic (π‐π) interactions were modelled here using a simplified face‐centred π‐stacking approach between an electron‐rich aromatic molecule, which acts as a ‘donor’ and an electron deficient aromatic compound that acts as an ‘acceptor’. These are a subset of aromatic interactions, which differ from electron neutral or rich aromatic stacking which is usually edged to face or in an offset conformation.

Observations from spectroscopic measurements and computational simulations are in line with earlier reports^32^ and indicate that the combination of porphyrin and graphitic layers occurs via a face‐face orientation and the two systems in the donor‐acceptor nanocomplex are very closely held together in these systems. AFM investigations are in agreement with this observation and and this is particularly evident in the HOPG substrates. This is in line with observations reported by us, and others, at the self‐assembly of porphyrins onto the surface of other extended aromatic nanomaterials such as carbon nanotubes.^26^, ^32^

The theoretical investigations suggest that compound **3** interacts with GO more favourably than compound **1** and **2** because of the π‐π interactions and geometry of the porphyrins and GO as shown in Figure 5e. Due to the π‐π stacking, the porphyrin molecules can be considered to act hereby as the “molecular glue” capable of linking together graphene sheets. Compound **3** provides the strongest interactions, reducing most effectively the 3D framework pore size with respect to **1** and **2** respectively. This agrees with the experimental results obtained from SEM images and BET and thus supporting that the bonding strength of this non‐covalent linkage and can be used to finely tune the final structure of the resulting 3D frameworks. Unlike the pure rGO frameworks, in the presence of organic solvents these new functionalised 3D‐frameworks incorporating porphyrin hybrids can expand and subsequently reverse their pores volume depending on their environment, as evidenced by BET measurements. Under reduced pressure, the synthesised frameworks compact their layers showing the lower porosity, but after re‐suspension in common organic solvents the composites structure expand and accommodate the solvent molecules.

**Figure 6:**
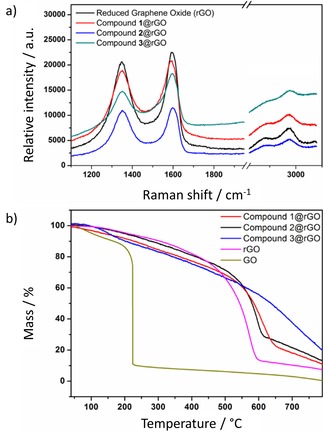
(a) Raman spectroscopy of solvothermally reduced rGO, **1**@rGO, **2**@rGO and **3**@rGO at selected ranges of the spectra; (b) TGA‐curves of GO, rGO and graphene@porphyrin composites.

An insight into the functional and fluorescence properties of porphyrin@GO framework was gained from spectroscopic investigations in the dispersed phase and in bulk as well as in thin film. The Raman spectra of hybrids **1**–**3**@rGO were recorded using a laser wavelength excitation of 514 nm, a region in which there was little interferences from porphyrin fluorescence emission (Figure 6a and Table S6). All G bands of porphyrin@rGO composites were found to be red‐shifted, suggesting an electronic coupling between rGO and porphyrin molecules, this is in line with previous investigations.^49^ The I_D_/I_G_ ratios (estimated in the Raman spectra of rGO, **1**@rGO and **2**@rGO) were all of similar magnitudes, while **3**@rGO composite presented a relatively lower I_D_/I_G_ ratio. All rGO frameworks exhibit emerging 2D and D+G peaks at ca. 2800 cm^−1^ indicating the defect minimisation with the aromatic network upon functionalisation.^50^ To study the physical and chemical properties of porphyrin@rGO porous structures obtained, thermogravimetric analysis (TGA) investigations were carried out in air, from 0 °C to 900 °C (Figure 6b and ESI, Table S6). TGA measurements indicate that the decomposition temperatures of functionalised rGO framework increased in the trend of increasing bonding strength which indicates a more stable structure.^18^

**Figure 7:**
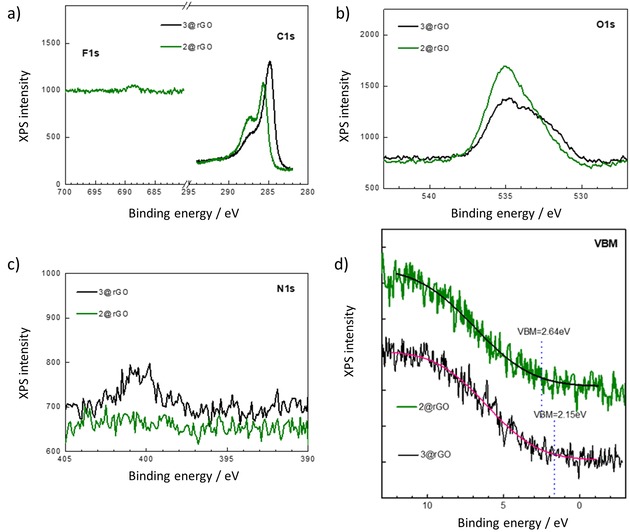
High‐resolution XPS spectra corresponding to the VBM region for **2**@rGO and **3**@rGO. (a) C1s and F1s regions for **2**@rGO and **3**@rGO, (b) O1s region for **2**@rGO and **3**@rGO, (c) N1s region for **2**@rGO and **3**@rGO, and (d) VBM region for **2**@rGO and **3**@rGO samples.

Figure 7 shows the XPS data recorded for **2**@rGO with a focus on the O, F, N and C‐characteristic regions. Sharp peaks located at around 284.9 eV and 285.7 eV correspond to C=C/C−C in aromatic rings and C−O in hydroxyl and epoxy groups, respectively.^27^ The peak corresponding to a binding energy of 287.4 eV corresponds to the carbonyl functional groups. In the same figure, it can be observed the F1s region in which a peak around 688.7 eV is observed for the **2**@rGO sample confirming the presence of the C−F groups from the porphyrin.^51^ Two oxygen signals situated at 535 eV and 532 eV were detected, which can be assigned to C−O−H and C−O respectively.^27^ Finally, the presence of a signal around 400.5 eV in the N1s region confirms the presence of N in the samples and therefore the formation of porphyrin@rGO materials The valence band maximum (VBM) of **2**@rGO and **3**@rGO (Figure 7d) is 2.64 eV vs vacuum level for **2**@rGO and lower, ca. 2.15 eV, for **3**@rGO sample. This tendency agrees with the values observed in the DOS calculations by DFT using coronene as a model system.

As seen in the titration experiments there is a strong quenching effect due to a close level interaction between the rGO and each of the porphyrin used, however the nature of the substituent heavily influences the excited state characteristics. The local interactions between porphyrins and rGO and consequent donor‐acceptor energy transfers were probed *via* single‐ and multi‐photon fluorescence lifetime imaging microscopy (FLIM) in the thin film phase (Figures S20–S22 and Table S7) and in dispersed phase. The aim was to fully characterise the excited states away from the 0–40 ps range [i. e. instrument response] for these new hybrid materials, and compare these with the features of the free porphyrin molecules, in the solution phase as well as aggregate states in this film. As the lifetime average is above 1 ns for all of the free base porphyrins studied, as well as their emerging hybrid systems involving GO and rGO, the most appropriate ultrafast experiments carried out in solution and in thin film were those using FLIM with 810 nm excitation ^.[26,31]^ The solution/dispersion TCSPC measurements presented in ESI are inclusive of the control experiments involving coronene as a small‐molecular model for rGO.

**Figure 8:**
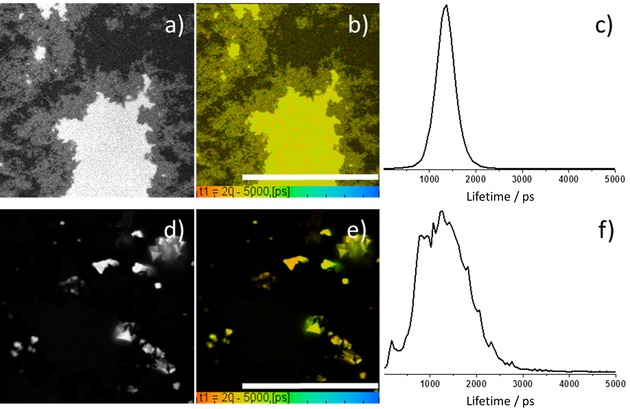
Single‐photon FLIM of a thin film obtained by drop casting of a 1 mg/mL dispersion of nanohybrids in CHCl_3_ : EtOH 1 : 1 onto borosilicate glass, (a) **1**@rGO, intensity image, λ_ex_=405 nm; (b) lifetime mapping of τ_1_; (c) corresponding fluorescence lifetime τ_1_ distribution curve; (d) **2**@rGO, intensity image, λ_ex_=405 nm; (e) lifetime mapping of τ_1_; (f) corresponding fluorescence lifetime τ_1_ distribution. Scale bars: 20 μm.

Thin films were obtained by drop‐casting and drying chloroform : ethanol 1 : 1 suspensions of hybrids **1**–**3**@rGO onto borosilicate glass coverslip and FLIM data was compared to that obtained in the dispersed phase by Time‐Correlated Single Photon Counting (TCSPC) lifetime decays of those frameworks dispersed in CHCl_3_ (data given in ESI). For the thin film phase investigations the single photon FLIM imaging (Figure 8, **ESI**), at 405 nm excitation resulted in an average fluorescence emission lifetime of 2400±100 ps for **1**@rGO. The lifetime emission mapping of **2**@rGO and **3**@rGO respectively also showed rather narrow distributed lifetimes, e. g. the corresponding lifetime distribution curves showed a shorter lifetime in compound **2**@rGO, 1730±100 ps and longer lifetime in **3**@rGO, 2700±100 ps.

**Figure 9:**
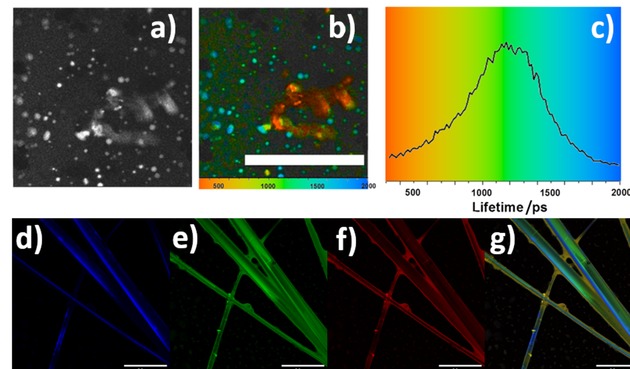
Two‐photon fluorescence lifetime imaging microscopy of drop‐casted **3**@rGO composite, λ_ex_=810 nm (a–c). (a) Intensity image; (b) average fluorescence lifetime mapping. Scale bar is 100 μm; (c) corresponding average fluorescence lifetime distribution curve. Super‐resolution Airyscan confocal microscopy images of **3**@coronene hybrids (d–g). (d) Blue channel (λ_exc_ 405 nm); (e) green channel (λ_exc_ 488 nm); (f) red channel (λ_exc_ 561 nm); (g) overlay of blue, green and red channels.

However, when 2‐photon excitation was used for fluorescence spectroscopy investigations, the fitting on multiple exponentials pictured more complex scenarios (**ESI**) where short‐lived intensely emissive species assignable to supramolecular aggregates could also be imaged, additionally to the longer‐lived emissive hybrids. Two‐photon fluorescence lifetime imaging microscopy (FLIM) investigations of the solid hybrid material formed between compound **3** and rGO in thin film was determined using 810 nm (ca. 0.1 mW). Lifetime data could be fitted to a two‐component (t_1_ and t_2_) with χ^2^ ca 1.02. In Figure 9a–c), average lifetime is t_m_=1200±300 ps, and a range of lifetimes (of similar magnitudes) were generally consistent to those found for porphyrins **1**–**3** and their relevant rGO composites (see Figures S20–S22 and Table S7 in ESI).

The corresponding values of the t_1_ and t_2_ together with their amplitudes are given in ESI. Observations from two‐photon TCSPC lifetime decays in the dispersed phase (measured in CHCl_3_, 1 mg/ml conc. and using 810 nm 2‐photon excitation) are consistent throughout: in each case, a short‐lived highly fluorescent component of ca. 410 ps could also be identified (ESI). There are also, as expected, marked differences in lifetime determinations between solvent‐dispersed scenario and thin film measurements which highlight the solvent influence in the electronic properties of the composites within these porous materials.

The three types of functional porphyrins used present subtly different interactions with rGO which result in different local electron interactions/transitions within the system and different degrees of quenching, but which are also solvent‐mediated in each case. This is consistent with similar observations in Zn‐porphyrins@GO and NDI@thermally reduced GO where flat, π‐conjugated molecules were found to self‐organise in stacks onto the graphene layers in thin film.^34^ An inspection of the 2P TCSPC data for **1**–**3**@rGOs and a comparison with the corresponding spectroscopy of free base porphyrins **1**–**3**, revealed that the characteristic emission of an intact porphyrin core appears to be preserved. Further studies in our laboratories are in progress to identify whether or not the original J‐stacking of porphyrins is altered following solvothermal treatment as it has been reported for alternative “guest”‐like molecules.^13^ Fluorescence lifetime measurement evidences show the nature of the porphyrin molecules frameworks to be maintained for **1**–**3** after the solvothermal treatment, and no clear evidence regarding the deprotection of the thioactetate groups in Compound **3** has been found upon a close inspection of the current XPS data.

To further understand the microscopic morphologies of the hybrids, super‐resolution confocal fluorescence microscopy images using the Airyscan technology of **1**–**3**@coronene complexes were recorded and compared to the data obtained from 2P FLIM for **2**@coronene. There are close similarities in the FLIM behaviour in the nano‐crystalline phase of **3**@coronene and 3@rGO both in terms of emissive properties and 2P FLIM parameters. Interestingly, in terms of surface self‐assembly behaviour, the AFM imaging of **1**–**3**@coronene matches to a high degree of confidence the behaviour observed by super‐resolution spectroscopy as well as SEM imaging (ESI). These experiments aimed to fully characterise the hybrid **2**@coronene in thin film phase by FLIM and correlated microscopic techniques and are given in Supporting information (Figure S29). In the case of **2**@coronene, it was advantageous to use two‐photon excitation as this minimises the effects of laser quenching and significant photo‐bleaching in the bulk material compared to one‐photon excitation. All FLIM parameters for the rGO‐based materials are in alignment with the **2**@coronene model. We have recently reported that these changes in the lifetime, particularly the presence of a lower lifetime component account for a photo‐induced excited state electron transfer which occurs when aromatic chromophores and planar carbon materials are closely bound generating donor‐acceptor systems.^30^ Such results and variation in fluorescence lifetime decay suggest that different functional units at the periphery of the porphyrin core not only affect the distribution of the composite pore sizes but also give rise to photo‐induced excited state electron transfers where the porphyrin acts as a donor and the reduced graphene oxide is the acceptor.^52^, ^53^, ^54^, ^55^

**Figure 10:**
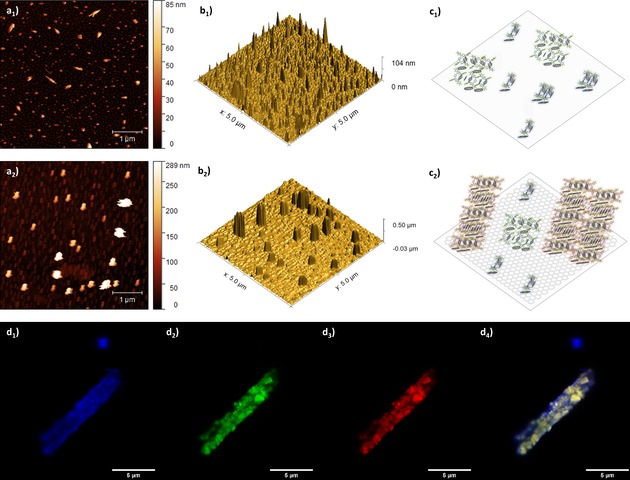
Investigations into the self‐assembly processes within **2**@coronene by AFM and super resolution confocal microscopy. a_1–2_) tapping mode AFM image of **2**@coronene on mica (a_1_) and HOPG substrates (a_2_). b_1–2_) Corresponding 3D images of a_1_ and a_2_. c_1–2_) Proposed supramolecular aggregation models of **2**@coronene on mica (c_1_) or HOPG (c_2_) substrates. d_1–4_) Super resolution Airyscan confocal microscopy images, d_1_) blue channel (λ_exc_ 405 nm); d_2_) green channel (λ_exc_ 488 nm); d_3_) red channel (λ_exc_ 561 nm); d_4_) overlay of blue, green and red channels.

The aromatic stacking of porphyrins and coronene was further investigated by AFM by varying the nature of the substrate used to record the TM AFM of the samples. We hypothesised that by using highly oriented pyrolytic graphite (HOPG) surfaces the stacking of porphyrin‐coronene hybrids would be favoured with respect to mica surfaces. The samples were prepared by immersion of the corresponding substrates in 0.5 mM solutions of porphyrin@coronene samples in chloroform/toluene (1 : 1). The tapping mode AFM images for **2**@coronene (Figure 10) showed two main groups of nanostructures in mica surfaces: small particles with sizes in the 2–4 nm range and stacks with heights in the 40–50 nm range. Interestingly, in the case of HOPG samples, a distribution of nanostructures with 10–15 nm heights was observed in the background while a second group of stacked hybrids with larger sizes, reaching 200–400 nm, was observed. This is an indicative of the preferential controlled stacking of the aromatic porphyrin‐coronene hybrids in HOPG surfaces with respect to mica surfaces. The stacking behaviour was also present in the **2**@coronene crystals grown by solvent diffusion and studied by super resolution confocal microscopy (Figure 10, d_1–4_) showing differential fluorescence behaviour from the starting materials, differentiated domains and structure sizes in the micrometre scale. The π‐electron density available in the free base porphyrins of choice affects the binding ability in the non‐covalently interactions that occur by π‐π stacking with rGO and thus imparts control of the morphologies, in turn being able to mediate hybrid framework porosities.

## 3 Conclusion

We discovered a new route to controlled porosity of new 3D structures by addressing molecular level stacking interactions. New 3D fluorogenic hierarchical architectures having an ability to vary their pore volumes in presence of organic solvents emerged. To the best of our knowledge, this is an unprecedented feature in hybrid self‐assembled materials: this in situ method generates rGO hierarchical architectures with a variety of morphologies giving rise to localised fluorescence, electronic and energy transfer properties, of relevance to future functional energy materials and electronic materials design.

Functionalised free‐base porphyrin molecules intercalated within rGO or coronene layers hold these together by strong aromatic interactions giving rise to flexible 3D networks capable of accommodating solvent molecules through increasing the distance between the aromatic planes by acting as the ‘molecular glue’. These porous materials appear to be responsive to the presence of the organic solvent, and the nature of the molecular glue present in terms of their local optical properties. We have synthesised a set of three different flexible 3D rGO‐based network that are capable to adapt solvent molecules through increasing the distance between GO planes. Single X‐ray diffraction using coronene as a model showed the aromatic stacking of porphyrin, coronene and toluene molecules. Spectroscopic measurements in solution and in the thin film phase indicate that the electronic properties in these porous networks are influenced by the choice of porphyrin “guest” as well as by the presence of the organic solvent. Thus, the pore size of the resulting framework can be tuned by a simple supramolecular self‐assembly route using different free‐base porphyrins without introducing high‐energy consumption techniques such as high vacuum systems or extensive covalent chemistry modifications. Energy transfer between porphyrins and graphene oxide/rGO was predicted by DFT and determined by UV‐vis and fluorescence spectroscopies, also by single/multiphoton fluorescence lifetime spectroscopies in the dispersed phase and thin film. Experimental and modelling (semi‐empirical and DFT level calculations) investigations indicate that strong non‐covalent interactions between the porphyrins and rGO are responsible for the fine tuning of pore size and flexible 3D‐network obtained and this lead to the rational design a palette of new 3D carbon‐based functional materials with electronic properties thus far unexplored. This design and synthesis principle having supramolecular polymerisation processes at the core allows the extension of an accurate control of the 3D framework pore size from Å level (as in the case of covalent organic frameworks) to nanometre and/or micron scale due to the supramolecular frameworks constructs, of relevance to new materials with applications as energy materials.

## Experimental Section

### General Synthesis of Graphene and Porphyrin 3D Framework

The synthesis of 3‐dimensional porphyrin@rGO frameworks was carried out by the one‐step solvothermal reactions. As a general procedure, the porphyrin was dissolved in chloroform (1 mM) and then mixed with an ethanol dispersion of GO (0.5 mg/mL) in a 1 : 1 volume ratio. To keep a batch‐to‐batch consistency across all experiments, the reduction of graphene oxide was dispersed in the same solvent (1 : 1 ethanol and chloroform). The dispersed mixture in a Teflon liner was heated in an autoclave at 200 °C for 24 hours. After the solvothermal process was completed, the autoclave was left to return to room temperature under air.

### Materials Characterisation

A comprehensive examination of the obtained products was conducted using a broad set of characterisation techniques. TEM images were obtained with Gatan Dualvison digital camera on a JEOL 1200EXII transmission electron microscope coupled with energy‐dispersive X‐ray spectroscopy (point resolution, 0.16 nm). Raman spectroscopy was carried out on a Renishaw inVia Raman spectroscopy. The specimens were either in solid state or dispersed in pure water (MilliQ) or water:ethanol 1 : 1 mixture. During the measurements, the carbon nanomaterials samples were deposited on an aluminium substrate. The laser wavelength was set at 514 nm. At least ten accumulations were generally acquired in Raman spectroscopy measurements, the beam was focused in at least three different positions across the specimen and these spectra were averaged to obtain batch‐representative peaks and most reliable results. Atomic force microscopy measurements were carried out with a Digital Instruments Multimode Atomic Force Microscope with IIIa controller. Thermogravimetric (TGA) measurements were carried out on a Polymer Laboratories STA 1500 Simultaneous Analysis System. Samples for TGA analysis were placed in a ceramic crucible which was heated from room temperature up to 900 °C at a rate of 2 °C per minute, with data points collected every 2 seconds under dynamic N2 flow (industrial grade, flow rate 100 ml min^−1^).

### X‐ray Photoelectron Spectroscopy

X‐ray photoelectron spectroscopy (XPS) was used to characterise the chemical composition of the samples. XPS spectra were acquired in an ultrahigh vacuum (UHV) chamber with a base pressure of 1×10^−9^ mbar using a hemispherical electron energy analyser (SPECS Phoibos 150 spectrometer) and a monochromatic AlKα X‐ray source (1489.74 eV). XPS spectra were recorded at the normal emission take‐off angle, using an energy step of 0.05 eV and a pass‐energy of 10 eV for high resolution data, which provides an overall instrumental peak broadening of 0.4 eV.^56^, ^57^ A suitable number of scans (depending on the core level) were acquired so that the statistic and error, i. e. the signal‐to‐noise ratio, was good enough to ensure that there are no additional changes in the spectrum during acquisition. Carbon and hydroxyl (OH) species were also detected as surface contaminants and the signal form adventitious carbon at 284.6 eV was used for energy calibration. Data processing was performed using CasaXPS software. The specific surface area was determined by the Brunauer‐Emmett‐Telle (BET) method together with nitrogen adsorption/desorption isotherms were carried out on an ASAP 2020‐Micromeritics (Norcross, GA, USA) at 77 K. Samples were degassed at 120 °C during 48 h before analysis.

### Single Crystals X‐ray Diffraction

Single crystals suitable for X‐ray diffraction experiment for complex **2**@coronene were grown from toluene over several weeks. Experimental details relating to the single‐crystal X‐ray crystallographic study are summarised in Tables S5–S7. Data for complex **2**@coronene were acquired using an Agilent Supernova Dual diffractometer equipped with an EosS2 CCD plate detector using a mirror monochromator (Cu Kα radiation, λ=1.54184 Å). All structures were solved with SHELXT and refined by a full‐matrix least‐squares refinement based on F^2^ (Shelxl‐2014/7).^58^ All non‐hydrogen atoms were refined with anisotropic displacement parameters. C‐H hydrogen atom were placed onto calculated positions and refined riding on their parent atom. All heteroatom hydrogen atoms have been in the difference Fourier map and were refined. Additional programmes used for analysing data and their graphical manipulation included: SHELXle^59^, ORTEP‐3 for windows.^60^ Additionally, for space group determination, structure solution and full‐matrix least‐squares refinement the WINGX‐v2014 suite of programs was used.^61^ All non‐hydrogen atoms were refined with anisotropic displacement parameters. C−H hydrogen atom were placed onto calculated positions and refined riding on their parent atom. All hetero atom hydrogen atoms have been located in the difference Fourier map and were refined freely. The program MERCURY (CCDC)^62^ was employed for the graphics used in this publication. Data was deposited to Cambridge Structural Database, CCDC/CSD Deposition Number 1922138.

### Two Photon Fluorescence Spectroscopy and Fluorescence Lifetime Imaging

Two‐photon excitation experiments were performed at the Rutherford Appleton Laboratory following the methodology described by Botchway et al.^27^, ^34^, ^63^ and used in an identical setup with that described in ref. [27]. A mode locked Mira Ti‐sapphire laser (from Coherent Lasers Ltd, USA), generating 180 fs pulses at 75 MHz and emitting light at a wavelength of 710–970 nm was used for the 2‐photon excitation. The laser was pumped by a solid state continuous wave 532 nm laser (Verdi V18, Coherent Laser Ltd), with the oscillator fundamental output of 810±2 nm. The laser beam was focused to a diffraction limited spot through a water immersion ultraviolet corrected objective (from Nikon VC x60, NA1.2) and specimens illuminated at the microscope stage of a custom‐modified Nikon TE2000‐U with UV transmitting optics. The focused laser spot was raster scanned using an XY galvanometer (GSI Lumonics). Fluorescence emission was collected and passed through a coloured glass (BG39) filter and detected by fast microchannel plate photomultiplier tube used as the detector (R3809‐U, Hamamatsu, Japan). These were linked in a correlative way via a TCSPC PC module SPC830. Lifetime calculations were obtained using SPCImage analysis software (Becker and Hickl, Germany).

### DFT Calculations

The molecular level DFT calculations were based on first‐principles density functional theory together with dispersion correction (DFT+D). The VASP code^64^, ^65^ was used to solve the standard Kohn‐Sham equations using plane wave basis sets with the energy cut‐off of 500 eV. A single k‐point was used in all geometry optimisations. Densities of States (DOS) were plotted using 2×2×2 Monkhorst‐Pack^66^ *k* point mesh. For the exchange correlation energy term the Generalised Gradient Approximation (GGA) was used in a form of Perdew, Burke and Ernzerhof (PBE).^67^ Structure optimisations were performed using a conjugate gradient algorithm and the forces on the atoms were smaller than 0.001 eV/Å. A cubic supercell with a length of 35 Å was used in all calculations to make sure that adjacent images do not interact. We define the binding energy by the following equation.

$$E_{bind}=E_{PorPhy\_Coro}-\;E_{Porphy}-E_{Coro}$$

where $E_{Porphy}$ is the total energy of the isolated porphyrin molecule, $E_{Coro}$ is the total energy of the coronene and $E_{PorPhy\_Coro}$ is the total energy of the porphyrin binding the surface of a coronene molecule. Here we include van der Waals (vdW) forces by using pair‐wise force field as implemented by Grimme *et al*.^68^ in the VASP package. The optimisation of the structures was carried out using MMFF94 Force Field in Avogadro (v 1.2.0). The electrostatic surface potentials were calculated in MarvinSpace (v 16.9.5.0).

## Conflict of interest

The authors declare no conflict of interest.

## Supporting information

As a service to our authors and readers, this journal provides supporting information supplied by the authors. Such materials are peer reviewed and may be re‐organized for online delivery, but are not copy‐edited or typeset. Technical support issues arising from supporting information (other than missing files) should be addressed to the authors.

Supplementary
