# Supplementary material for: Directed Molecular Stacking for Engineered Fluorescent Three‐Dimensional Reduced Graphene Oxide and Coronene Frameworks
Source: ChemistryOpen. 2019 Dec 4;8(12):1383–98. doi: 10.1002/open.201900310 (PMC6892451; doi:10.1002/open.201900310)
Supplement: Supplementary file 1 — Supplementary [file OPEN-8-1383-s001.pdf]

Open Access
ChemistryOPEN

Supporting Information

© Copyright Wiley-VCH Verlag GmbH & Co. KGaA, 69451 Weinheim, 2019

Directed Molecular Stacking for Engineered Fluorescent Three-Dimensional Reduced Graphene Oxide and Coronene Frameworks

Boyang Mao,* Fernando Cortezon-Tamarit+, Haobo Ge+, Navaratnarajah Kuganathan+, Vincenzo Mirabello, Francisco J. Palomares, Gabriele Kociok-Köhn, Stanley W. Botchway, David G. Calatayud,* and Sofia I. Pascu*© 2019 The Authors. Published by Wiley-VCH Verlag GmbH & Co. KGaA.
This is an open access article under the terms of the Creative Commons Attribution License, which permits use, distribution and reproduction in any medium, provided the original work is properly cited.This article is part of a Special Collection dedicated to Functional Supramolecular Systems

Supporting Information

Table of contents

1. **Experimental Section** 2
2. **UV-vis titration experiments** 6
3. **2D fluorescence spectroscopy** 10
4. **BET experimental data** 13
5. **Single crystal X-ray diffraction crystallography and associated comparisons** 14
6. **Raman spectroscopy** 18
7. ***TGA experiments*** 19
8. **XPS experiments** 22
9. **Confocal microscopy, 1P and 2P fluorescence spectroscopy TCSPC and FLIM** 23
10. **AFM microscopy on mica and on HOPG supports** 36
11. **Scanning electron microscopy** 41
13. **DFT Calculations data:** 46
14. **Semiempirical modelling** 51

1. Experimental Section

Synthesis of Graphene Oxide: Graphene oxide (GO) sheets were exfoliated from graphite oxide using an adapted procedure from the Hummers method,[1] whereby the graphite oxide was generated from natural graphite powder (SP-1 grade 325 mesh, Bay Carbon Inc.). Powdered flake graphite (10 g) and sodium nitrate (5 g) were stirred in 230 mL concentrated sulphuric acid in a 1.5 L beaker. This was then cooled down in an ice bath and maintained at 0 °C temperature. Under stirring, potassium permanganate (30 g) was then added into this mixture. The rate of addition was controlled carefully so that the temperature of the mixture did not exceed 20 °C. The reaction was then removed from the ice bath and the temperature was allowed to rise to 35 °C. The mixture was then stirred at room temperature for a further 30 min. Distilled H_2O (460 mL) was slowly added into the mixture, and it was observed that the temperature of the mixture increased, effervescence occurred. Then, the mixture was diluted to ca. 1.4 L with distilled water. An aqueous solution of H_2O_2 (3%) was slowly added into the mixture to remove excess permanganate and manganese dioxide. Afterwards, the colour of the suspension became bright yellow. The suspension was filtered while still warm, and then rinsed with excess warm water. The solid was collected onto a Cyclopore® membrane followed by dispersion in 3.2 L distilled water. A two week dialysis process was introduced to remove any remaining salts. Afterwards, the suspension was filtered again and rinsed with distilled water (Millipore water). The solid fractions were collected from the filtering membrane and the solid products kept at 60 °C in a furnace overnight to make sure the graphite oxide was fully dried. For the preparation of graphene oxide, an ethanol dispersion was prepared from the dried product and sonicated in an ultrasonic bath (40 kHz) for 3 h, following a 6 × 30 min sonication program with 10 min intervals between each session. The suspension was then centrifuged at 3000 rpm for 30 min. The supernatant was collected and separated from the precipitate. It remained free of precipitation upon standing over one month period only when a microfiltration was performed, and only the clear supernatant was used in the experiments. This lengthy procedure was deemed necessary, and repeated several times to secure batch-to-batch consistency of the resulted GO material. This was tested by IR spectroscopy and compared to the corresponding spectra of graphite and graphite oxide. [1]

Synthesis of the self-assembled reduced graphene oxide hydrogel by hydrothermal treatment

The self-assembled and reduced graphene oxide hydrogel was carried out to build up the 3D structure from the 2D precursors. The modification and functionalisation of graphene oxide had been widely established.[2] The hydrothermal reaction of graphene oxide was applied to build

up a 3D network structure. Three-dimensional network structures obtained a list of beneficial properties including: macroscale size, high accessible surface area, less restacking, highly-interconnected microstructure, high strength and flexibility, fast ion transport and electron conductivity.[3] The synthesis was performed by adding 0.5 mg/mL graphene oxide water solution into an autoclave. The reaction was heated at 200 °C for 24 hours and the pressure generated within the autoclave ramped up to 1800 psi. The relatively high temperature and high pressure contributed to reduce and exfoliate the graphene oxide. This process was also repeated under similar conditions whereby EtOH, or $CHCl_3$: EtOH (1:1) were used instead of H_2O. After the solvothermal process was completed, the autoclave was allowed to return to room temperature in air.

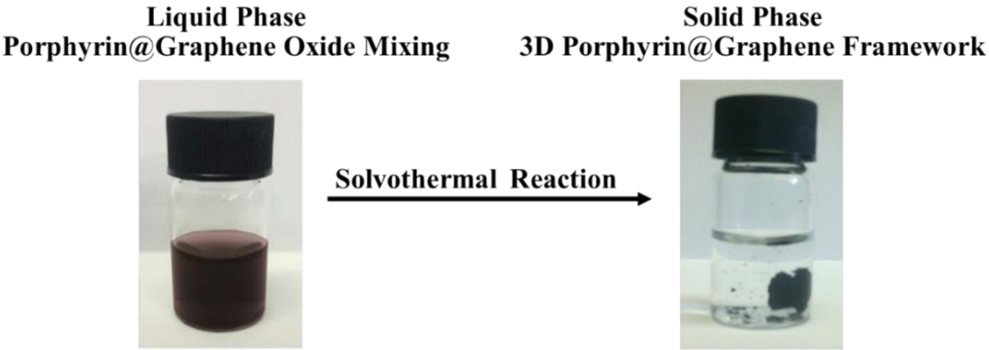

Figure S1. Images of the dispersion of **3** and GO mixing phase and of solid phase 3D cellular porphyrin and rGO framework after the solvothermal reaction in $CHCl_3$: EtOH (1:1).

Materials characterisation details: A comprehensive examination of the obtained products was conducted using a broad set of characterisation techniques. TEM images were obtained with Gatan Dualvison digital camera on a JEOL 1200EXII transmission electron microscope coupled with energy-dispersive X-ray spectroscopy (point resolution, 0.16 nm). Raman spectroscopy was carried out on a Renishaw inVia Raman spectroscopy. The specimens were either in solid state or dispersed in pure water (MilliQ) or water:ethanol 1:1 mixture. During the measurements, the carbon nanomaterials samples were deposited on an aluminium substrate. The laser wavelength was set at 514 nm. At least ten accumulations were generally acquired in Raman spectroscopy measurements, the beam was focused in at least three different positions across the specimen and these spectra were averaged to obtain batch-representative peaks and most reliable results. Atomic force microscopy measurements were carried out with a Digital Instruments Multimode Atomic Force Microscope with IIIa controller. Thermogravimetric (TGA) measurements were carried out on a Polymer Laboratories STA 1500 Simultaneous Analysis System. Samples for TGA analysis were placed in a ceramic

crucible which was heated from room temperature up to 900°C at a rate of 2°C per minute, with data points collected every 2 seconds under dynamic nitrogen flow (industrial grade, flow rate 100 ml min^{-1}).

X-ray photoelectron spectroscopy: X-ray photoelectron spectroscopy (XPS) was used to characterise the chemical composition of the samples. XPS spectra were acquired in an ultrahigh vacuum (UHV) chamber with a base pressure of 1 x 10^{-9} mbar using a hemispherical electron energy analyser (SPECS Phoibos 150 spectrometer) and a monochromatic AlKα X-ray source (1489.74 eV). XPS spectra were recorded at the normal emission take-off angle, using an energy step of 0.05 eV and a pass-energy of 10 eV for high resolution data, which provides an overall instrumental peak broadening of 0.4 eV.[4, 5] A suitable number of scans (depending on the core level) were acquired so that the statistic and error, i.e. the signal-to-noise ratio, was good enough to ensure that there are no additional changes in the spectrum during acquisition. Carbon and hydroxyl (OH) species were also detected as surface contaminants and the signal form adventitious carbon at 284.6 eV was used for energy calibration. Data processing was performed using CasaXPS software.
The specific surface area was determined by the Brunauer-Emmett-Telle (BET) method together with nitrogen adsorption/desorption isotherms were carried out on an ASAP 2020-Micromeritics (Norcross, GA, USA) at 77 K. Samples were degassed at 120 °C during 48 h before analysis.

*Single crystals X-ray diffraction for compound **2**@coronene.*
Single crystals suitable for X-ray diffraction experiment for complex **2**@coronene were grown from toluene over several weeks. Experimental details relating to the single-crystal X-ray crystallographic study are summarised in Tables **S5** – **S7**. Data for complex **2**@coronene were acquired using an Agilent Supernova Dual diffractometer equipped with an EosS2 CCD plate detector using a mirror monochromator (Cu Kα radiation, λ = 1.54184 Å). All structures were solved with SHELXT and refined by a full-matrix least-squares refinement based on F^2 (Shelxl-2014/7).[6] All non-hydrogen atoms were refined with anisotropic displacement parameters. C-H hydrogen atom were placed onto calculated positions and refined riding on their parent atom. All heteroatom hydrogen atoms have been in the difference Fourier map and were refined. Additional programmes used for analysing data and their graphical manipulation included: SHELXle[7] , ORTEP-3 for windows[8]. Additionally, for space group determination, structure solution and full-matrix least-squares refinement the WINGX-v2014 suite of programs was

used.[9] All non-hydrogen atoms were refined with anisotropic displacement parameters. C-H hydrogen atom were placed onto calculated positions and refined riding on their parent atom. All hetero atom hydrogen atoms have been located in the difference Fourier map and were refined freely. The program MERCURY (CCDC)[10] was employed for the graphics used in this publication. Data was deposited to Cambridge Structural Database, CCDC/CSD Deposition Number 1922138.

Two Photon Fluorescence Spectroscopy and Fluorescence Lifetime Imaging. Two-photon excitation experiments were performed at the Rutherford Appleton Laboratory following the methodology described by Botchway et al.[11-13] and used in an identical setup with that described in ref. [11]. A mode locked Mira Ti-sapphire laser (from Coherent Lasers Ltd, USA), generating 180 fs pulses at 75 MHz and emitting light at a wavelength of 710–970 nm was used for the 2-photon excitation. The laser was pumped by a solid state continuous wave 532 nm laser (Verdi V18, Coherent Laser Ltd), with the oscillator fundamental output of 810 ± 2 nm. The laser beam was focused to a diffraction limited spot through a water immersion ultraviolet corrected objective (from Nikon VC x60, NA1.2) and specimens illuminated at the microscope stage of a custom-modified Nikon TE2000-U with UV transmitting optics. The focused laser spot was raster scanned using an XY galvanometer (GSI Lumonics). Fluorescence emission was collected and passed through a coloured glass (BG39) filter and detected by fast microchannel plate photomultiplier tube used as the detector (R3809-U, Hamamatsu, Japan). These were linked in a correlative way via a TCSPC PC module SPC830. Lifetime calculations were obtained using SPCImage analysis software (Becker and Hickl, Germany).

Super-resolution confocal microscopy was acquired in a Zeiss LSM880 microscope with Airyscan using 405, 488 and 561 nm excitation lasers.

Fluorescence spectroscopy measurements in single photon excitation modes were carried out on a Perkin Elmer Luminescence spectrophotometer LS 55. UV–vis spectroscopy was carried out in a Perkin-Elmer Lambda 35 spectrometer.

2. UV-vis titration experiments

The supramolecular interactions between compounds **1**, **2** and **3** with coronene were studied by UV-vis titrations in a chloroform/toluene (1:1) solvent system. Coronene was selected as a simple model for 2D carbon nanomaterials as reported before and the titrations were carried out as previously described.[11-13] Coronene was progressively added from a stock solution of porphyrin (5 µM)–coronene (50 µM) of porphyrin (5 µM) in $CHCl_3$/toluene (1:1) in order to keep the concentration of porphyrin constant throughout the experiment. The UV-vis spectra were measured in the 250 – 900 nm range, after every addition / aliquot removal sequence. The characteristic porphyrin band at ca. 410 nm was monitored and an increase in intensity observed upon addition of coronene.

The absorption values of this band at ca. 410 nm were recorded and analysed using a MATLAB fitting program developed by Thordarson.[14] The results indicate that the interaction between porphyrins **1** – **3** and coronene can be fitted following a 1:1 equilibrium model and that association constants are in the 10^5 M^{-1} order of magnitude. Selected statistical parameters such as standard error of estimated data (SEy) or the covariance of the fit (Cov_f) as well as the association constants ($K_{a1:1}$) are summarised in Table S1. The UV-vis spectra acquired during the titrations and corresponding binding isotherms for ΔA values can be seen in Figures S2-4.

Table S1. Estimated binding constants and corresponding statistical parameters (SEy, standard error of estimated data and Cov_f, covariance of the fit) for the titrations of compounds **1** – **3** with coronene.

	**Compound 1**	**Compound 2**	**Compound 3**
$K_{a1:1}$ (M^{-1})	$9.7x10^4$	$3.1x10^5$	$5.4x10^5$
SEy	0.00435	0.00205	0.00110
Cov_f	0.00608	0.00852	0.0133

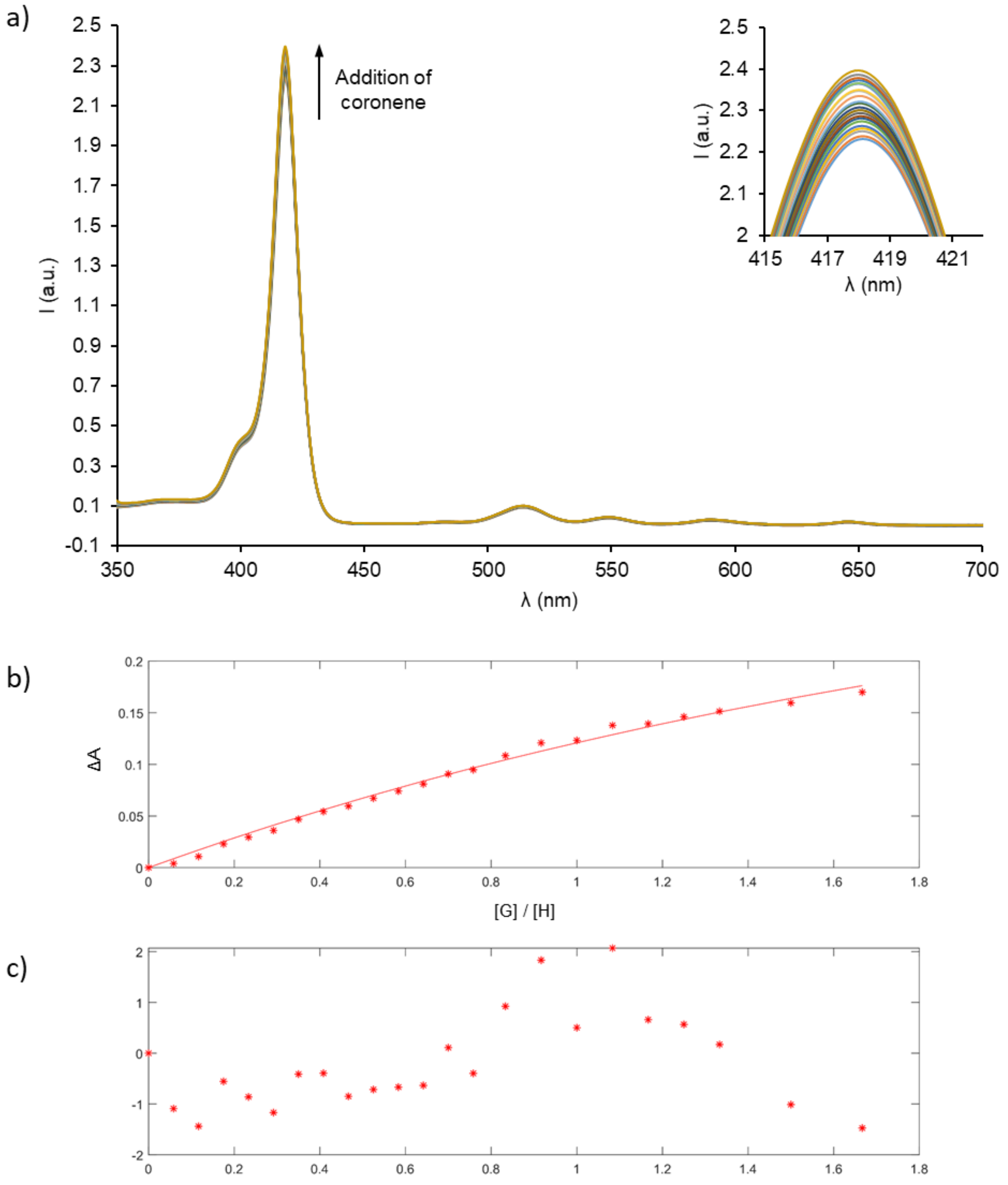

Figure S2. a) UV-vis titration of coronene into a solution of compound **1** including and inset with a magnification of the 418 nm band. b) Representation of the absorption change of the 418 nm band versus the equivalents of coronene added (red asterisks) as well as the 1:1 binding isotherm fitting (red line). c) Standard deviation of absorption values.

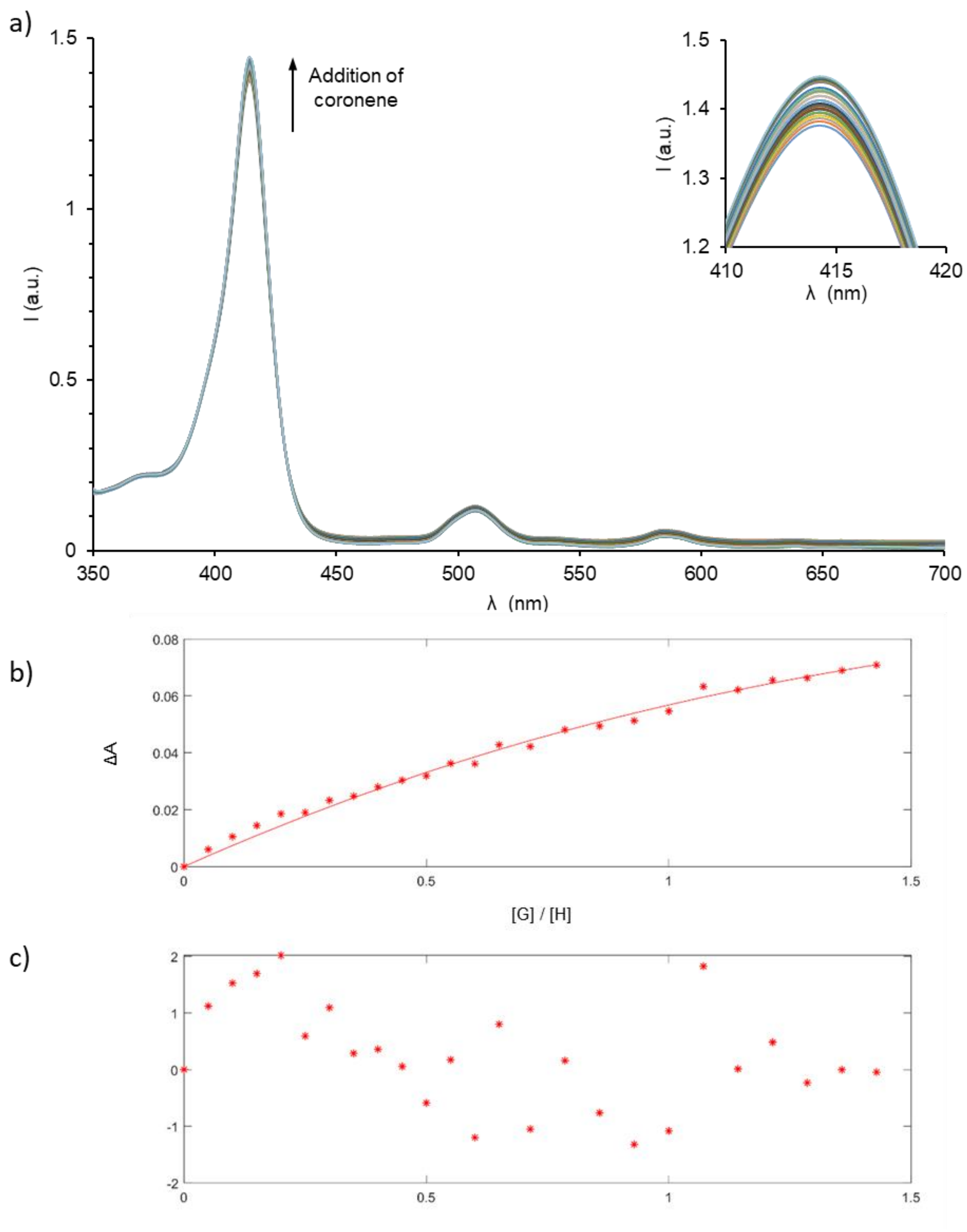

Figure S3. a) UV-vis titration of coronene into a solution of compound **2** including and inset with a magnification of the 414 nm band. b) Representation of the absorption change of the 414 nm band versus the equivalents of coronene added (red asterisks) as well as the 1:1 binding isotherm fitting (red line). c) Standard deviation of absorption values.

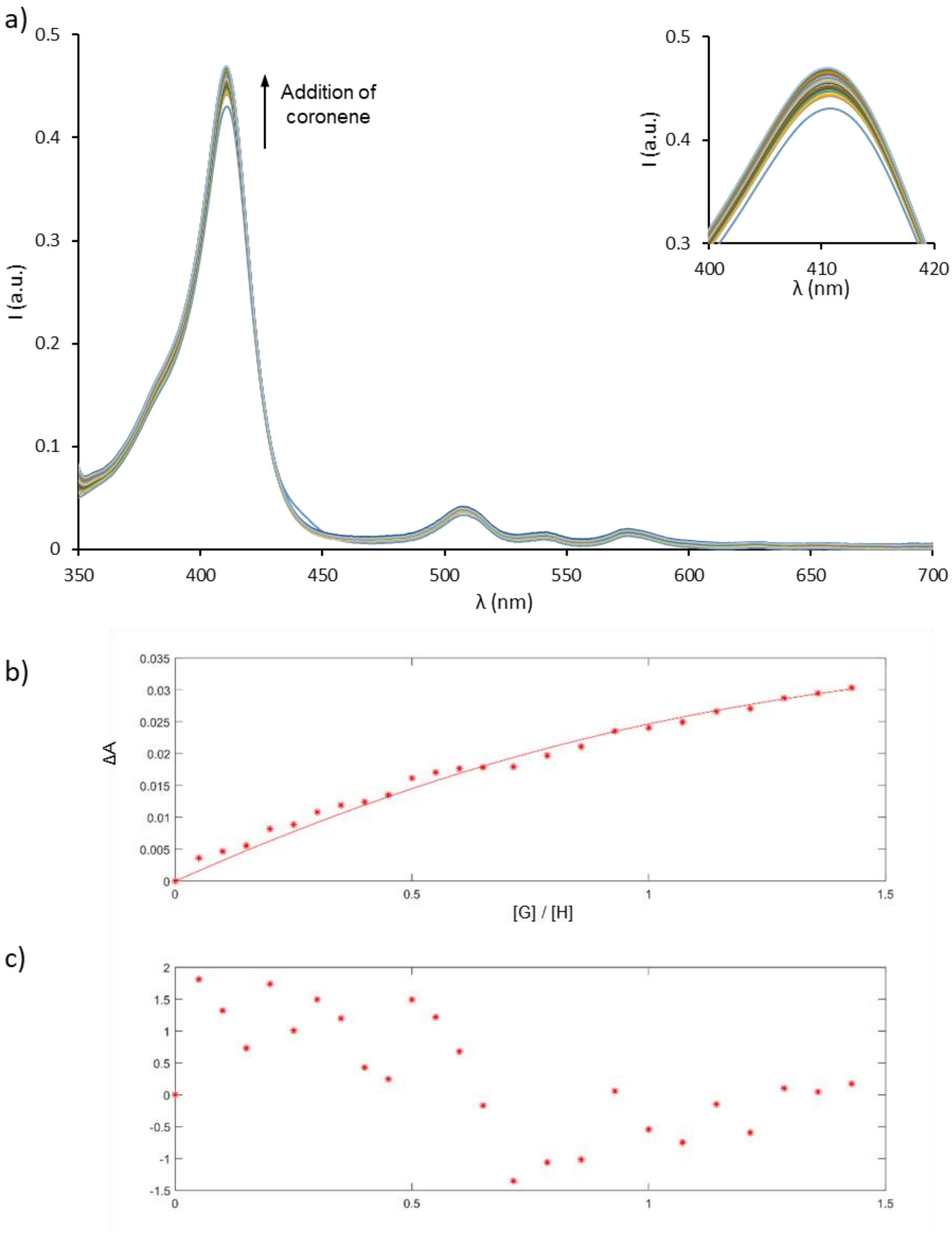

Figure S4. a) UV-vis titration of coronene into a solution of compound **3** including and inset with a magnification of the 411 nm band. b) Representation of the absorption change of the 411 nm band versus the equivalents of coronene added (red asterisks) as well as the 1:1 binding isotherm fitting (red line). c) Standard deviation of absorption values.

3. 2D fluorescence spectroscopy

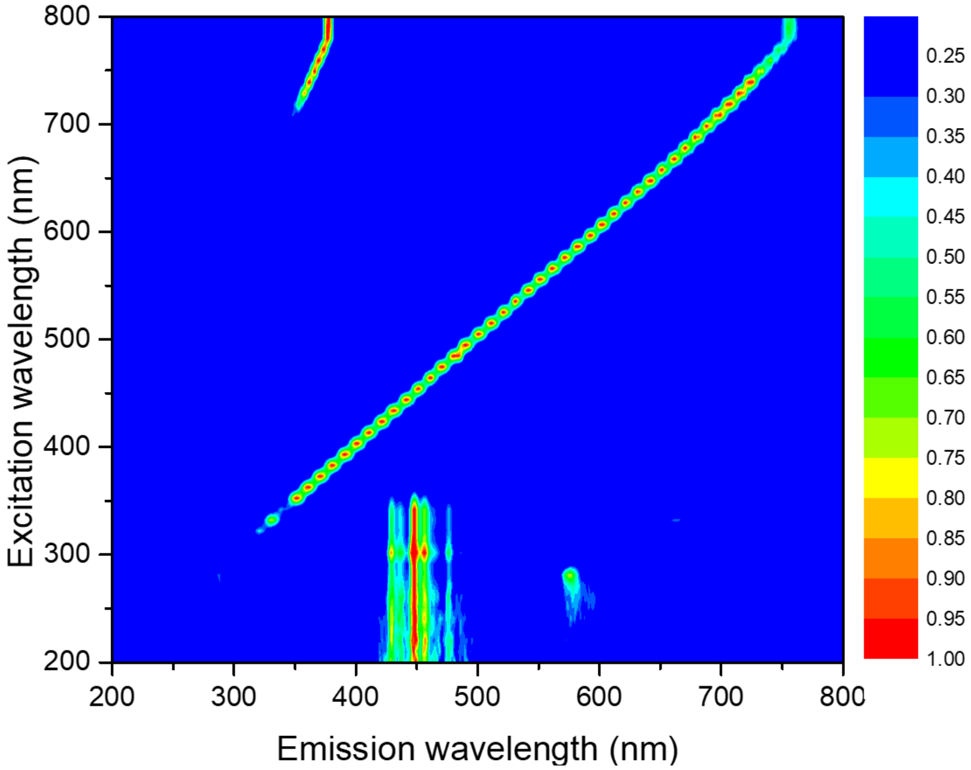

Figure S5. 2D excitation/emission mapping of coronene (5 μM) in $CHCl_3$: toluene (1:1).

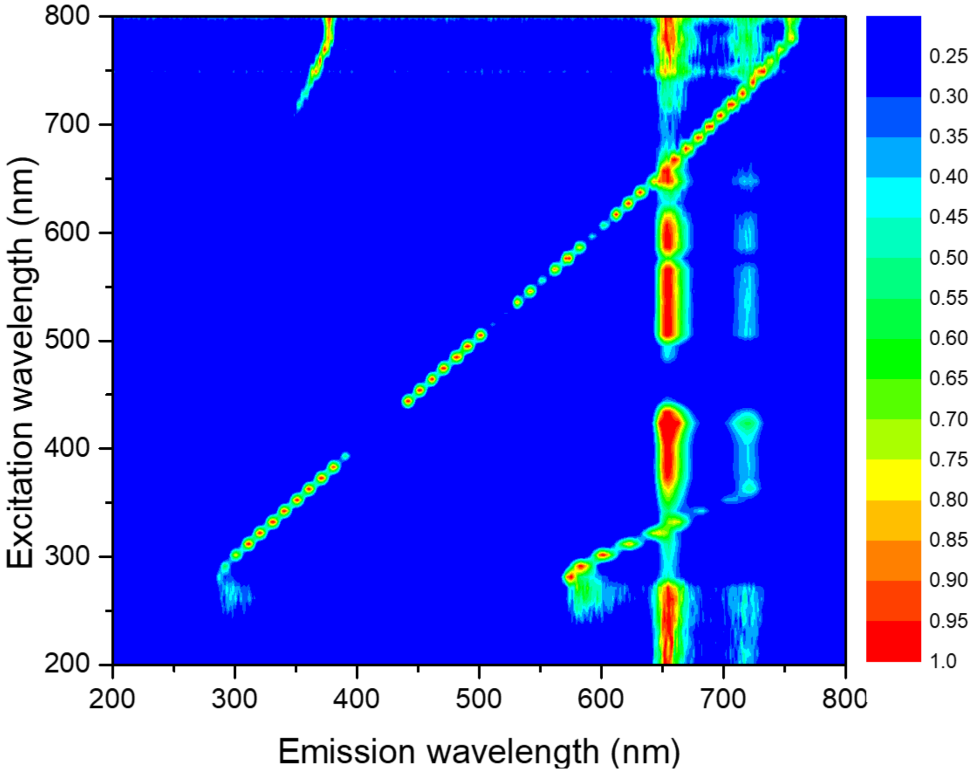

Figure S6. 2D excitation/emission mapping of **1** (5 μM) in $CHCl_3$: toluene (1:1).

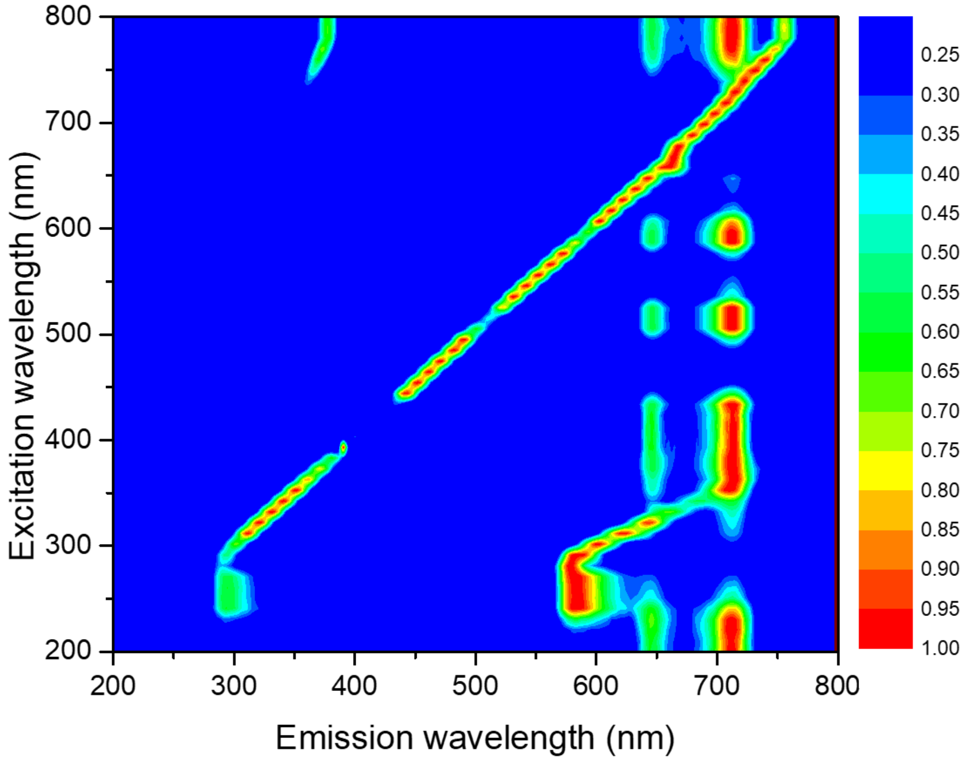

Figure S7. 2D excitation/emission mapping of **2** (5 µM) in $CHCl_3$: toluene (1:1).

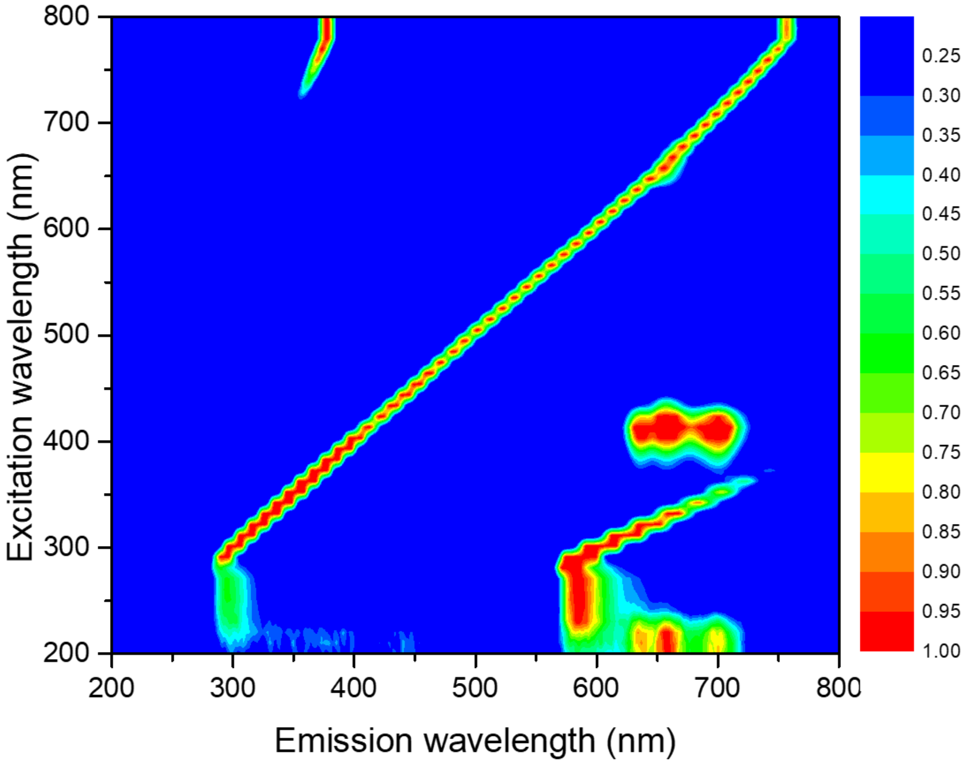

Figure S8. 2D excitation/emission mapping of **3** (5 µM) in $CHCl_3$: toluene (1:1).

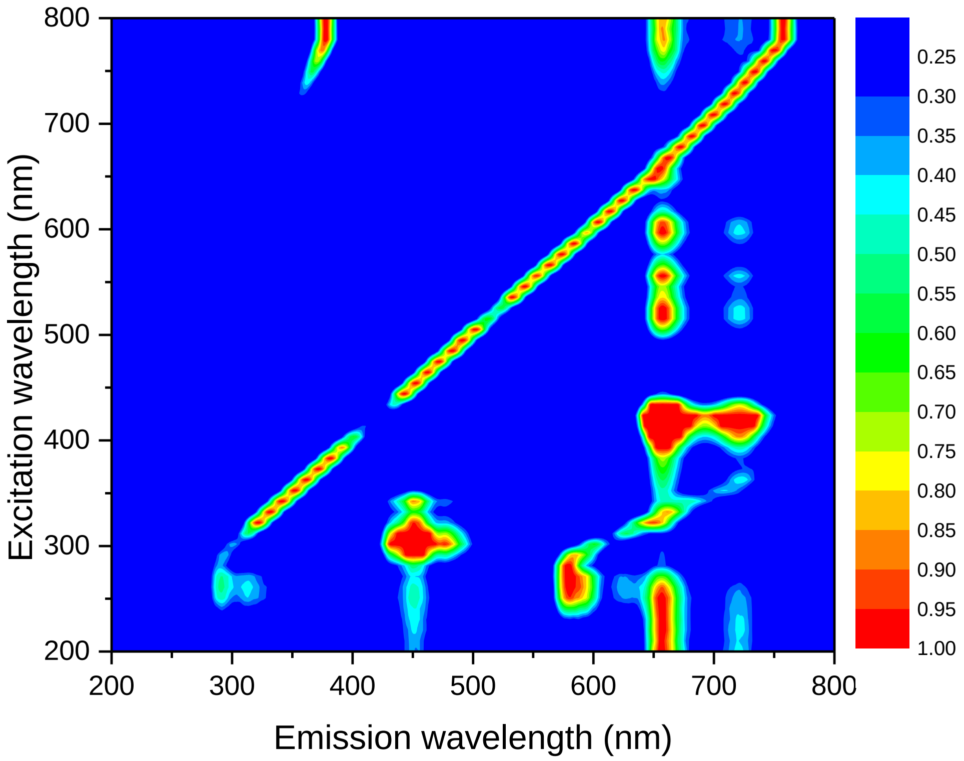

Figure S9. 2D excitation/emission mapping of **1**@coronene solution (5 µM) in $CHCl_3$: toluene (1:1).

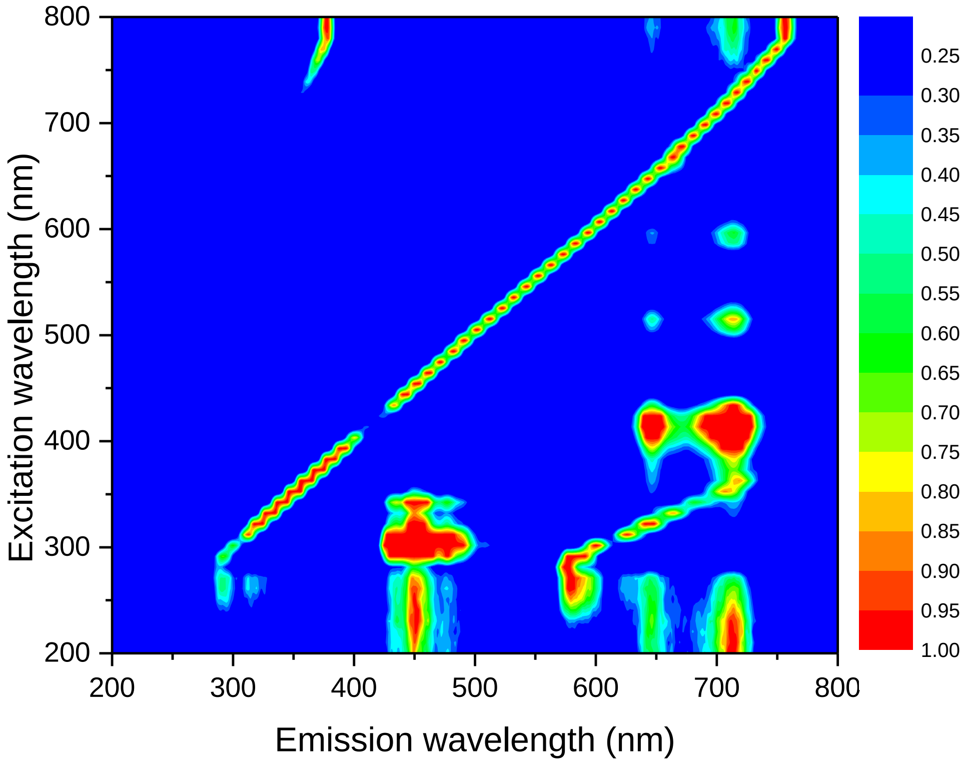

Figure S10. 2D excitation/emission mapping of **2**@coronene solution (5 µM) in $CHCl_3$: toluene (1:1).

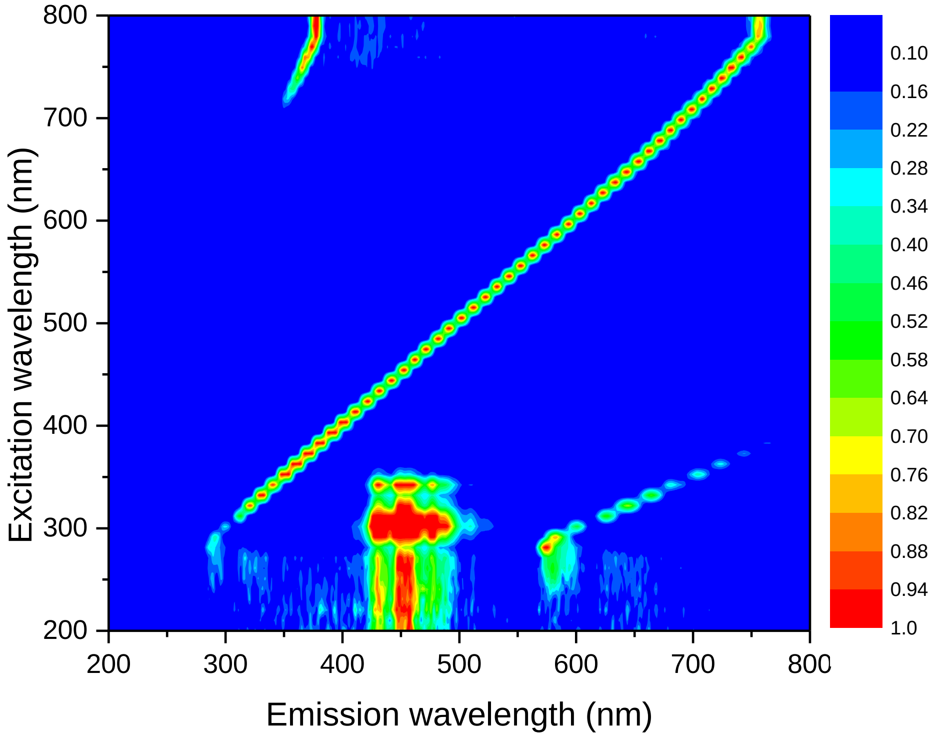

Figure S11. 2D excitation/emission mapping of **3**@coronene solution (5 µM) in $CHCl_3$ / toluene (1:1).

4. BET experimental data

Table S2. Specific surface area of the prepared samples and corresponding pore size (BJH desorption).

**Sample**	**Area Se (m^2/g)**	**Pore Size (des) (Å)**
**1**@rGO	211	33.1
**2**@rGO	46	45.7
**3**@rGO	58	31.7

5. Single crystal X-ray diffraction crystallography and associated comparisons

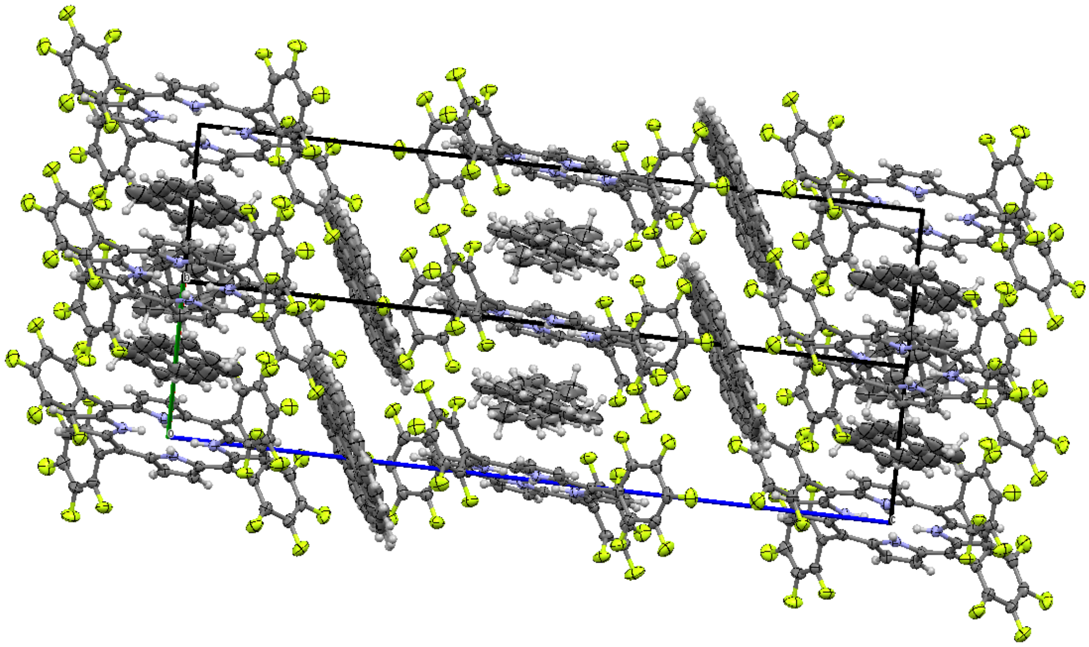

Figure S12. Unit cell for porphyrin **2**@coronene hybrid, showing that the π-π interaction is established between the porphyrin core and the solvating toluene molecules are forming porphyrin-toluene stacking columns and no porphyrin-porphyrin stacking contacts are observed.

Table S3. Crystal data and structure refinement for porphyrin **2**@coronene structure.

Empirical formula	C82 H38 F20 N4	
Formula weight	1459.16	
Temperature	150(2) K	
Wavelength	1.54184 Å	
Crystal system	Monoclinic	
Space group	C2/c	
Unit cell dimensions	a = 7.05350(10) Å	α= 90°.
	b = 28.0761(3) Å	β= 90.9390(10)°.
	c = 31.7306(4) Å	γ = 90°.
Volume	6282.92(14) Å^3	
Z	4	
Density (calculated)	1.543 Mg/m^3	
Absorption coefficient	1.149 mm^{-1}	
F(000)	2952	
Crystal size	0.254 x 0.119 x 0.015 mm^3	
Theta range for data collection	2.786 to 73.254°.	
Index ranges	-8<=h<=7, -34<=k<=34, -39<=l<=38	
Reflections collected	47582	
Independent reflections	6245 [R(int) = 0.0384]	
Completeness to theta = 67.684°	100.0 %	

Absorption correction	Semi-empirical from equivalents
Max. and min. transmission	1.00000 and 0.68344
Refinement method	Full-matrix least-squares on F2
Data / restraints / parameters	6245 / 20 / 635
Goodness-of-fit on F2	1.078
Final R indices [I>2sigma(I)]	R1 = 0.0418, wR2 = 0.1080
R indices (all data)	R1 = 0.0503, wR2 = 0.1134
Extinction coefficient	n/a
Largest diff. peak and hole	0.315 and -0.239 e.Å^{-3}

Table S4. Selected Bond lengths [Å] for porphyrin **2**.

N(1)-C(4)	1.364(2)	C(9)-C(10)	1.373(3)
N(1)-C(1)	1.366(2)	C(10)-F(10)	1.343(2)
N(1)-H(1)	0.854(18)	C(10)-C(11)	1.378(2)
N(2)-C(12)	1.361(2)	C(11)-F(11)	1.3387(19)
N(2)-C(15)	1.361(2)	C(12)-C(13)	1.451(2)
N(2)-H(2)	0.855(19)	C(13)-C(14)	1.345(2)
C(1)-C(16)#1	1.396(2)	C(14)-C(15)	1.448(2)
C(1)-C(2)	1.437(2)	C(15)-C(16)	1.400(2)
C(2)-C(3)	1.353(2)	C(16)-C(17)	1.499(2)
C(3)-C(4)	1.442(2)	C(17)-C(22)	1.384(2)
C(4)-C(5)	1.399(2)	C(17)-C(18)	1.391(2)
C(5)-C(12)	1.402(2)	C(18)-F(18)	1.335(2)
C(5)-C(6)	1.493(2)	C(18)-C(19)	1.380(2)
C(6)-C(11)	1.385(2)	C(19)-F(19)	1.335(2)
C(6)-C(7)	1.390(2)	C(19)-C(20)	1.372(3)
C(7)-F(7)	1.3381(19)	C(20)-F(20)	1.3372(19)
C(7)-C(8)	1.379(2)	C(20)-C(21)	1.373(3)
C(8)-F(8)	1.336(2)	C(21)-F(21)	1.335(2)
C(8)-C(9)	1.373(3)	C(21)-C(22)	1.384(2)
C(9)-F(9)	1.3363(19)	C(22)-F(22)	1.335(2)

Table S5. Bond angles [°] for porphyrin **2**.

C(4)-N(1)-C(1)	108.29(13)	F(9)-C(9)-C(8)	120.25(17)
C(4)-N(1)-H(1)	125(2)	C(10)-C(9)-C(8)	119.66(15)
C(1)-N(1)-H(1)	127(2)	F(10)-C(10)-C(9)	119.26(16)
C(12)-N(2)-C(15)	107.00(12)	F(10)-C(10)-C(11)	120.99(16)
C(12)-N(2)-H(2)	128(3)	C(9)-C(10)-C(11)	119.75(16)
C(15)-N(2)-H(2)	124(3)	F(11)-C(11)-C(10)	117.71(15)
N(1)-C(1)-C(16)#1	125.67(14)	F(11)-C(11)-C(6)	119.72(14)
N(1)-C(1)-C(2)	108.48(13)	C(10)-C(11)-C(6)	122.57(15)
C(16)#1-C(1)-C(2)	125.85(14)	N(2)-C(12)-C(5)	125.65(14)
C(3)-C(2)-C(1)	107.52(14)	N(2)-C(12)-C(13)	109.62(13)
C(3)-C(2)-H(2A)	126.2	C(5)-C(12)-C(13)	124.72(14)
C(1)-C(2)-H(2A)	126.2	C(14)-C(13)-C(12)	106.73(14)
C(2)-C(3)-C(4)	107.24(14)	C(14)-C(13)-H(13)	126.6
C(2)-C(3)-H(3)	126.4	C(12)-C(13)-H(13)	126.6
C(4)-C(3)-H(3)	126.4	C(13)-C(14)-C(15)	107.11(14)
N(1)-C(4)-C(5)	125.62(14)	C(13)-C(14)-H(14)	126.4
N(1)-C(4)-C(3)	108.47(13)	C(15)-C(14)-H(14)	126.4
C(5)-C(4)-C(3)	125.90(14)	N(2)-C(15)-C(16)	125.80(14)
C(4)-C(5)-C(12)	125.75(14)	N(2)-C(15)-C(14)	109.52(14)
C(4)-C(5)-C(6)	117.29(13)	C(16)-C(15)-C(14)	124.66(14)
C(12)-C(5)-C(6)	116.95(13)	C(1)#1-C(16)-C(15)	126.84(14)
C(11)-C(6)-C(7)	115.85(14)	C(1)#1-C(16)-C(17)	116.94(14)
C(11)-C(6)-C(5)	122.41(14)	C(15)-C(16)-C(17)	116.21(14)
C(7)-C(6)-C(5)	121.70(14)	C(22)-C(17)-C(18)	116.71(15)
F(7)-C(7)-C(8)	117.77(15)	C(22)-C(17)-C(16)	121.93(15)
F(7)-C(7)-C(6)	119.76(14)	C(18)-C(17)-C(16)	121.35(15)
C(8)-C(7)-C(6)	122.46(15)	F(18)-C(18)-C(19)	118.11(16)
F(8)-C(8)-C(9)	119.99(15)	F(18)-C(18)-C(17)	119.88(15)
F(8)-C(8)-C(7)	120.33(16)	C(19)-C(18)-C(17)	122.00(17)
C(9)-C(8)-C(7)	119.66(16)	F(19)-C(19)-C(20)	120.11(16)
F(9)-C(9)-C(10)	120.09(16)	F(19)-C(19)-C(18)	120.35(17)

C(20)-C(19)-C(18)	119.54(17)	F(21)-C(21)-C(22)	120.56(17)
F(20)-C(20)-C(19)	120.17(17)	C(20)-C(21)-C(22)	119.50(16)
F(20)-C(20)-C(21)	119.62(17)	F(22)-C(22)-C(17)	119.76(14)
C(19)-C(20)-C(21)	120.20(15)	F(22)-C(22)-C(21)	118.20(16)
F(21)-C(21)-C(20)	119.93(16)	C(17)-C(22)-C(21)	122.04(16)

Symmetry transformations used to generate equivalent atoms:

#1 -x+1,-y+1,-z+1

6. Raman spectroscopy

Table S6. Raman spectroscopy data in the solid state (λ_{ex} = 514 nm) for reduced graphene oxide and different types of porphyrins composites. (Each spectra average of 3 times at measurement, ±10%); and TGA measurement data.

3D Materials	**D band (cm $^{-1}$)**	**I_D**	**G band (cm $^{-1}$)**	**I_G**	**I_D/I_G**	**decomposition temperature (°C)**
rGO	1348	20641	1592	22518	0.917	600
**1**@rGO	1350	18947	1594	20863	0.908	651
**2**@RGO	1353	10936	1596	11498	0.951	634
**3**@RGO	1350	14784	1596	18316	0.807	Over 700

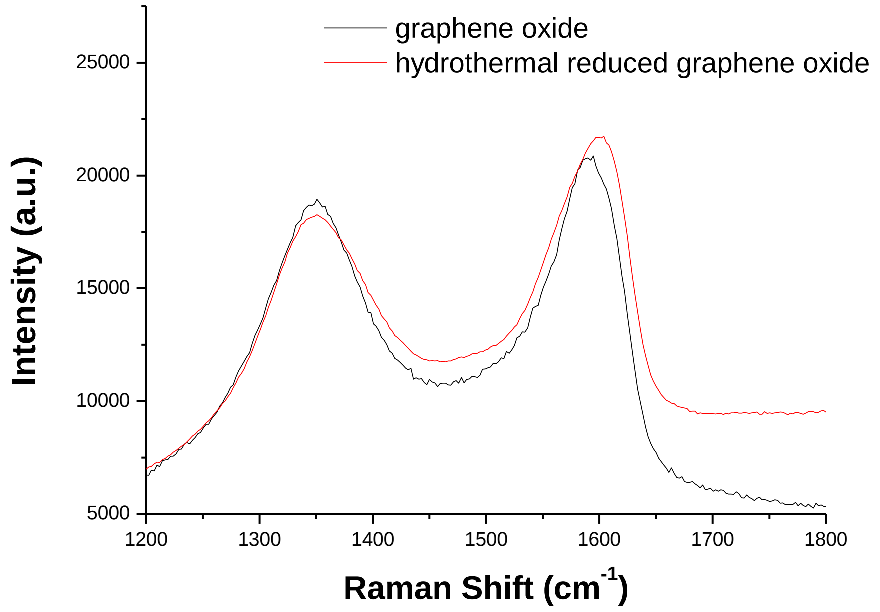

Figure S13. Raman spectrum of hydrothermal reduced graphene oxide.

The rGO 3D framework exhibits a D band at 1348 cm^{-1} and a G band at 1592 cm^{-1} with a 0.917 I_D/I_G ratio, characteristic of the 3D framework expected to be found for the GO.

For the **1**@rGO composite, the D band and G band are at 1350 cm^{-1} and 1594 cm^{-1} respectively, with an I_D/I_G ratio of 0.908. The **2**@rGO composite exhibits a D band at 1353 cm^{-1} and a G band at 1596 cm^{-1}, and an I_D/I_G ratio of 0.951. The **3**@rGO composite presents a D band at

1350 cm^{-1} and a G band at 1596 cm^{-1} with an I_D/I_G ratio of 0.807. All four 3D frameworks exhibited an emerging 2D and D+G peaks at around 2800 cm^{-1}, which indicates quality improvements in terms of defect minimisation with the aromatic network after this solvothermal annealing described, because the C-O, C=O and C(O)OH were likely also partly removed by this *in situ* treatment[15].

7. *TGA experiments*

TGA experiments showed that after the formation of 3D structures, the composite materials present a higher decomposition temperature around 600 °C, **2**@rGO and **1**@rGO decomposed at 634 °C and 651 °C respectively. Although **3**@rGO did not exhibit a clear decomposition temperature, it revealed the strongest thermal stability. TGA results agreed with the hypothesis of interactions between porphyrins and reduced graphene oxide sheets leading to significant structural changes for the 3D frameworks. The reduction of the pore sizes limits the contact with oxygen and as a result the thermal stability of the compounds. The porphyrin molecules are acting like molecular "glue" in the hybrids system, the strong interaction between porphyrins and rGO enhances the inner cross-linking of porous material and may well protect them from decomposing under enhanced temperature in the presence of oxidising conditions.

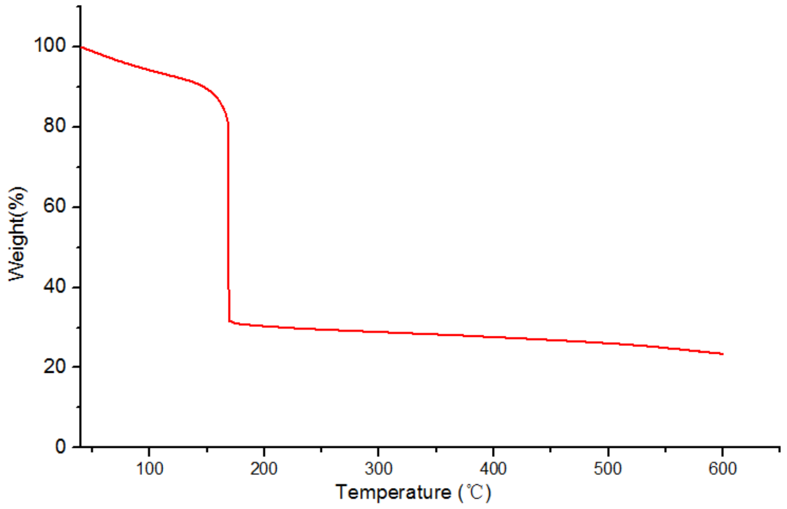

Figure S14. TGA curve obtained for graphene oxide starting material.

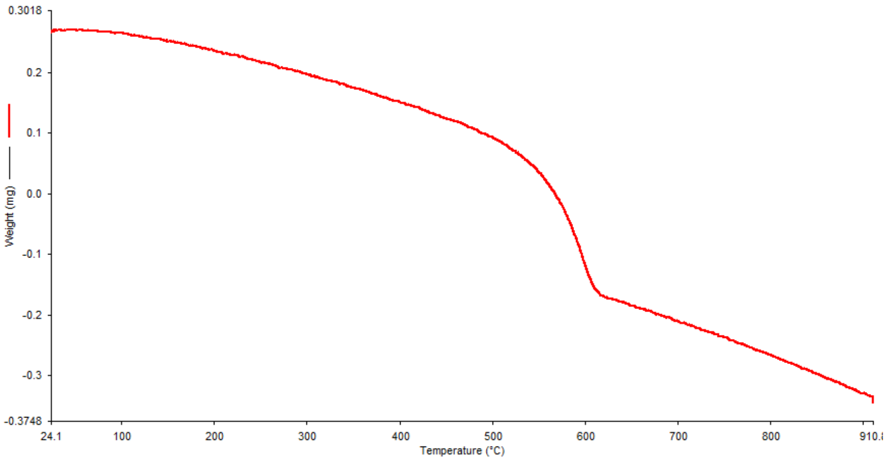

Figure S15. TGA curve obtained hydrothermally reduced graphene oxide.

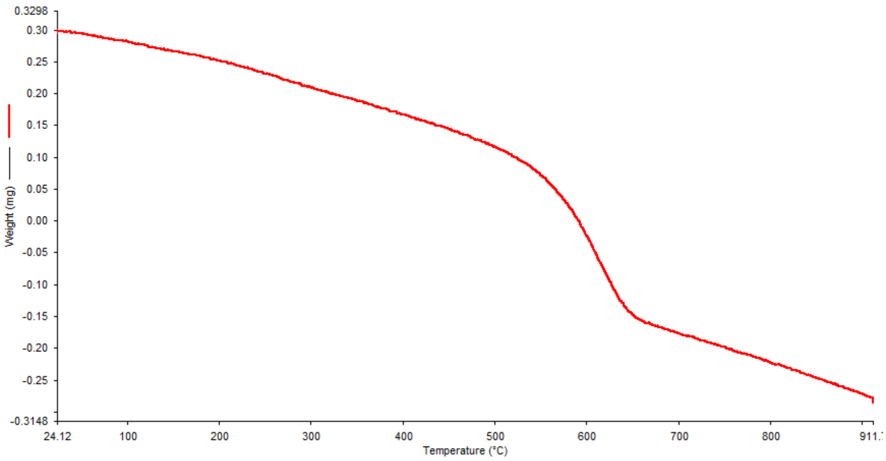

Figure S16. TGA curve obtained **1**@rGO.

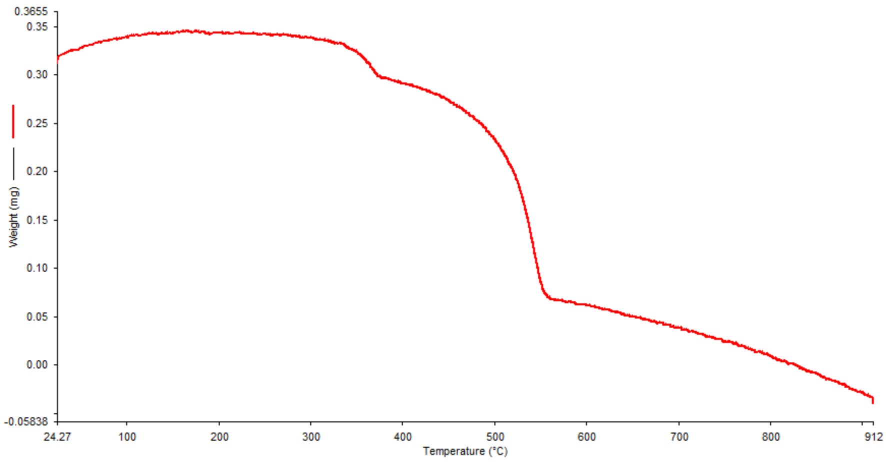

Figure S17. TGA curve obtained **2**@rGO.

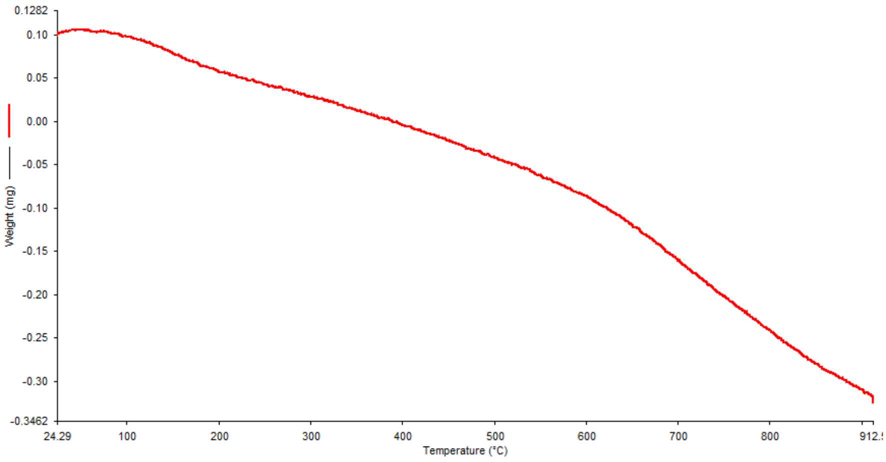

Figure S18. TGA curve obtained **3**@rGO.

8. XPS experiments

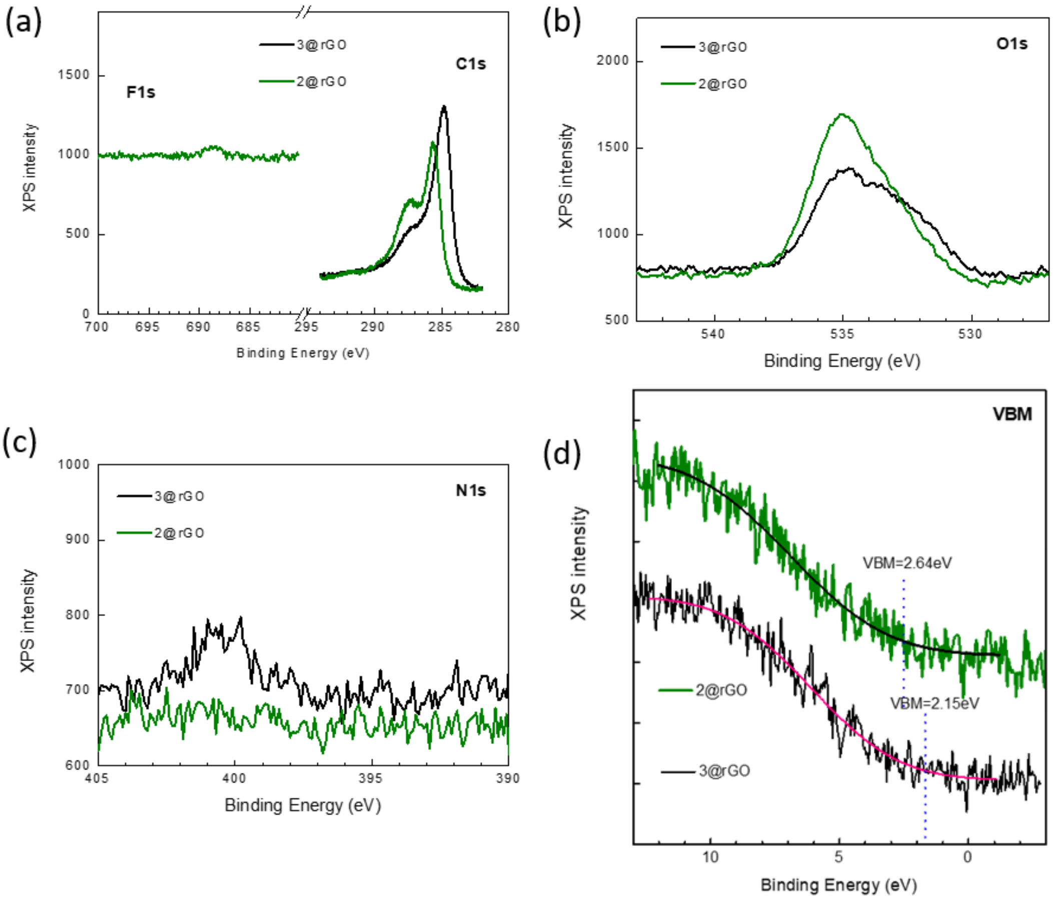

Figure S19. High-resolution XPS spectra corresponding to: (a) C1s and F1s regions for **2**@rGO and **3**@rGO, (b) O1s region for **2**@rGO and **3**@rGO, (c) N1s region for **2**@rGO and **3**@rGO, and (d) VBM region for **2**@rGO and **3**@rGO samples.

Figure S19 shows the XPS data recorded for **2**@rGO with a focus on the O, F, N and C-characteristic regions. These were the only elements observed in the XPS spectra of **2**@rGO, and no obvious impurities were detected. As shown in Figure S19 (a) shows sharp peaks located at around 284.9 eV and 285.7 eV correspond to C=C/C-C in aromatic rings and C-O in hydroxyl and epoxy groups, respectively.[16] The peak corresponding to a binding energy of 287.4 eV corresponds to the carbonyl functional groups. In the same figure, it can be observed the F1s region in which a peak around 688.7 eV is observed for the **2**@rGO sample confirming the presence of the C-F groups from the porphyrin.[17] XPS spectra of the O 1s region were illustrated in Figure S19. (b). Two oxygen signal situated at 535 eV and 532 eV were detected, which can be assigned to C-O-H and C-O respectively.[16] The content of surface hydroxyl oxygen on **2**@rGO is higher than that on **3**@rGO, implying that the physical chemical characteristics of this sample can enhance the surface hydroxyl groups. Finally, the presence of

a signal around 400.5 eV in the N1s region confirms the presence of N in the samples and therefore the formation of porphyrin@rGO materials Figure S19. (c).[16] Figure S19 (d) shows the VBM of **2**@rGO and **3**@rGO, which was measured by XPS, and the VBM is 2.64 eV vs vacuum level for **2**@rGO and lower, ca. 2.15 eV, for **3**@rGO sample. This tendency is in the same line than the values observed in the DOS calculations (**Figure 4 (g-i)**).

9. Confocal microscopy, 1P and 2P fluorescence spectroscopy TCSPC and FLIM

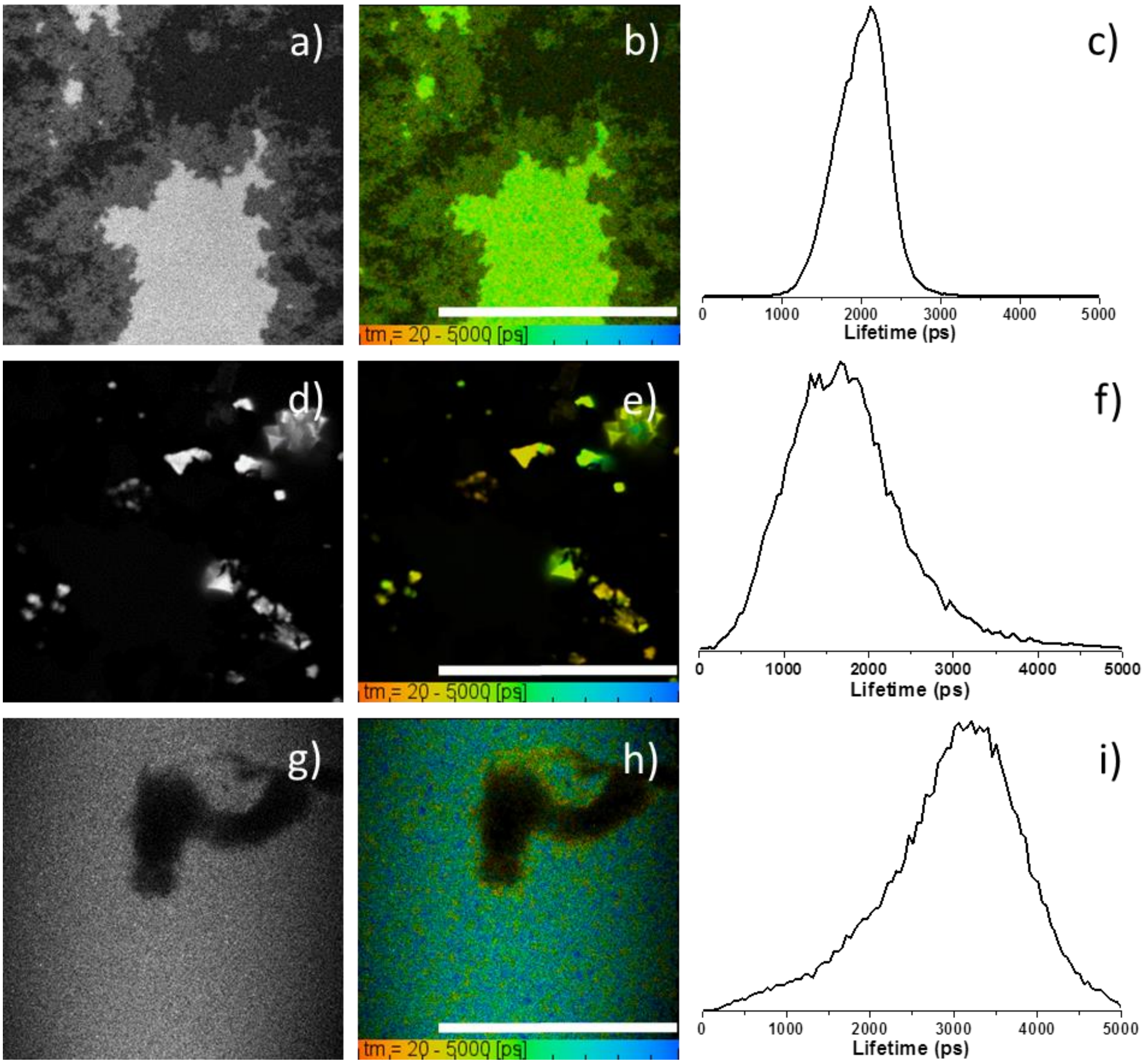

Figure S20. Single-photon FLIM of a thin film obtained by the drop casting of a 1 mg/mL dispersion of nanohybrids in $CHCl_3$:EtOH 1:1 onto borosilicate glass, (a) **1**@rGO composite, intensity image, λ_{ex} = 405 nm; (b) lifetime mapping of τ_m, scale bar: 20 µm; (c) corresponding fluorescence lifetime τ_m distribution curve; (d) **2**@rGO composite, intensity image, λ_{ex} = 405 nm; (e) lifetime mapping of τ_m, scale bar: 20 µm; (f) corresponding fluorescence lifetime τ_m distribution; (g) solid **3**@rGO composite, intensity image, λ_{ex} = 405 nm; (h) lifetime mapping of τ_m, scale bar: 20 µm, (i) corresponding fluorescence lifetime τ_m distribution curve;

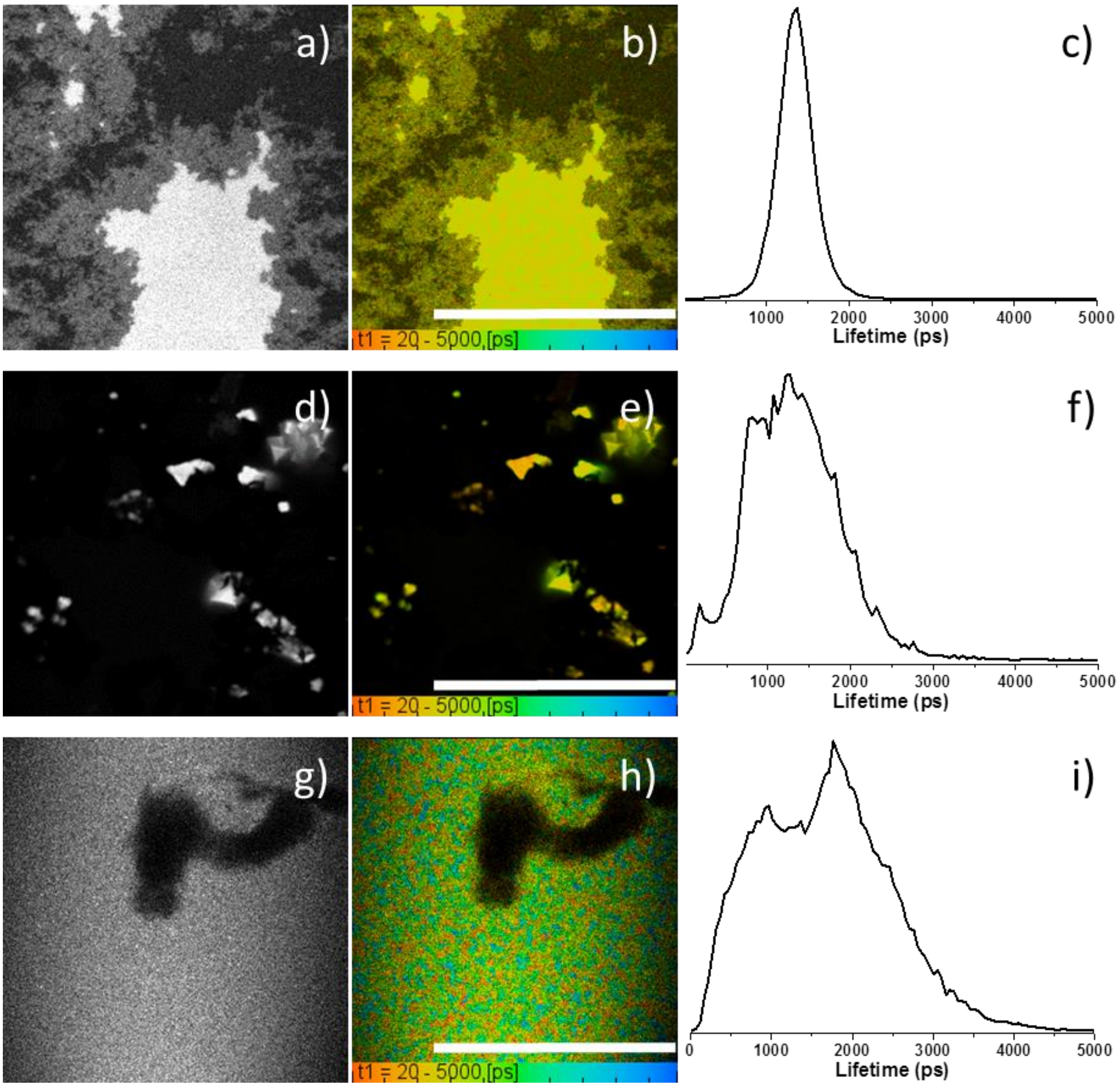

Figure S21. Single-photon FLIM of a thin film obtained by drop casting of a 1 mg/mL dispersion of nanohybrids in $CHCl_3$:EtOH 1:1 onto borosilicate glass, (a) **1**@rGO composite, intensity image, λ_{ex} = 405 nm; (b) lifetime mapping of τ_1, scale bar: 20 µm; (c) corresponding fluorescence lifetime τ_1 distribution curve; (d) solid **2**@rGO composite, intensity image, λ_{ex} = 405 nm; (e) lifetime mapping of τ_1, scale bar: 20 µm; (f) corresponding fluorescence lifetime τ_1 distribution; (g) **3**@rGO composite, intensity image, λ_{ex} = 405 nm; (h) lifetime mapping of τ_1, scale bar: 20 µm (i) corresponding fluorescence lifetime τ_1 distribution curve.

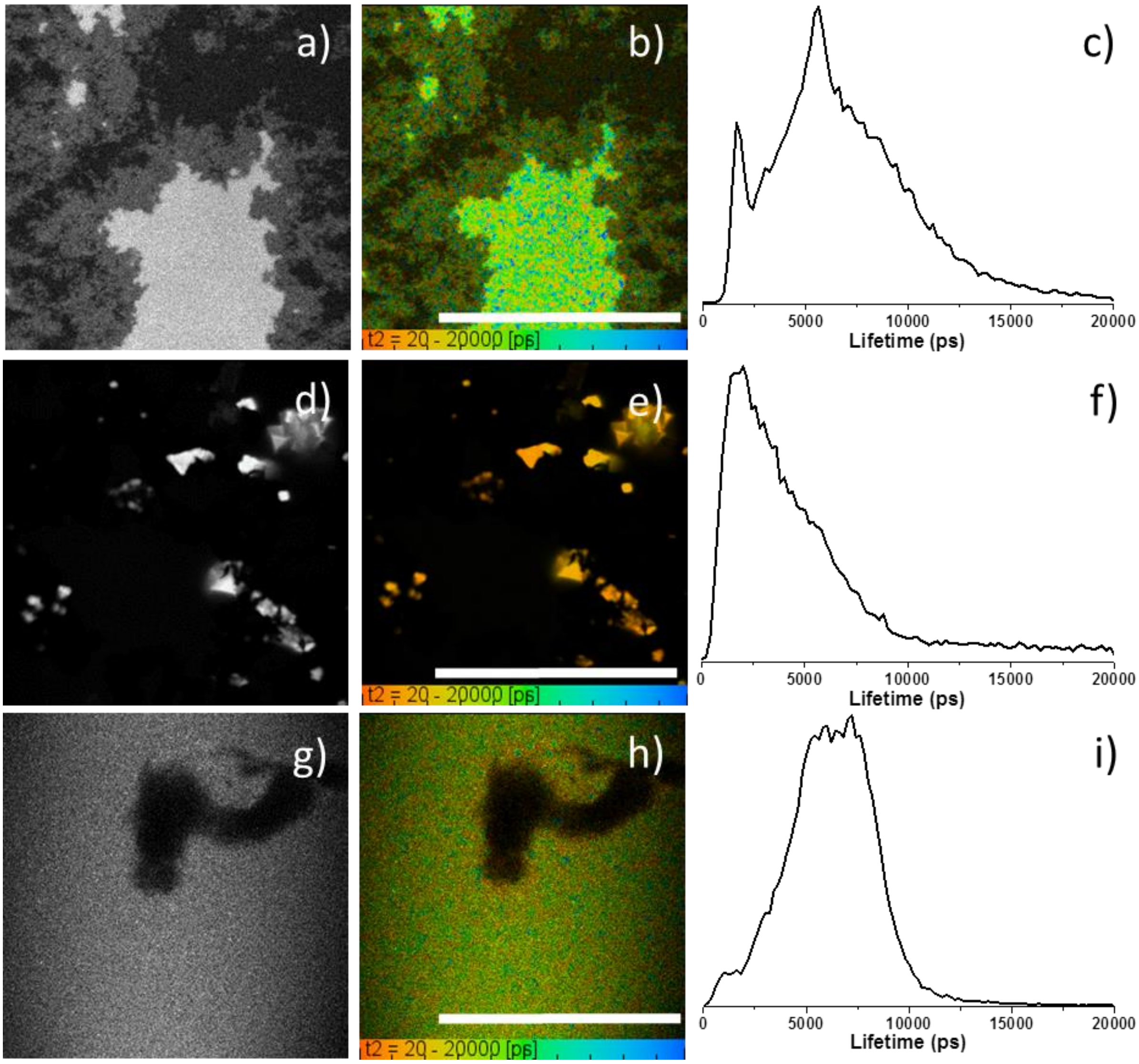

Figure S22. Single-photon FLIM of a thin film obtained by drop casting of a 1 mg/mL dispersion of nanohybrids in $CHCl_3$:EtOH 1:1 onto borosilicate glass, (a) **1**@rGO composite, intensity image, λ_{ex} = 405 nm; (b) lifetime mapping of τ_2, scale bar: 20 µm; (c) corresponding fluorescence lifetime τ_2 distribution curve; (d) **2**@rGO composite, intensity image, λ_{ex} = 405 nm; (e) lifetime mapping of τ_2, scale bar: 20 µm; (f) corresponding fluorescence lifetime τ_2 distribution; (g) solid **3**@rGO composite, intensity image, λ_{ex} = 405 nm; (h) lifetime mapping of τ_2, scale bar: 20 µm (i) corresponding fluorescence lifetime τ_2 distribution curve.

Table S7. Summary data for 1P TCSPC data for pophyrin-rGO hybrids evaluated in a randomly chosen spot in thin film measurements.

	χ^2	t1 (ps)	a1(%)	t2 (ps)	a2(%)	tm (ps)
**1**@rGO	1.38	1190	85.65	8858	14.35	2176
**2**@rGO	1.14	1485	92.04	3947	7.96	1681
**3**@rGO	1.01	1541	71.15	5804	28.85	2771

Figures S20-S22 show respectively the 1P fluorescence lifetime measurements (FLIM) of **1**@rGO, **2**@rGO and **3**@rGO represented in terms of mean lifetime (t_m), short lifetime component (t_1) and long lifetime component (t_2) in the thin film phase. The fluorescence lifetime distribution curves (**c**, **f** and **i**) show broad distribution of mean lifetime of **1**@rGO (2111 ± 1638 ps), **2**@rGO (1668 ± 712 ps) and **3**@rGO (3215 ± 748 ps). Results demonstrate that majority of lifetime measurements of **1**@rGO, **2**@rGO and **3**@rGO appear in the range of 473 – 3749 ps, 956 – 2380 ps and 2467 – 3963 ps respectively. The mean lifetime of chosen spots of **1**@rGO, **2**@rGO and **3**@rGO are 2176 ps, 1681 ps and 2771 ps and they are with their corresponding lifetime range. Compared with the mean lifetime of composites in dispersed form, i.e. **1**@rGO (2400 ± 100 ps), **2**@rGO (1730 ± 712 ps) and **3**@rGO (2700 ± 100 ps), **3**@rGO is the composite most sensitive to the environmental changes and **2**@rGO is the most stable composite to the lifetime change induced by its environment. Similarly, t_1 also is affected remarkably by environmental switch from solution to thin film. The results are increased from sub-nanosecond (459 ps, 405 ps and 403 ps) to nanosecond level and above (1366 ± 227 ps, 1247 ± 608 ps and 1763 ± 1006 ps).

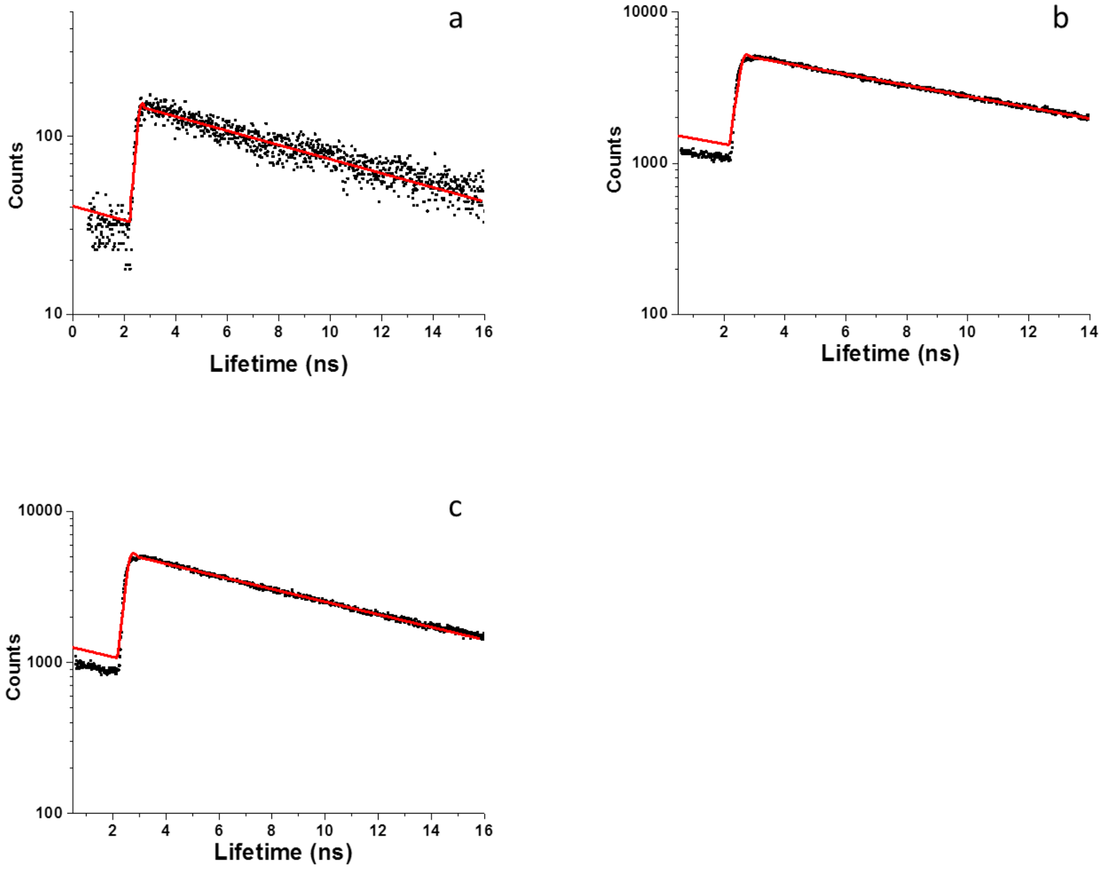

1P TCSPC	χ^2	t_1 (ps)	t_1 (%)	t_2 (ps)	t_2 (%)	t_m (ps)
Dichlorobenzene	1.24	9648	33.15	11553	66.85	10921.82
DMF: toluene	3.69	11898	100	-	-	11898
EtOH: Chloroform	4.64	10349	100	-	-	10349

Figure S23. Single photon TCSPC spectrum of free base porphyrin **3** in different solvent environments, a) dichlorobenzene, b) DMF: toluene (1:1) and c) EtOH: Chloroform (1:1) (405 nm excitation)

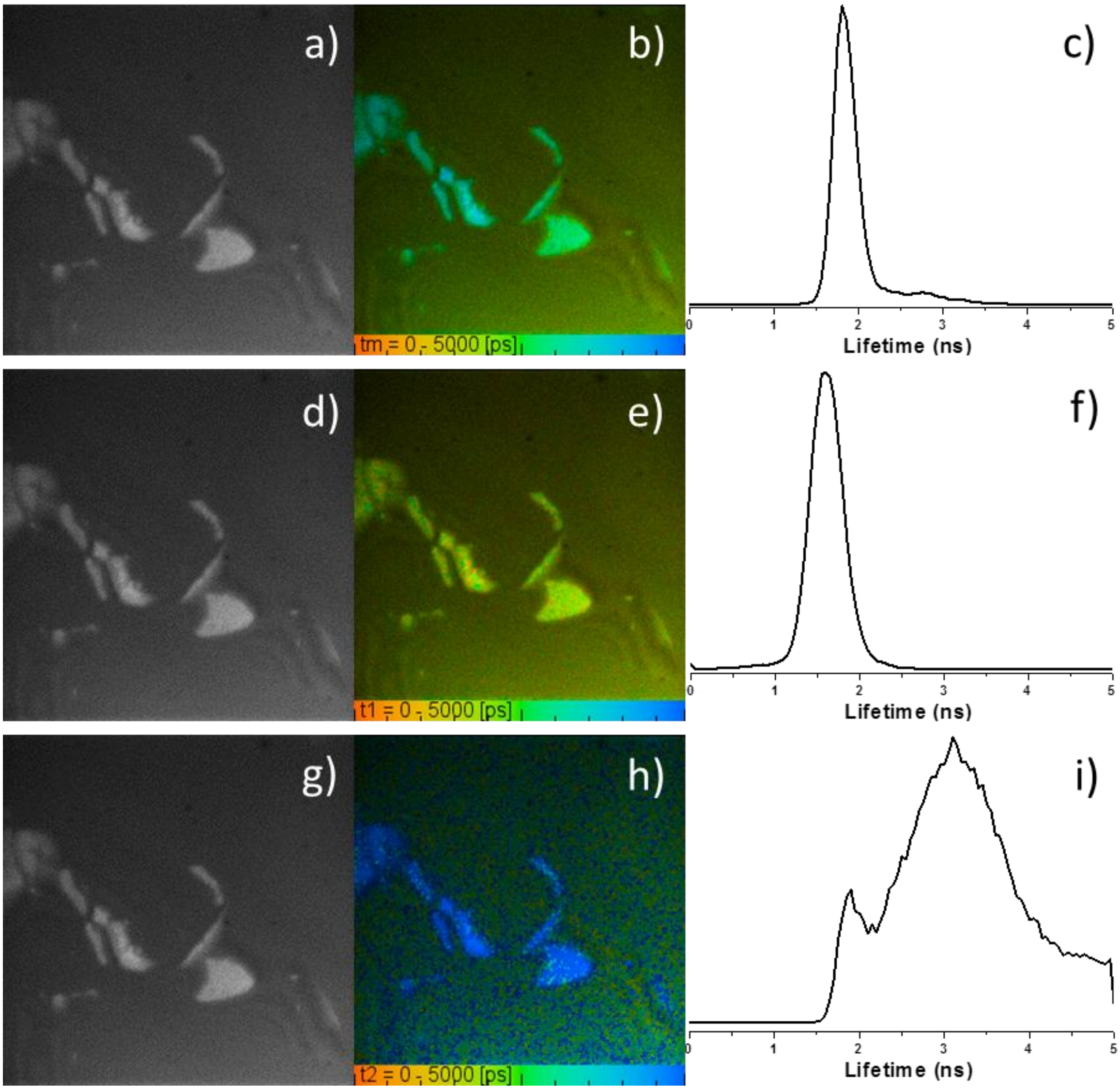

Figure S24. Single-photon laser FLIM of a thin film obtained by the drop casting of a 1 mg/mL solution of free base porphyrin **3** onto borosilicate glass, (a) intensity image, λ_{ex} = 405 nm; (b) lifetime mapping of τ_m, scale bar: 20 μm; (c) corresponding fluorescence lifetime τ_m distribution curve; (d) intensity image, λ_{ex} = 405 nm; (e) lifetime mapping of τ_1, scale bar: 20 μm; (f) corresponding fluorescence lifetime τ_2 distribution; (g) intensity image, λ_{ex} = 405 nm; (h) lifetime mapping of τ_2, scale bar: 20 μm (i) corresponding fluorescence lifetime τ_2 distribution curve.

2P TCSPC	χ^2	t_1 (ps)	t_1 (%)
**1**@rGO dispersion	1.07	459	100

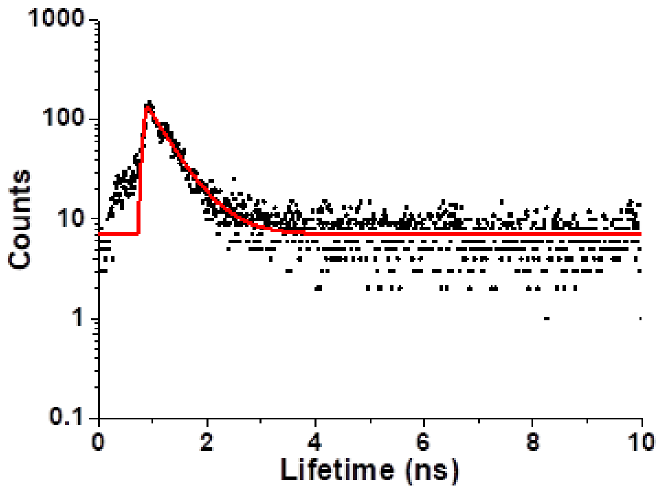

Figure S25. Dispersed phase 2–photon TCSPC spectrum ($CHCl_3$:EtOH, 1 mg/mL) of **1**@rGO composite, λ_{ex} = 810 nm, laser power: 5.6 mW.

2P TCSPC	χ^2	t_1 (ps)	t_1 (%)
**2**@rGO dispersion	1.43	406	100

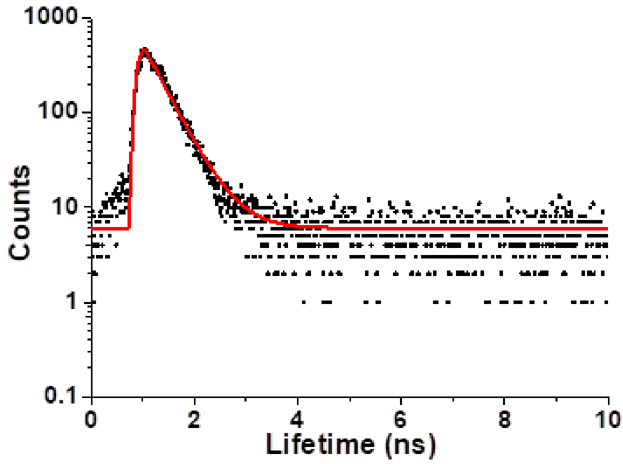

Figure S26. Dispersed phase 2–photon TCSPC spectrum ($CHCl_3$:EtOH, 1 mg/mL) of a **2**@rGO composite, λ_{ex} = 810 nm, laser power: 5.0 mW.

2P TCSPC	χ^2	t_1 (ps)	t_1 (%)
**3**@rGO dispersion	1.10	402	100

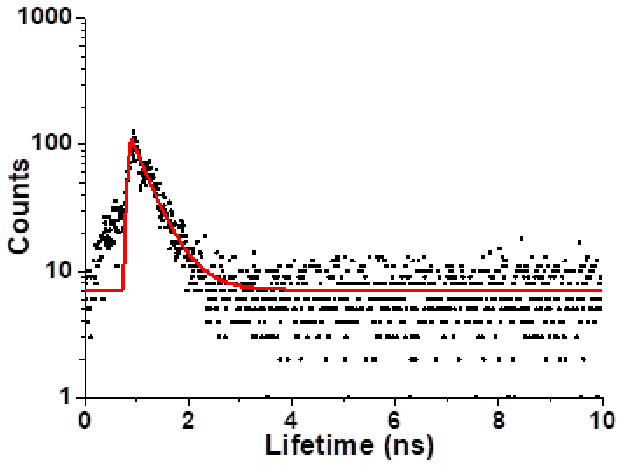

Figure S27. Dispersed phase TCSPC spectrum ($CHCl_3$:EtOH, 1 mg/mL) of **3**@rGO composite, λ_{ex} = 810 nm, laser power: 5.6 mW.

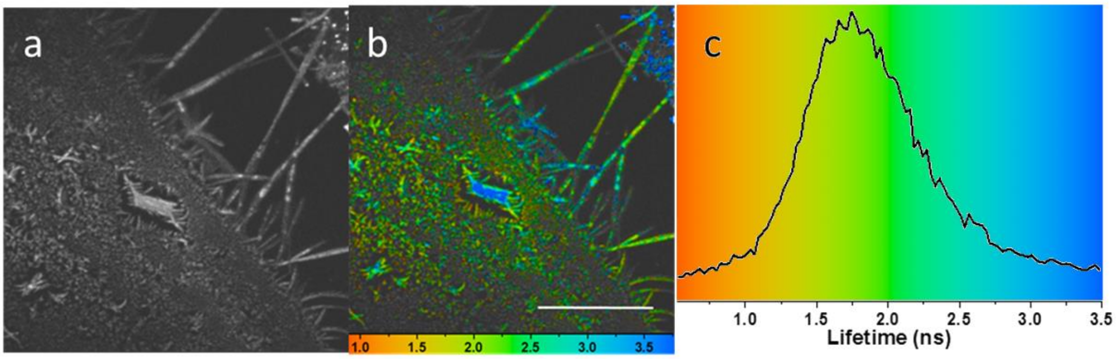

Figure S28. Two-photon fluorescence lifetime imaging microscopy of a thin film of drop-casted **2**@coronene composite, λ_{ex} = 810 nm, laser power was 0.1mW: **(a)** intensity image; **(b)** average fluorescence lifetime mapping and scale bar: 50 μm; **(c)** corresponding average fluorescence lifetime distribution curve.

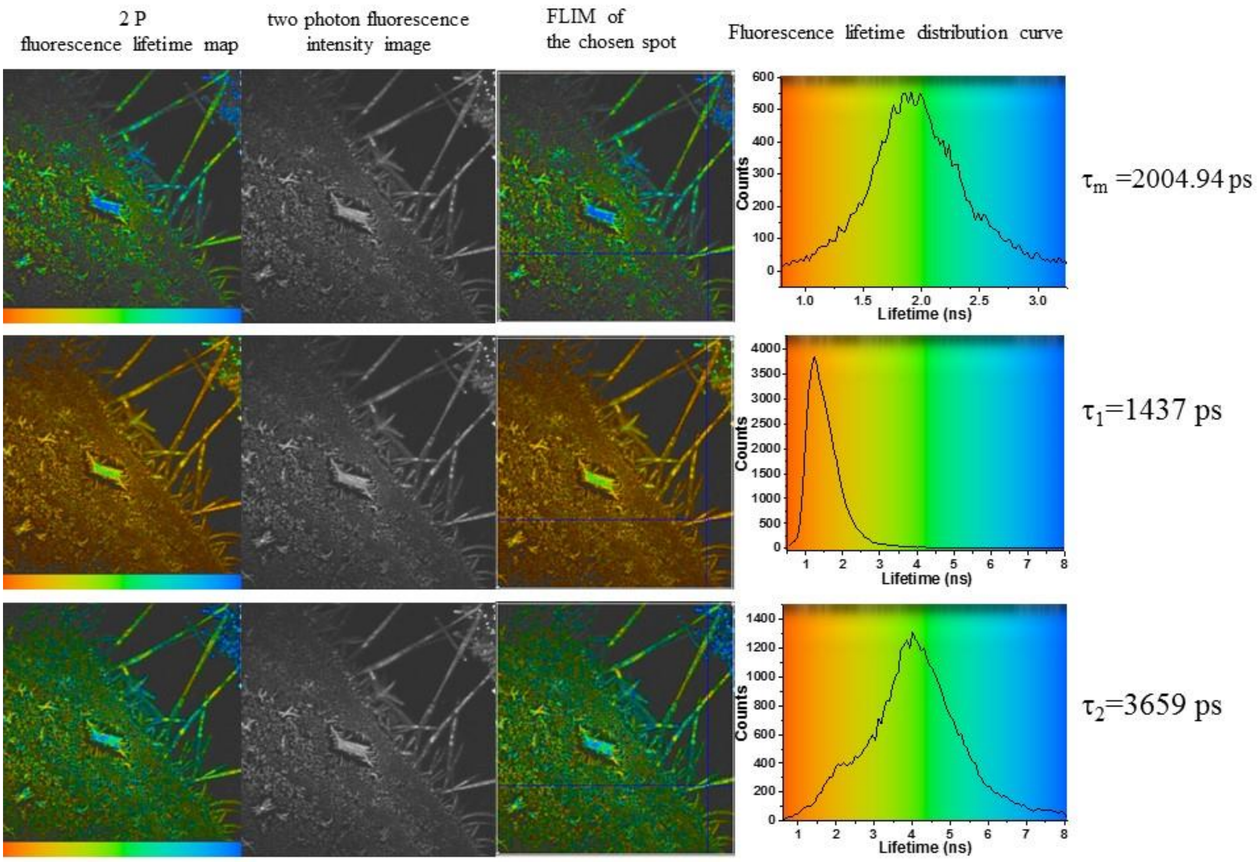

Chosen spot	χ^2	t_1 (ps)	t_1 (%)	t_2 (ps)	t_2 (%)	t_m
Compound 2@coronene	1.08	1437	74.4	3659	25.6	2004.94

Figure S29. Two-photon fluorescence lifetime imaging microscopy of a thin film of drop-casted **2**@coronene composite showing the two-component lifetime present in thin film.

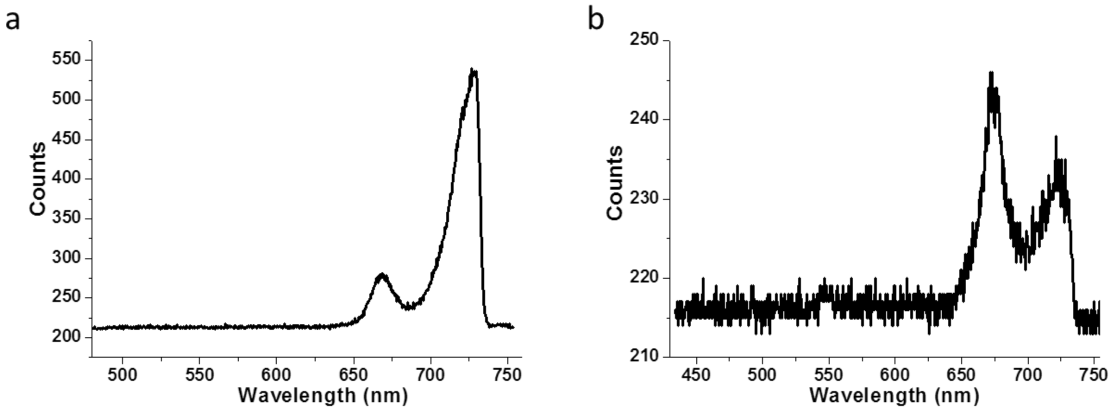

Figure S30. Two photon fluorescence emission spectrum of: (a) **2**@coronene composite dispersed in $CHCl_3$:EtOH (1 mg/ml), laser power: 3.0 mW; (b) **2**@ coronene in thin film, laser power: 1.0 mW. λ_{ex} = 810 nm.

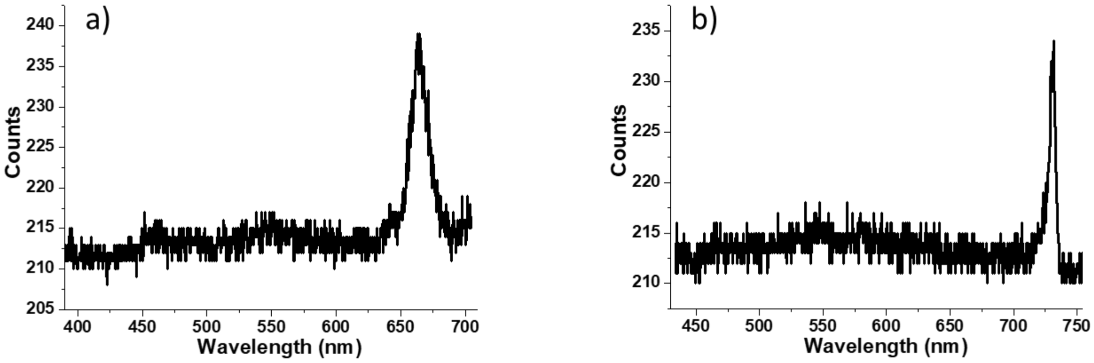

Figure S31. Two photon fluorescence spectrum of: (a) **2**@rGO, laser power was 5.0 mW; (b) **1**@ coronene in solution, laser power: 5.0 mW, λ_{ex} = 810 nm.

2P TCSPC	χ^2	t_1 (ps)	t_1 (%)	t_2 (ps)	t_2 (%)	t_m
**2**@coronene composite	1.06	318	51	6806.3	49	3495.7

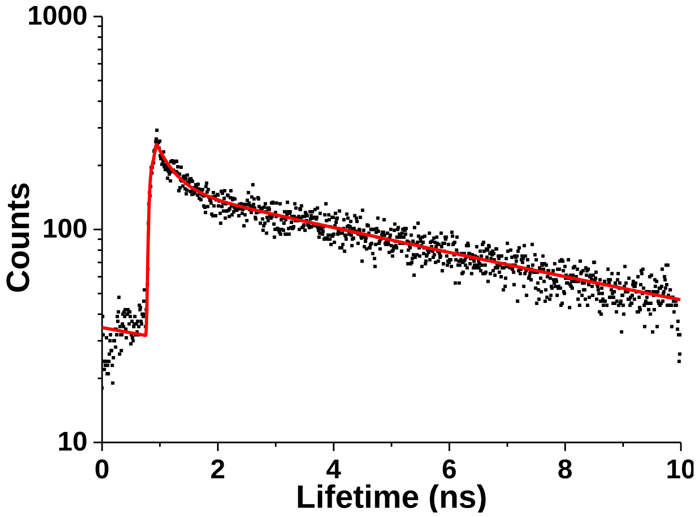

Figure S32. Solution 2P TCSPC spectrum ($CHCl_3$, 1 mg/mL) of the **2**@coronene composite, λ_{ex} = 810 nm, laser power was 5.0 mW.

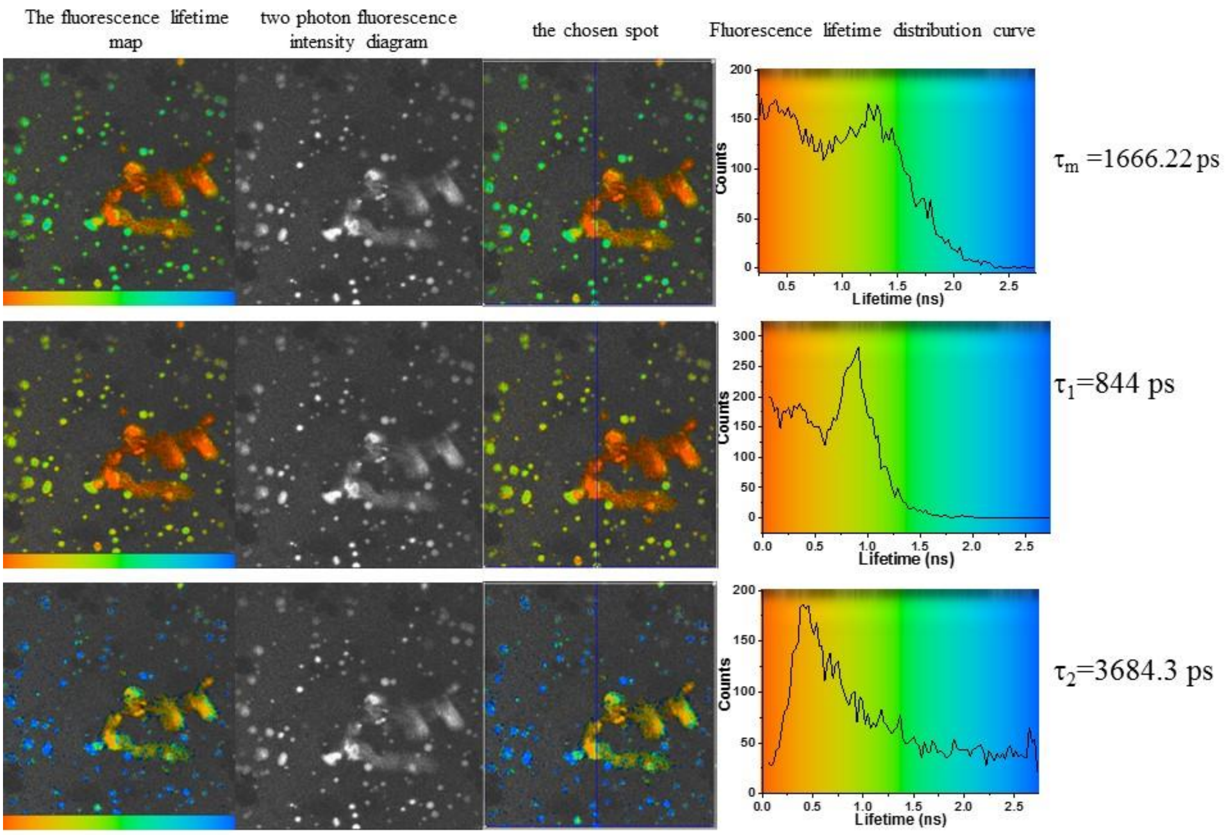

Chosen spot	χ^2	t_1 (ps)	t_1 (%)	t_2 (ps)	t_2 (%)	t_m
Compound 3@rGO	1.10	844	71.1	3684.3	28.9	1666.22

Figure S33. Two-photon photon confocal microscopy of **3**@rGO in thin film (810 nm, 0.5mW).

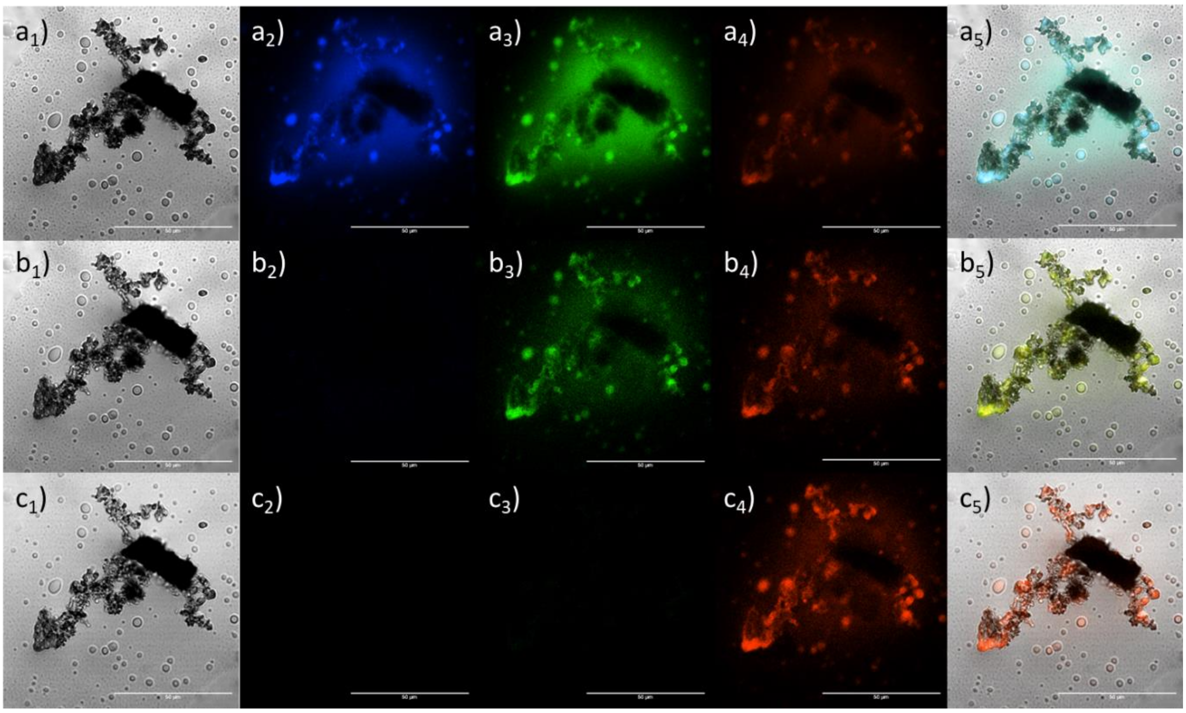

Figure S34. Single photon confocal microscopy of **3**@rGO in thin film. a_1-a_5) λ_{ex} 405 nm; b_1-b_5) λ_{ex} 488 nm; c_1-c_5) λ_{ex} 543 nm; a_1), b_1) c_1) DIC channel; a_2), b_2) c_2) blue channel; a_3), b_3) c_3) green channel; a_4), b_4) c_4) red channel; a_5), b_5) c_5) overlay of channels 1-4.

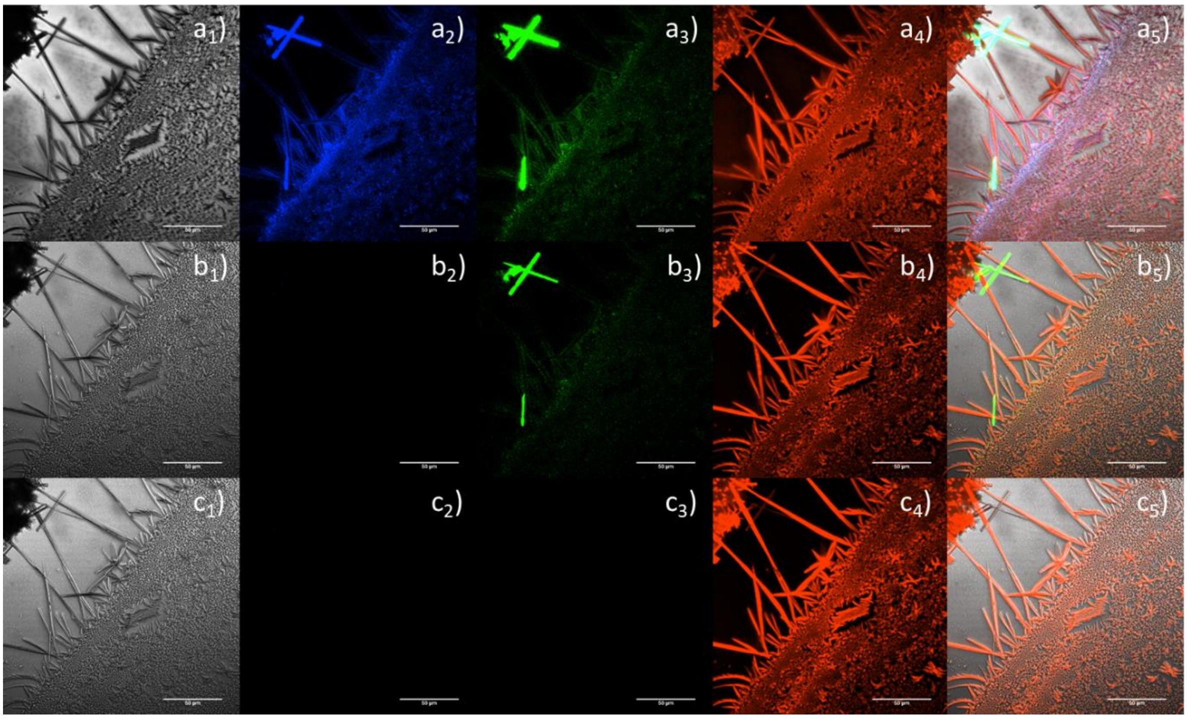

Figure S35. Single photon confocal microscopy of **2**@coronene in thin film. a_1-a_5) λ_{ex} 405 nm; b_1-b_5) λ_{ex} 488 nm; c_1-c_5) λ_{ex} 543 nm; a_1), b_1) c_1) DIC channel; a_2), b_2) c_2) blue channel; a_3), b_3) c_3) green channel; a_4), b_4) c_4) red channel; a_5), b_5) c_5) overlay of channels 1-4.

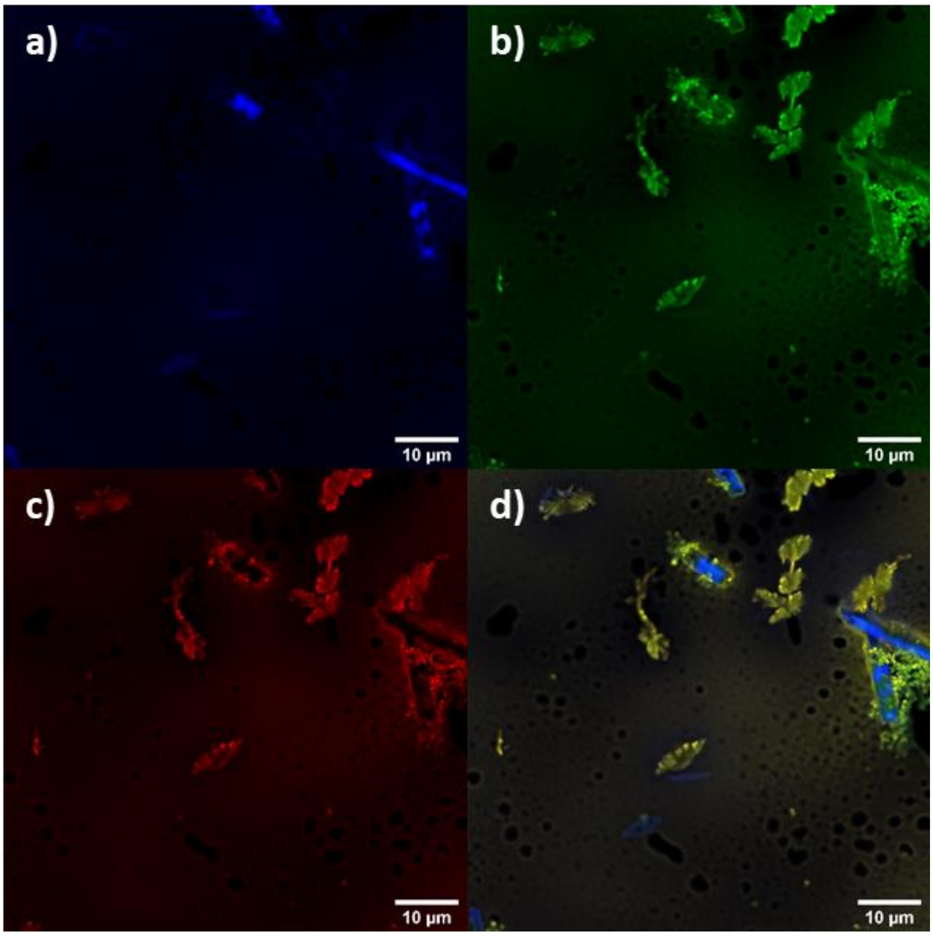

Figure S36. Super-resolution Airyscan confocal microscopy images of **1**@coronene hybrids. a) Blue channel (λ_{exc} 405 nm); b) green channel (λ_{exc} 488 nm); c) red channel (λ_{exc} 561 nm); d) overlay of blue, green and red channels.

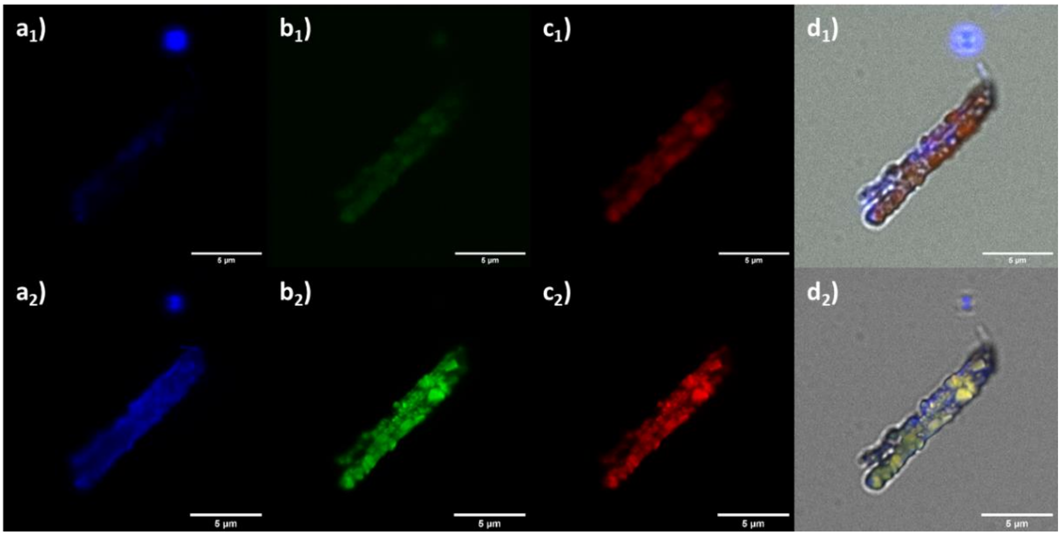

Figure S37. Fluorescence confocal microscopy images (a_1-d_1) and super resolution Airyscan confocal microscopy images (a_2-d_2) of a **2**@coronene crystal. a_{1-2}) Blue channel (λ_{exc} 405 nm); b_{1-2}) green channel (λ_{exc} 488 nm); c_{1-2}) red channel (λ_{exc} 561 nm); d_{1-2}) overlay of brightfield, blue, green and red channels.

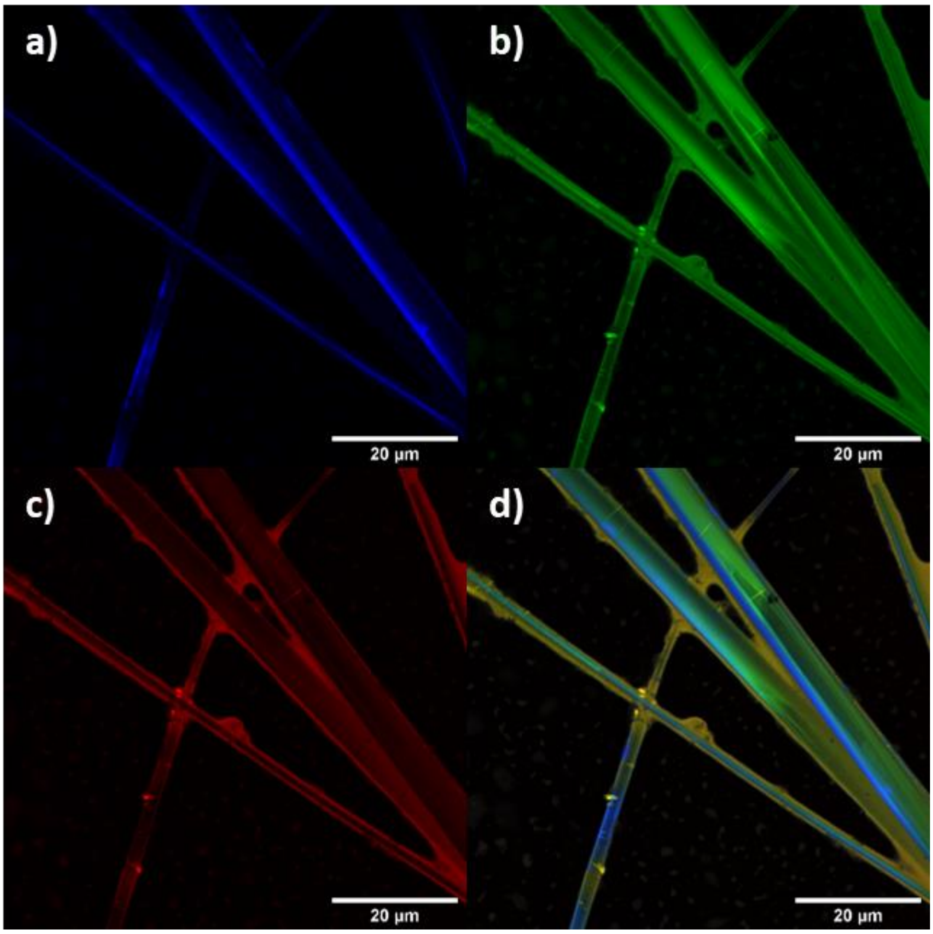

Figure S38. Super-resolution Airyscan confocal microscopy images of **3**@coronene hybrid. a) Blue channel (λ_{exc} 405 nm); b) green channel (λ_{exc} 488 nm); c) red channel (λ_{exc} 561 nm); d) overlay of blue, green and red channels.

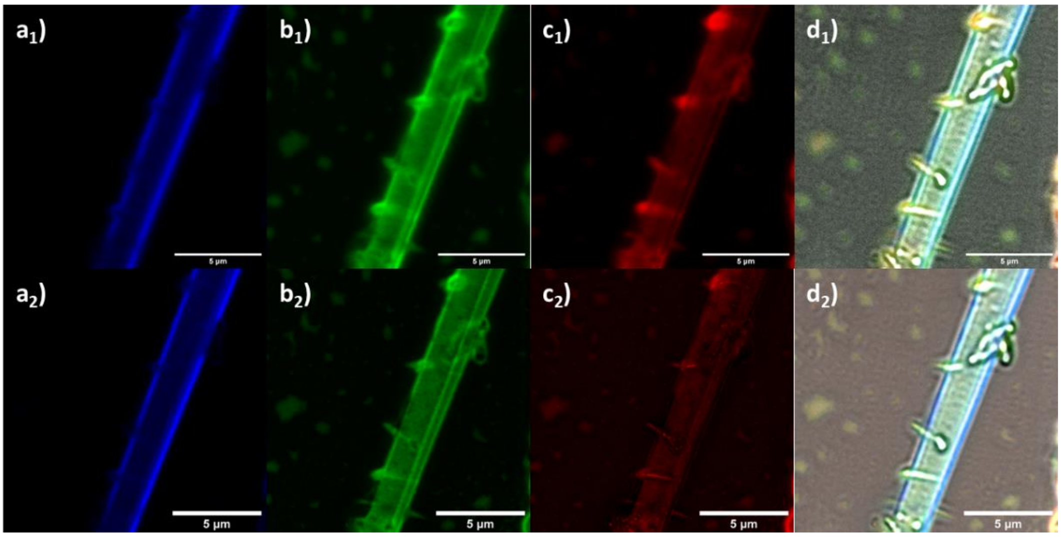

Figure S39. Fluorescence confocal microscopy images (a_1-d_1) and super resolution Airyscan confocal microscopy images (a_2-d_2) of a crystal of **3**@coronene. $a_{1\text{-}2}$) Blue channel (λ_{exc} 405 nm); $b_{1\text{-}2}$) green channel (λ_{exc} 488 nm); $c_{1\text{-}2}$) red channel (λ_{exc} 561 nm); $d_{1\text{-}2}$) overlay of brightfield, blue, green and red channels.

10. AFM microscopy on mica and on HOPG supports

AFM images of **1**, **2**, **3** and coronene hybrids were acquired in freshly cleaved mica or HOPG substrates (Agar scientific) and deposited by immersion in the corresponding 0.5 mM $CHCl_3$/toluene (1:1) solutions for 1.5 h at room temperature and dried under a nitrogen flow.[18] This followed a previously described procedure for the controlled stacking of aromatic molecules onto HOPG by surface assembly[18] and the solvent mixture was maintained from titration experiments for consistency. Images were acquired with silicon tips with resonant frequency at 70 kHz (Scout 70, Nunano).

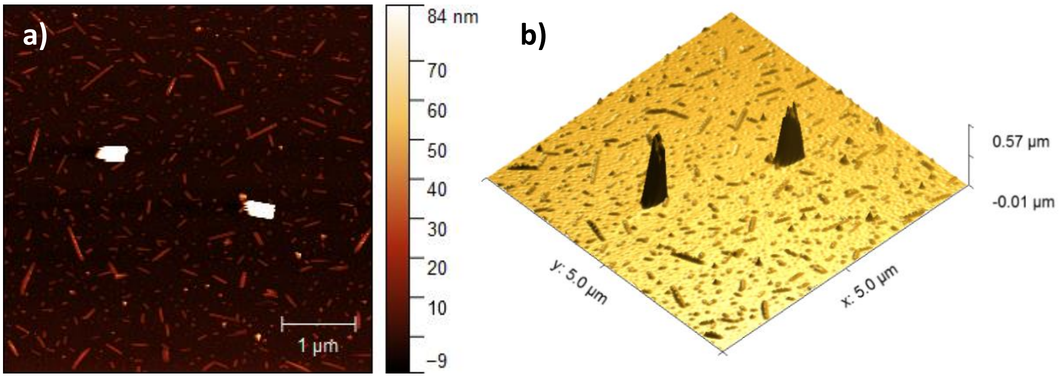

Figure S40. Tapping mode AFM image of coronene on a mica substrate; b) 3D tapping mode image of a).

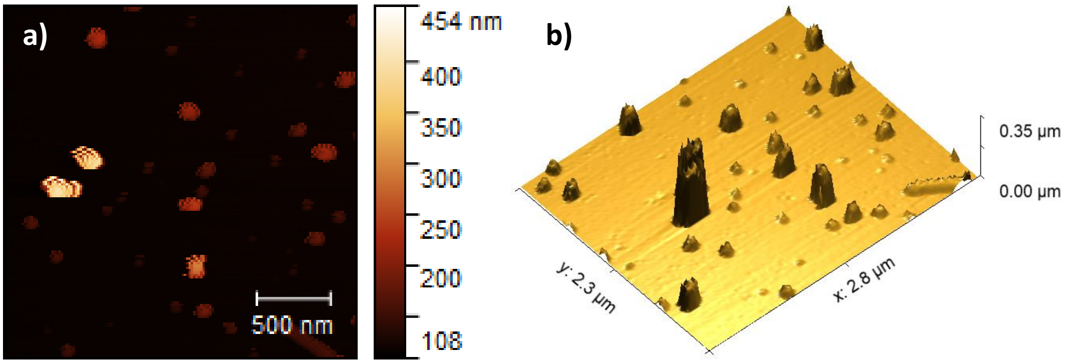

Figure S41. Tapping mode AFM image of coronene on a HOPG substrate; b) 3D tapping mode image of a).

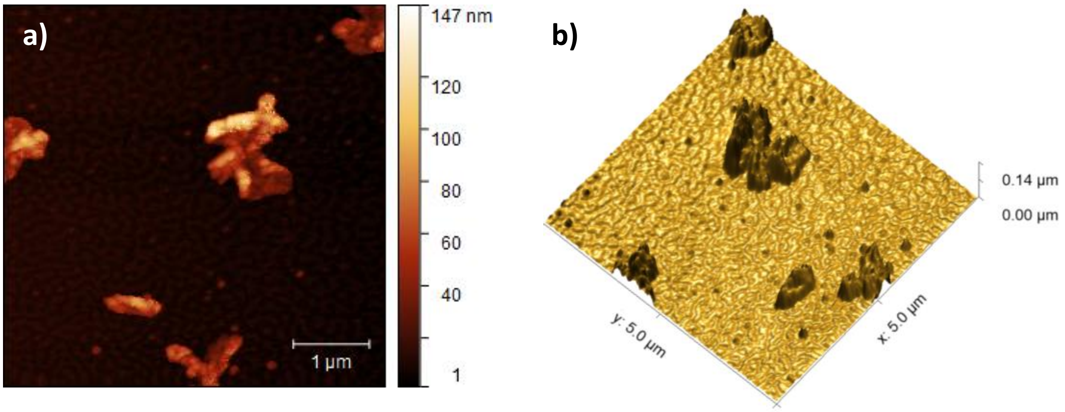

Figure S42. Tapping mode AFM image of **1** on a mica substrate; b) 3D tapping mode image of a).

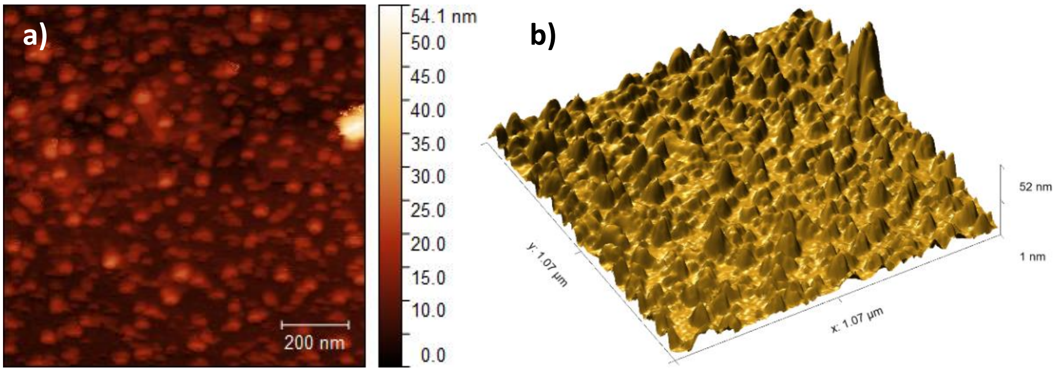

Figure S43. Tapping mode AFM image of **1** on a HOPG substrate; b) 3D tapping mode image of a).

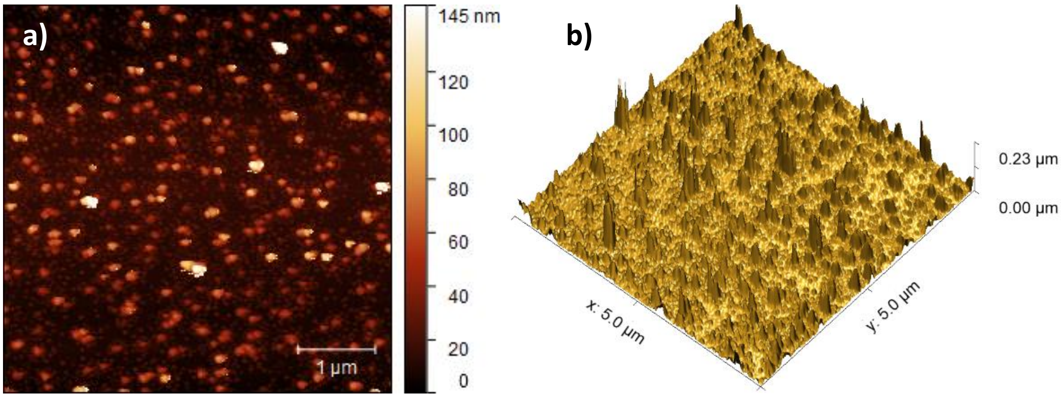

Figure S44. Tapping mode AFM image of **2** on a mica substrate; b) 3D tapping mode image of a).

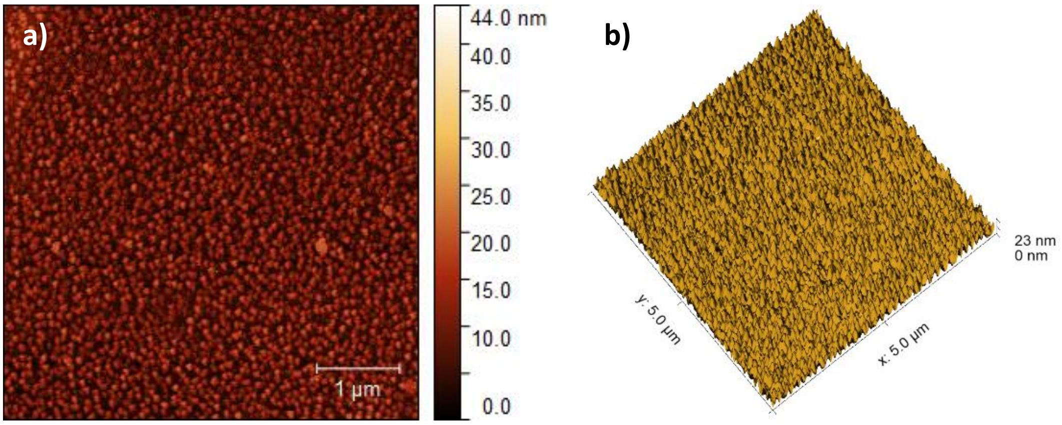

Figure S45. Tapping mode AFM image of **2** on a HOPG substrate; b) 3D tapping mode image of a).

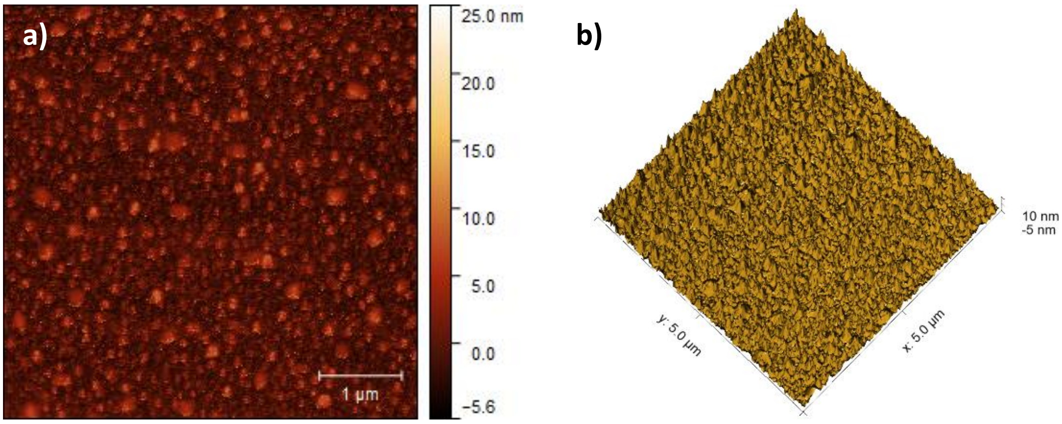

Figure S46. Tapping mode AFM image of **3** on a mica substrate; b) 3D tapping mode image of a).

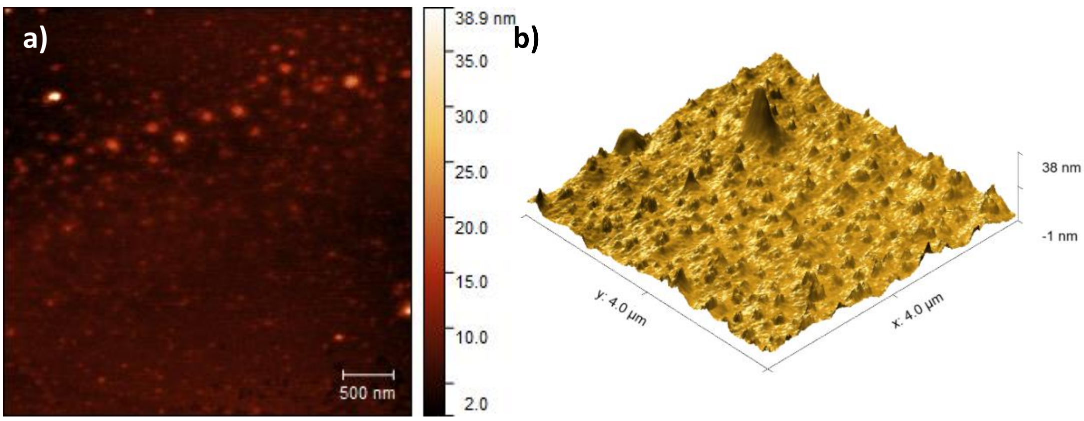

Figure S47. Tapping mode AFM image of **3** on a HOPG substrate; b) 3D tapping mode image of a).

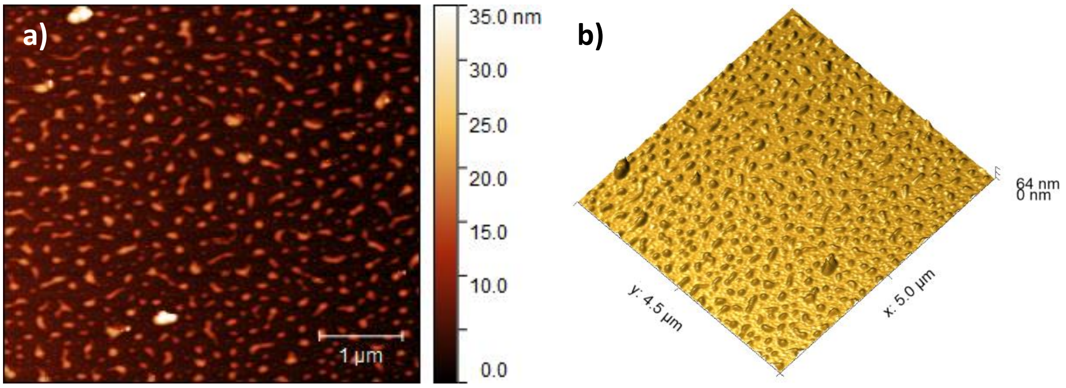

Figure S48. Tapping mode AFM image of **1**@coronene on a mica substrate; b) 3D tapping mode image of a).

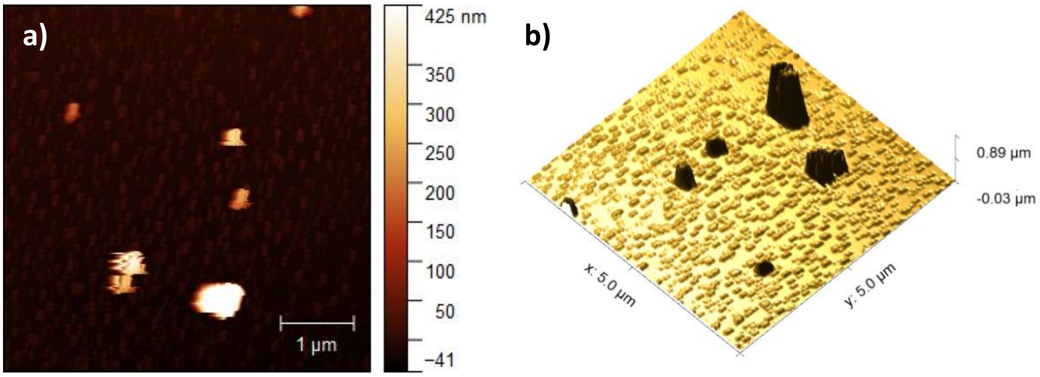

Figure S49. Tapping mode AFM image of **1**@coronene on a HOPG substrate; b) 3D tapping mode image of a).

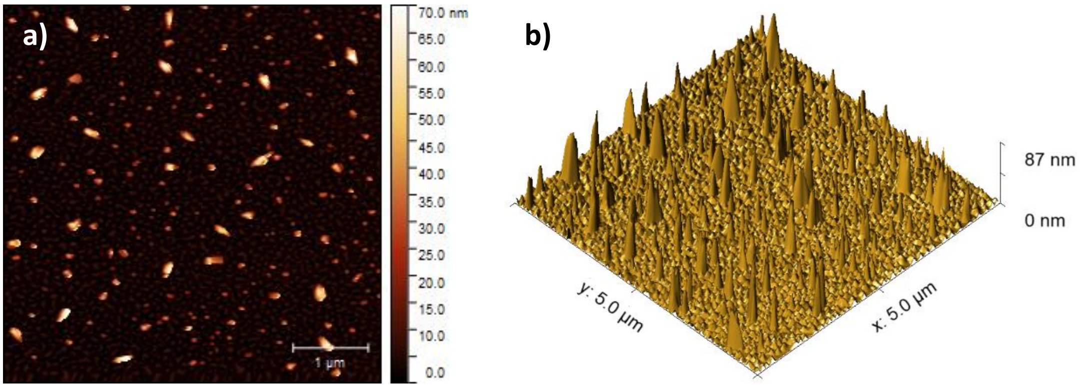

Figure S50. Tapping mode AFM image of **2**@coronene on a mica substrate; b) 3D tapping mode image of a).

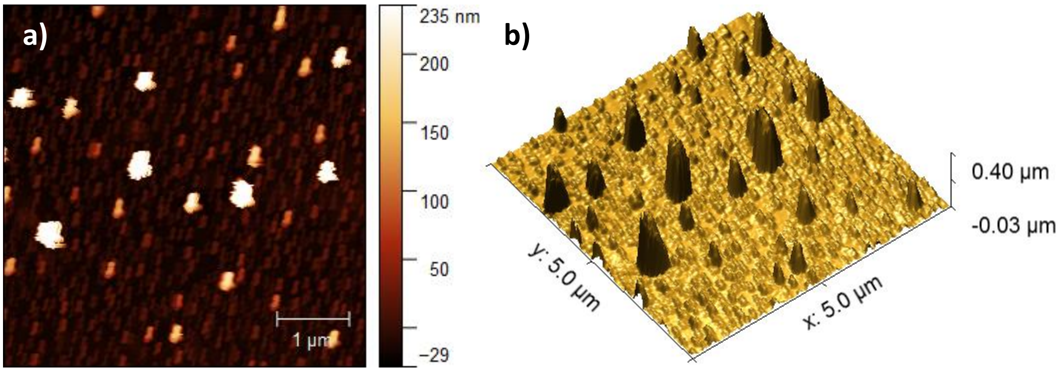

Figure S51. Tapping mode AFM image of **2**@coronene on a HOPG substrate; b) 3D tapping mode image of a).

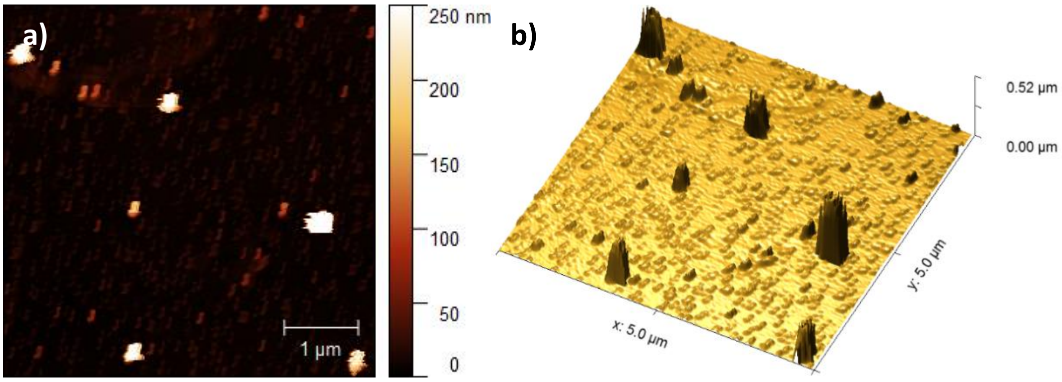

Figure S52. Tapping mode AFM image of **3**@coronene on a mica substrate; b) 3D tapping mode image of a).

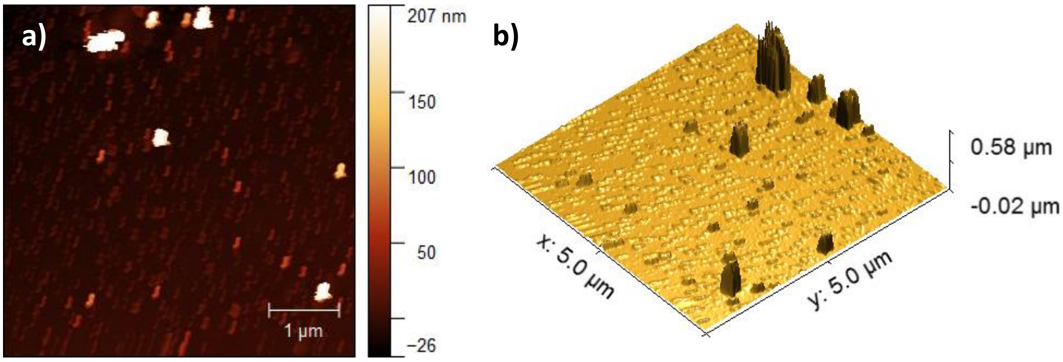

Figure S53. Tapping mode AFM image of **3**@coronene on a HOPG substrate; b) 3D tapping mode image of a).

11. Scanning electron microscopy

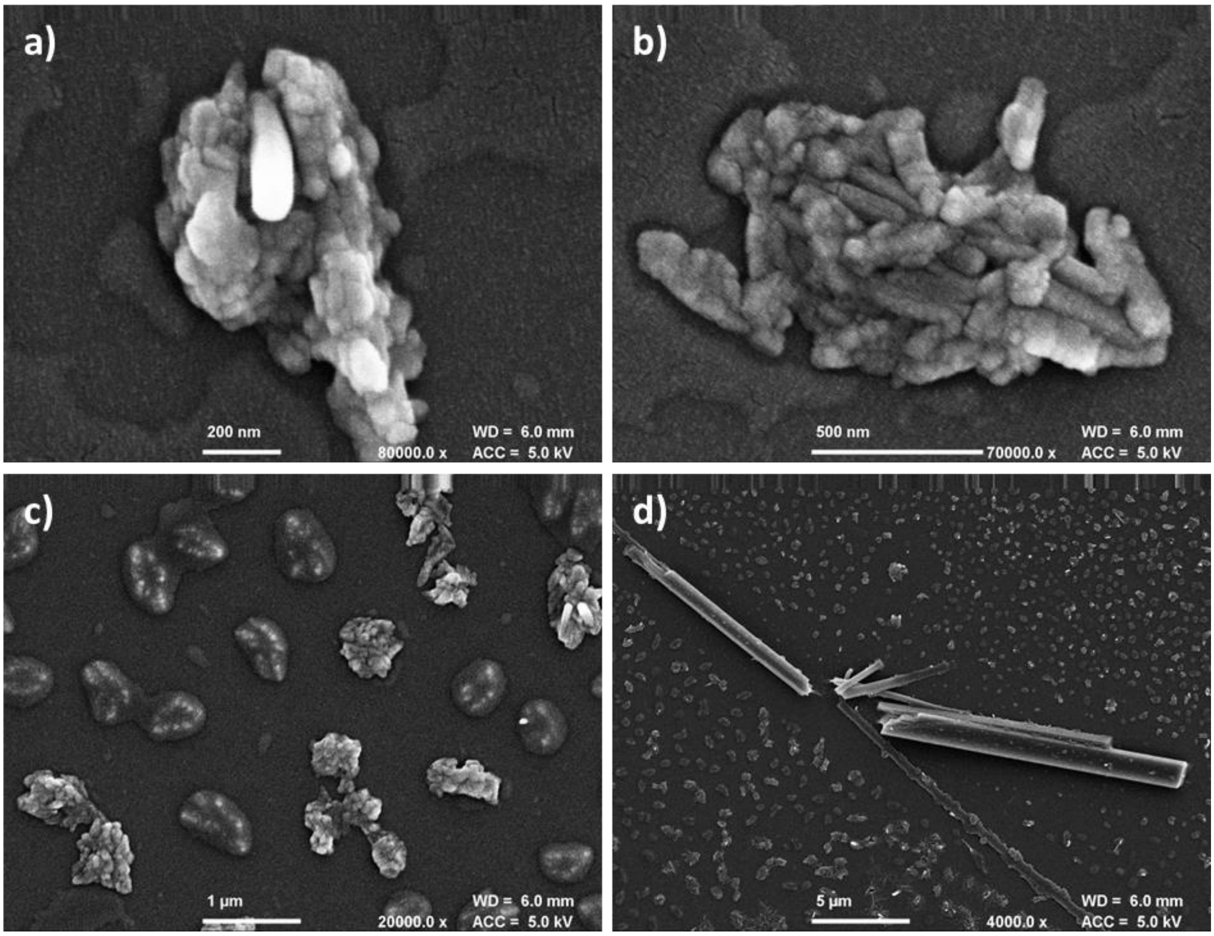

Figure S54. FE-SEM images of **1**@coronene hybrid at different magnifications. Scale bars: a) 200 nm; b) 500 nm; c) 1 µm; d) 5 µm.

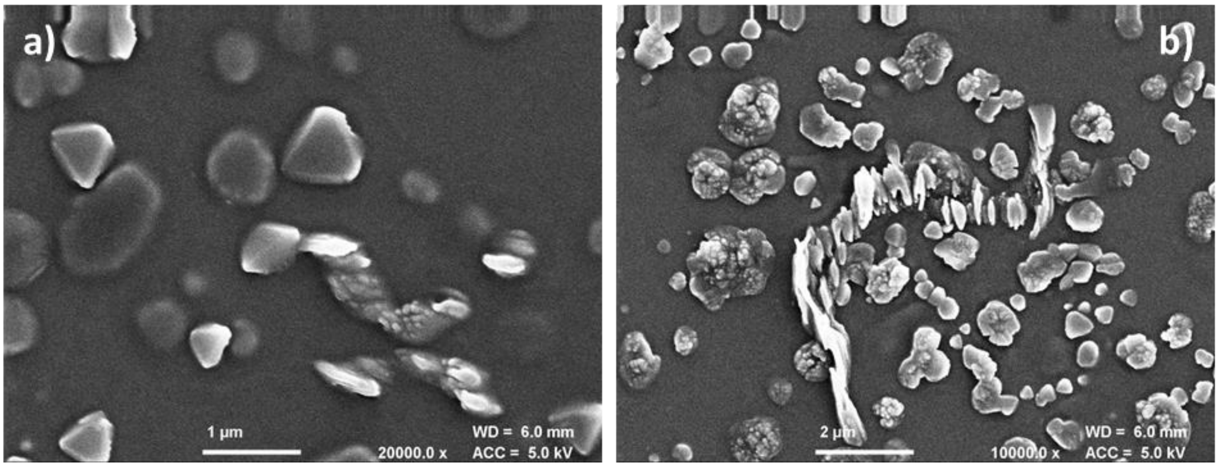

Figure S55. FE-SEM images of **2**@coronene hybrid at different magnifications. Scale bars: a) 1 µm; b) 2 µm.

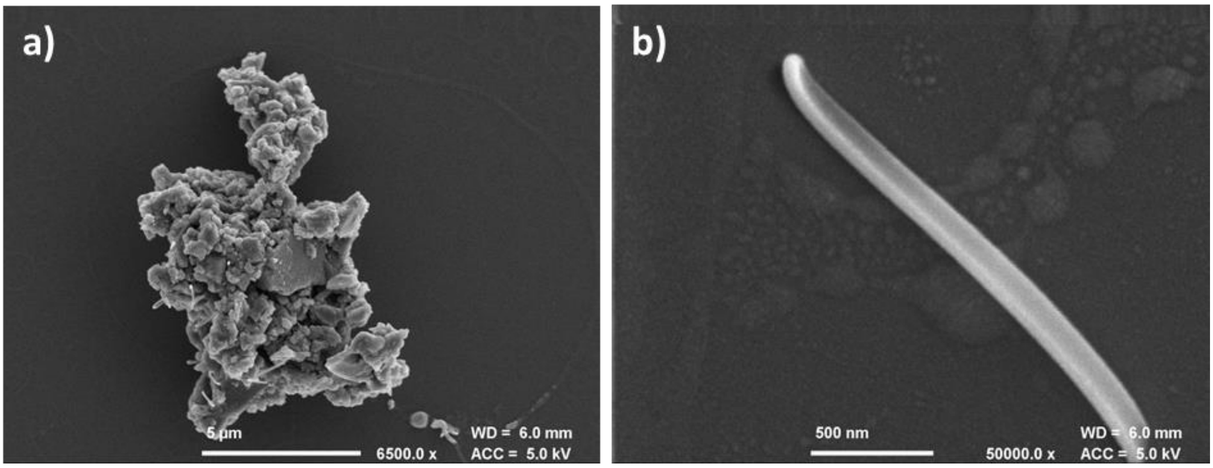

Figure S56. FE-SEM images of **3**@coronene hybrid at different magnifications. Scale bars: a) 5 µm; b) 500 nm.

The SEM measurements of porphyrin and rGO composite were carried out on samples generated by the freeze drying of composite samples to remove the solvents, and then transferring the solid samples onto a HOPG substrate. The samples ready for microscopy were further freeze dried to avoid any structured changes during the measurement. The SEM images suggested morphologies consistent onto a 3-dimensional structure of the synthesised composites in solid state. The hydrothermal reduced graphene oxide sheets formed a micro porous network structure, whereby the hole sizes obtained in compound **1**@rGO composite ranged from 2 µm to 5 µm, while the hole size of compound **2**@rGO composite showed a sligh difference: the range were from 0.5 µm to 5 µm. The hole size of the compound **3**@rGO composite showed a dramatic decrease compared with the other two composites, the surface morphology of the compound **3**@rGO became more dense, with reduce smaller-sized network.

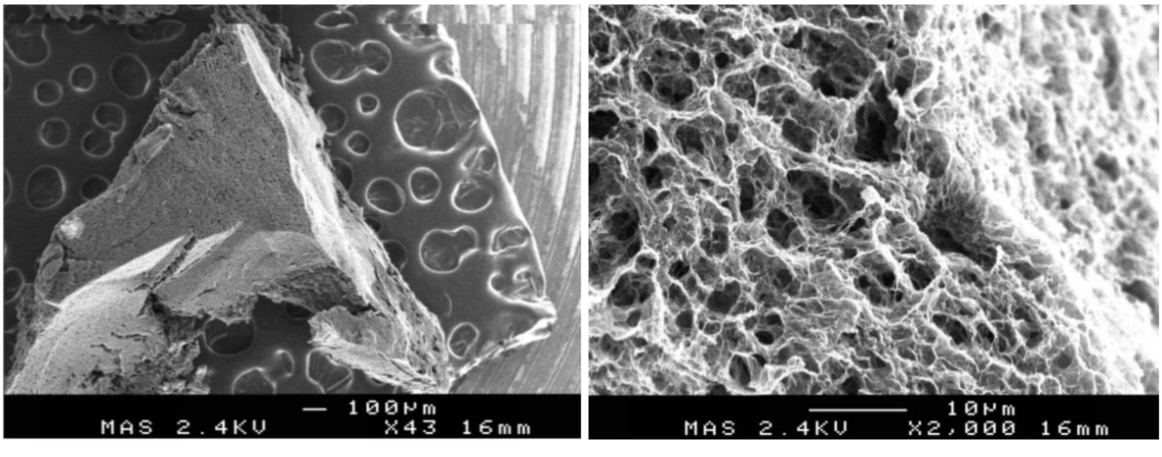

Figure S57. SEM images of compound **1**@rGO homo-composite at different magnifications, the scale bar is 100 µm (left) and 10 µm (right) respectively.

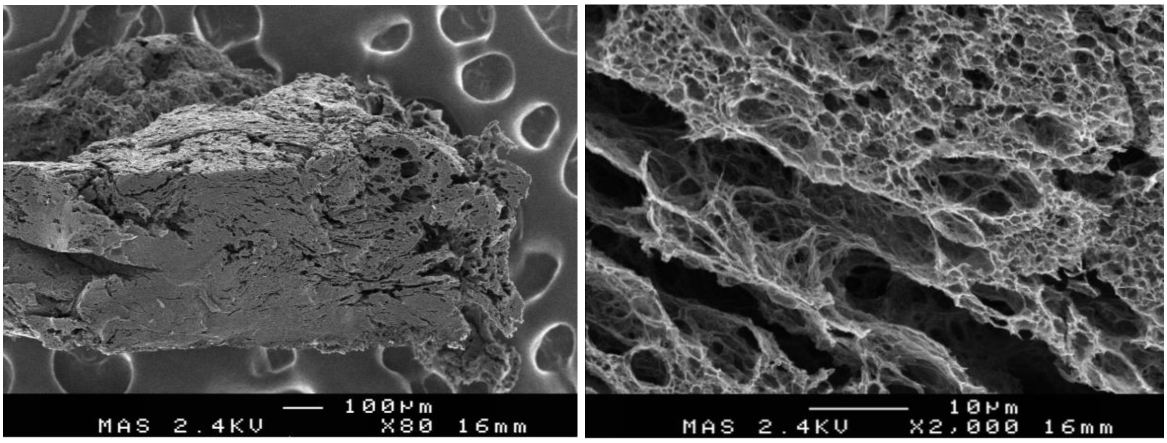

Figure S58. SEM images of compound **2**@rGO homo-composite at different magnifications, the scale bar is 100 μm (left) and 10 μm (right) respectively.

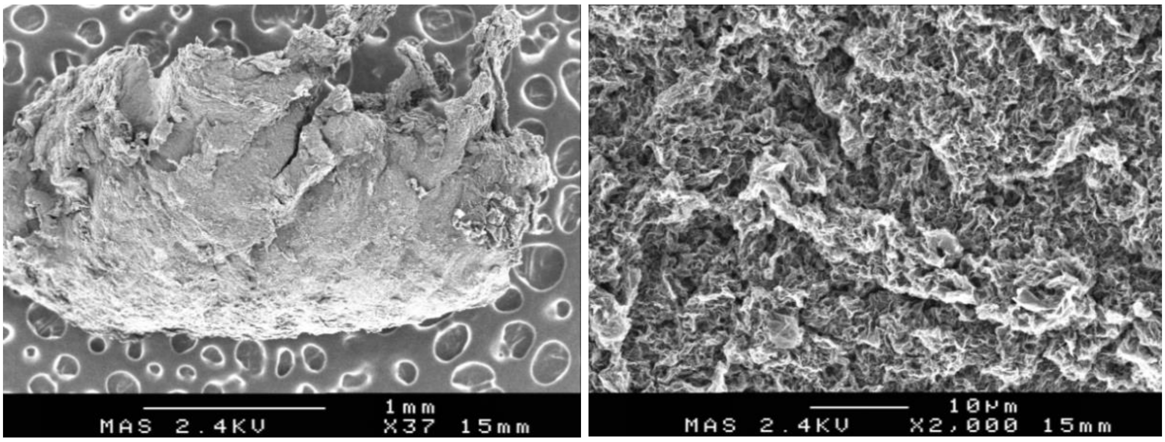

Figure S59. SEM images of compound **3**@rGO homo-composite at different magnifications; the scale bar is 1 mm (left) and 10 μm (right) respectively.

.

12. Transmission Electron microscopy experiments

The TEM measurements of porphyrin containing rGO composites were carried out on samples resulting by drop-casting the composite dispersion onto lacy carbon TEM grids. Figure S60 shows the TEM images of the graphene oxide starting material. Figure S60 (b) is the selected area electron diffraction (SAED), from the diffraction pattern, it can be seen there were 6 sharp and intense diffraction points appeared in the first friction order, which indicated a crystalline structure. Apart from the 6 sharp diffraction points, several relatively light points were combined to afford a circle diffraction ring, it means that the graphene oxide was not a single layer structure, instead, the graphene oxide were a few layer sheets overlaid, those

sheets are rotation oriented with each other and the properties of each layer are unique. The interlayer coherence is not destroyed.

The HRTEM were recorded at the edge of graphene oxide sheet, at which part the images could exhibit more details without the disruption of overlaying. From Figure S60 (c), it can be found from the edge of the graphene oxide sample, the sample was consisted three layers. Form the images, some defects and disorder on the graphene oxide surface can also be observed. Figure S60 (d), the sample were consisted with two layers, the lattice structures shown in the image were more clear and on defects or disorder appeared. The TEM coupled with SAED and HRTEM exhibited the detailed surface morphology information and the inner structure of graphene oxide. The measurements results gave a direct evidence of the formation of graphene oxide nanomaterials.

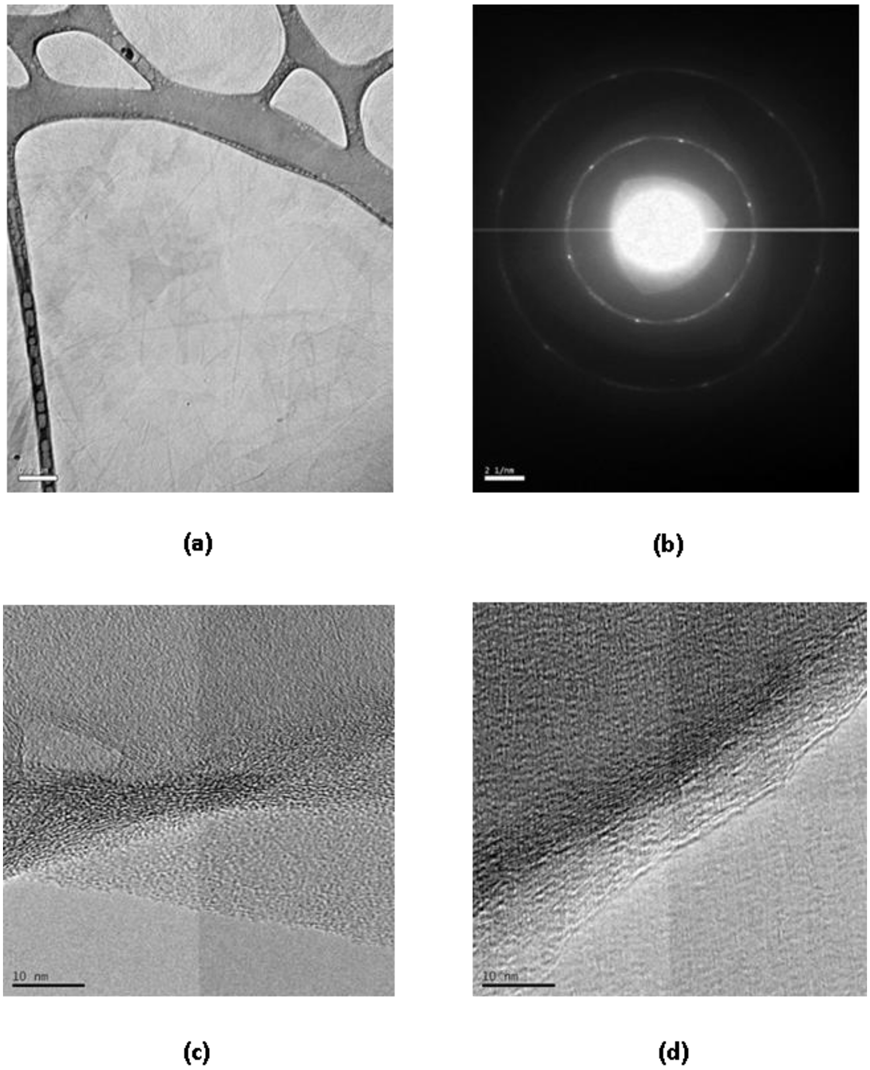

Figure S60. TEM images of graphene oxide starting material, scale bar =200 nm, **(b)** SAED of graphene oxide, the scale bar =2 1/nm, **(c)** HRTEM image of graphene oxide, scale bar = 10 nm and **(d)** HRTEM image of graphene oxide, scale bar = 10 nm.

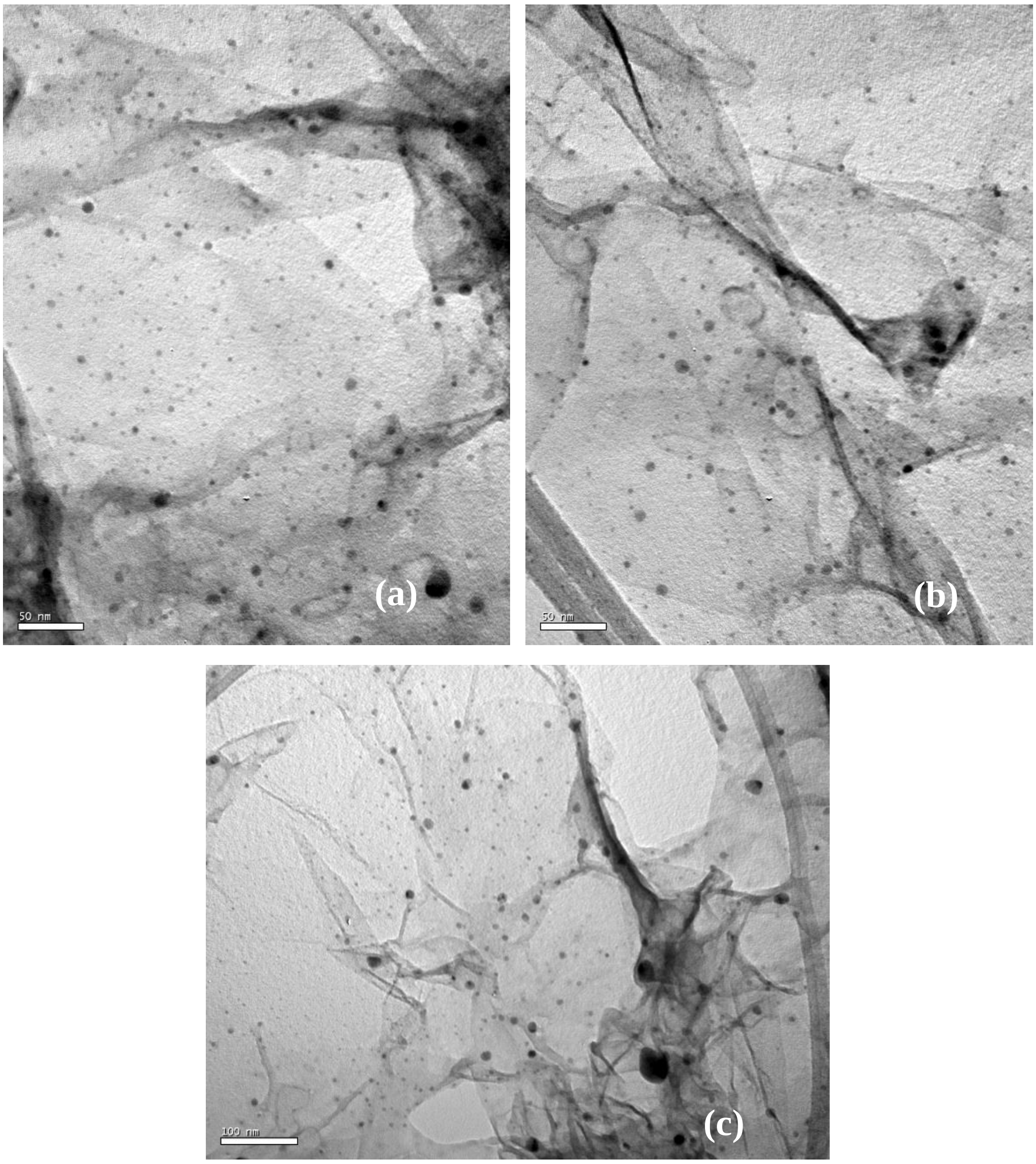

Figure S61. TEM images of **(a)** **1**@rGO composite, the scale bar=50nm, **(b)** compound **2**@rGO composite, the scale bar=50nm **(c)** compound **3**@rGO composite, the scale bar=100nm. TEM images show that the three different composite materials all show similar structures on the nanoscale, the porphyrins incorporated present as black dots with dimensions range from 1-5 nm onto the rGO surface.

13. DFT Calculations data:

Computational Details:

The solution of bulk material compounds **1-3** was studied by theoretical calculations at a semi-empirical level of the solvent accessible electrostatics potential surface. Calculations were carried out with the Avogadro software.[19] For compounds **2** and **3** this simulation was based on the reported X-ray data found for the molecular structure determination.[20] The software Marvin Space was used for data visualisation and manipulation.[21]

The molecular level DFT calculations are based on first-principles density functional theory together with dispersion correction (DFT+D). The VASP code[22, 23] was used to solve the standard Kohn-Sham equations using plane wave basis sets with the energy cut-off of 500 eV. A single k-point was used in all geometry optimisations. Densities of States (DOS) were plotted using 2×2×2 Monkhorst-Pack[24] k point mesh. For the exchange correlation energy term the Generalised Gradient Approximation (GGA) was used in a form of Perdew, Burke and Ernzerhof (PBE)[25]. Structure optimisations were performed using a conjugate gradient algorithm and the forces on the atoms were smaller than 0.001 eV/Å. A cubic supercell with a length of 35 Å was used in all calculations to make sure that adjacent images do not interact. We define the binding energy by the following equation.

$$E_{bind} = E_{PorPhy\_Coro} - E_{Porphy} - E_{Coro}$$

where E_{Porphy} is the total energy of the isolated porphyrin molecule, E_{Coro} is the total energy of the coronene and E_{PorPhy_Coro} is the total energy of the porphyrin binding the surface of a coronene molecule. Here we include van der Waals (vdW) forces by using pair-wise force field as implemented by Grimme *et al*.[26] in the VASP package. The optimisation of the structures was carried out using MMFF94 Force Field in Avogadro (v 1.2.0). The electrostatic surface potentials were calculated in MarvinSpace (v 16.9.5.0).

Table S8. Calculated distances at the binding of porphyrins to coronene.

	C-C (Å)	C-N (Å)	F-H (Å)
**1**@Coronene	3.919, 3.979, 4.067	4.014, 4.093, 4.098	-
**2**@Coronene	4.324, 4.475, 4.612	4.517, 4.575, 4.615	2.637, 2.649, 2.864
**3**@Coronene	3.891, 3.916, 4.202	4.439, 4.440, 4.512	

Table S9. Calculated binding energies of porphyrins-coronene interactions using dispersion (DFT+D) and without dispersion (DFT) and the estimated charge transfer using Bader approximation[27].

System	Total energy (eV)	
	**DFT**	**DFT+D**
Coronene	–262.17	–262.76
Porphyrin_**1**	–538.05	–539.60
Porphyrin_**2**	–540.67	–542.41
Porphyrin_**3**	–968.44	–971.66
Porphyrin_**1**@Coronene	–800.24	–803.22
Porphyrin_**2**@Coronene	–802.78	–805.69
Porphyrin_**3**@Coronene	–1230.51	–1235.28

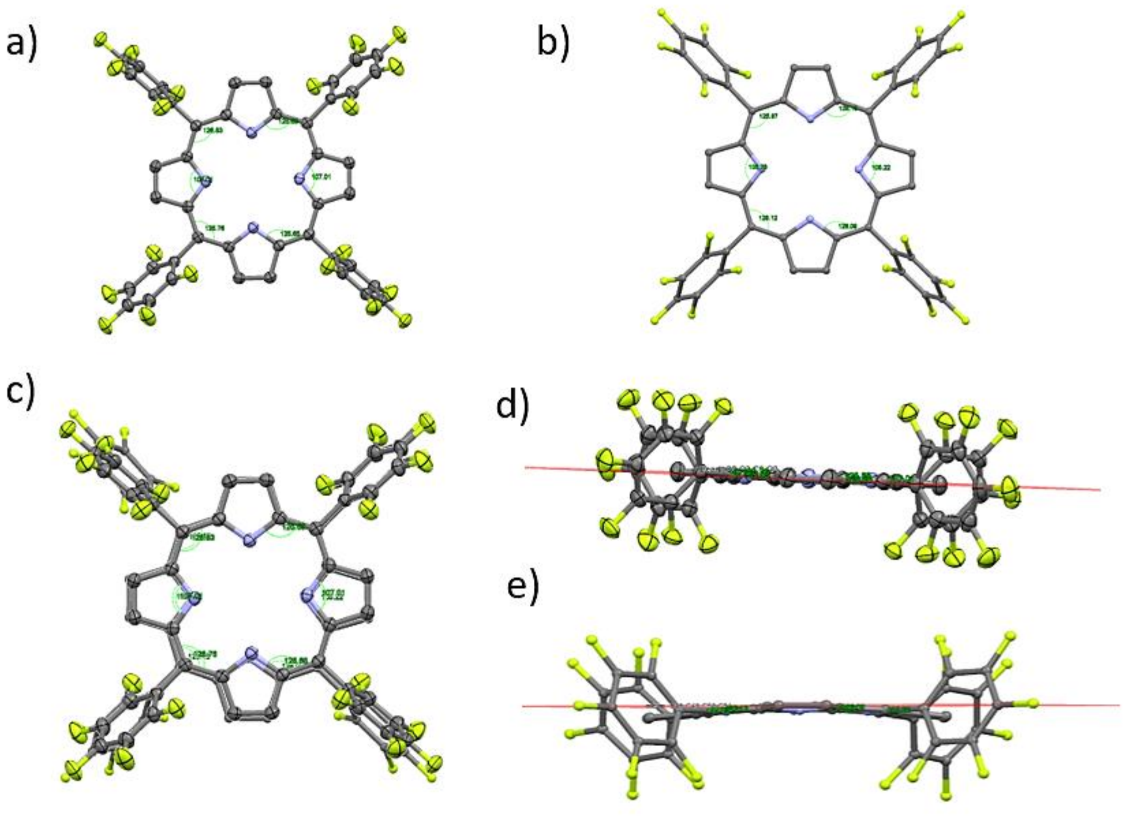

Figure S62. A comparison of the porphyrin component in **2**@coronene: (a) porphyrin model obtained from X-ray single crystal structure for porphyrin 2 (thermal ellipsoids at 50 % of probability), (b) theoretical model, (c) overlay of both structures, (d) X-ray single crystal structure for porphyrin 2 (thermal ellipsoids at 50 % of probability) and (e) theoretical model.

After a careful comparison of the X-ray single crystal structure of 2@coronene and a comparison and theoretical model for the geometry of porphyrin **2**, the main difference was that the porphyrin core is perfectly plane in the X-ray single crystal structure; however, the theoretical model presents a slight curvature (Figure S62, d) e)). The rest of structural parameters such bond lengths and angles are almost identical for the DFT calculated porphyrin 2 structure and the experimental data of **2@**coronene.

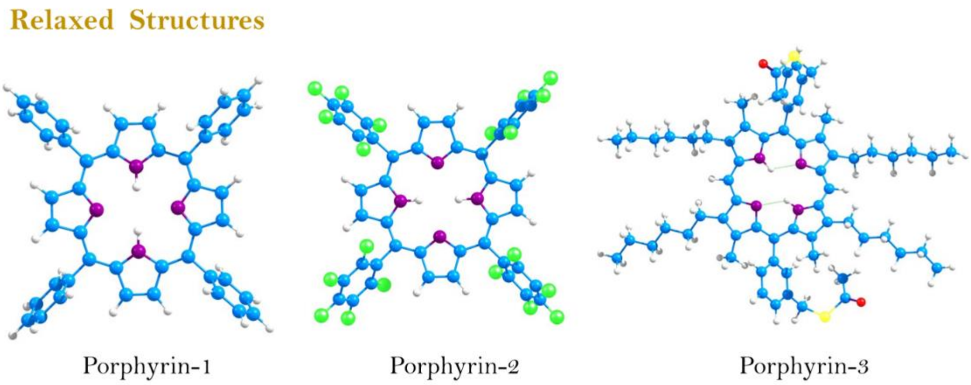

	Total energy (eV)	
	Dispersion	No-Dispersion
Porphyrin-1	−539.60	−538.05
Porphyrin-2	−542.41	−540.67
Porphyrin-3	−971.66	−968.44
Coronene	−262.76	−262.17
Porphyrin-1@Coronene	−803.22	−800.24
Porphyrin-2@Coronene	−805.69	−802.78
Porphyrin-3@Coronene	−1235.28	−1230.51

Figure S63. Geometry optimisations by DFT of porphyrins **1-3**.

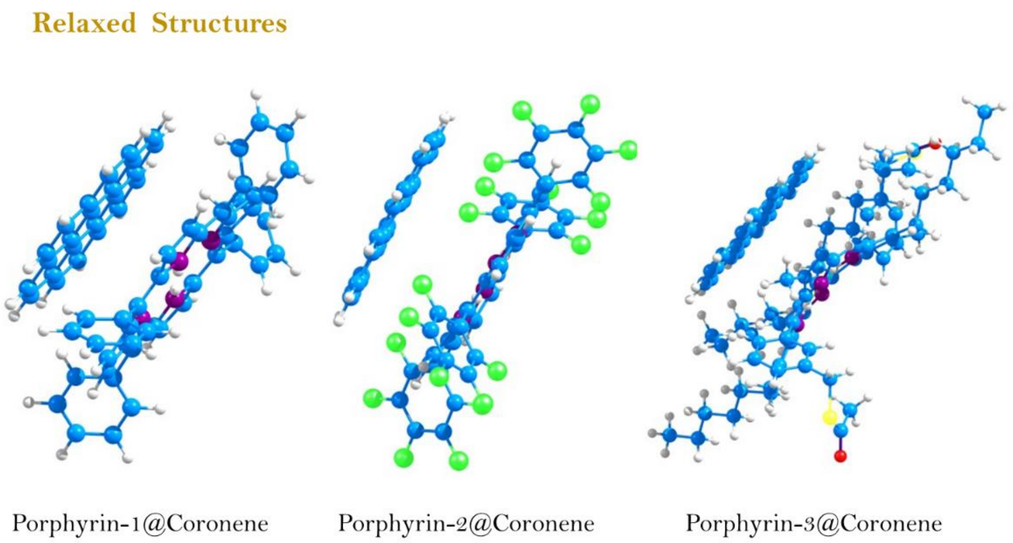

	Binding energy (eV)	
	Dispersion	No-Dispersion
Porphyrin-1@Coronene	−0.86	−0.02
Porphyrin-2@Coronene	−0.52	+0.06
Porphyrin-3@Coronene	−0.86	+0.10

Figure S64. Geometry optimisations by DFT of porphyrins-coronene hybrids.

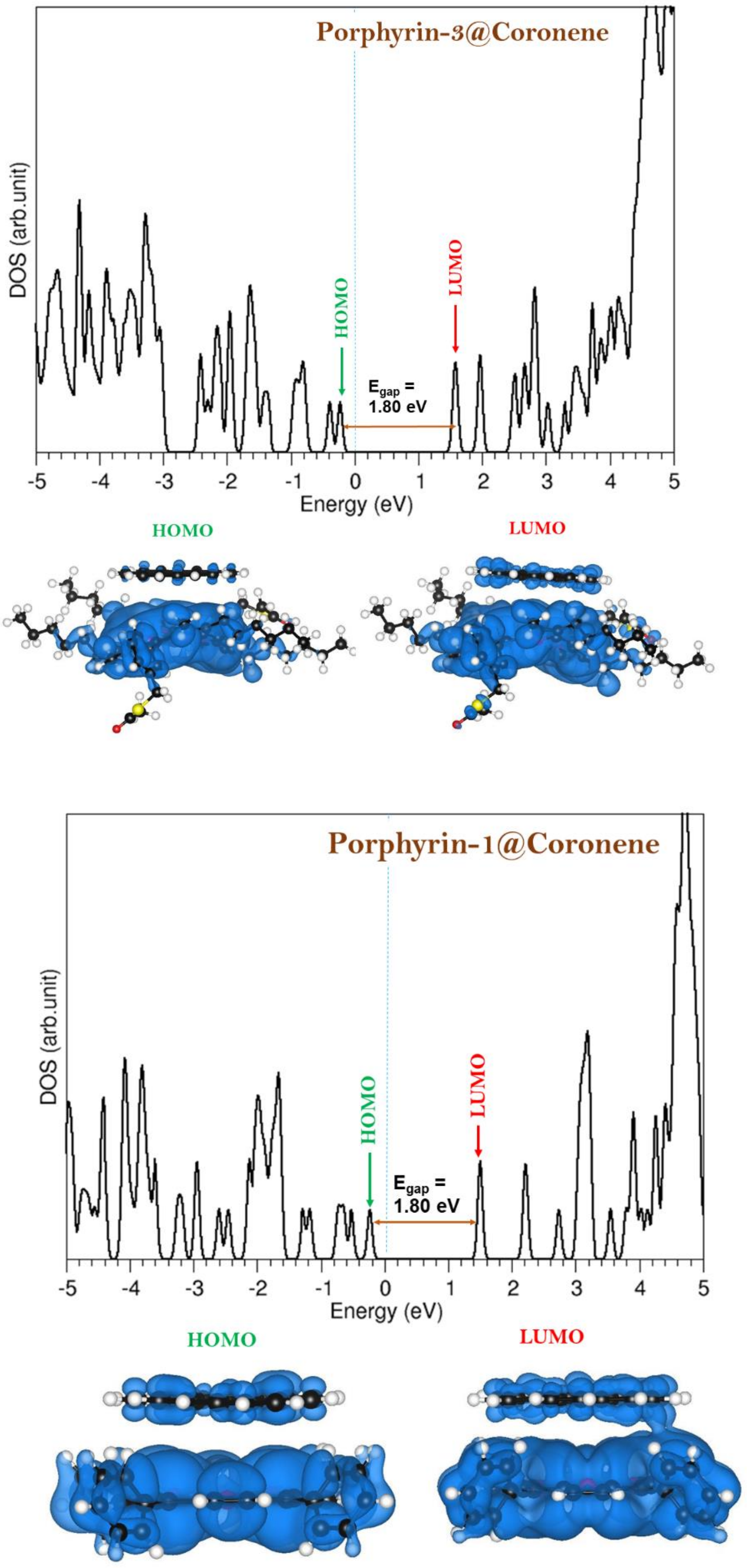

Figure S65. HOMO-LUMO Gaps and Density of States.

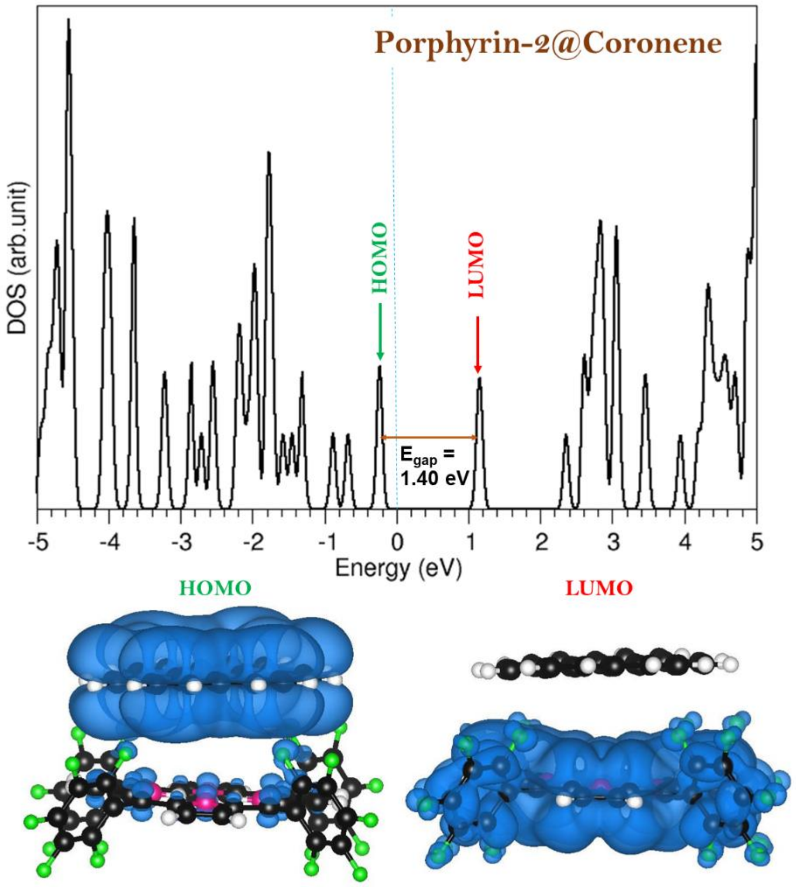

Figure S66. HOMO-LUMO Gaps and Density of States.

14. Semiempirical modelling

To describe the modes in which compounds 1-3 can interact with GO through π-π D-A interactions, the Hunter and Sander (HS) model was adopted and the calculations of electrostatic surface potentials using optimised structure of probe molecule and rGO layers were performed. The optimisation of the structures was carried out using MMFF94 Force Field in Avogadro (v 1.2.0). The electrostatic surface potentials were calculated in MarvinSpace (v 16.9.5.0)

The aromatic π-π interactions considered were the common face centred π-stacking interaction between an electron rich aromatic molecule which acts as a 'donor' and an electron deficient aromatic compound that acts as an 'acceptor'. These are a subset of aromatic interactions, which differs from electron neutral or rich aromatic stacking which is usually edge to face or in an offset conformation. In HS model, the substituents on an aromatic system either will influence the quadrupolar moment of the aromatic moiety by inducing either a partial negative or positive

charge. This difference in quadrupolar moment then determines the geometry of the interaction whether it be face-centred stacking, offset or T-shaped. The HS model successfully predicts the relative magnitude and geometry of aromatic interactions in solution.

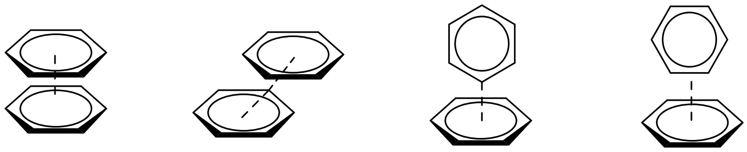

Figure S67. Schematic represenattions of π-Stacking confirmations L to R face centred, offset, T-shaped and edge to face

The interaction between the porphyrin molecules and GO were studied by electrostatic simulation. Different π-π stacking between the molecules and GO were proposed by adopting the Hunter and Sander (HS) model and carried out the calculations of electrostatic surface potentials. The strutures were optimised with MMFF94 Force Field in Avogadro software, and then the electrostatic surface potentials were modelled. By analogy with the symple systems shown in Figure S67, face-centred stacking was observed when the quadrupole moments are opposite to each other. In electron neutral aromatics such as benzene, the electron density creates a quadrupole moment where the density is concentrated above and below and plane formed by the sigma bonds. In case of electron neutral or rich aromatics the π-π interaction leads to T-Shaped or edge to face geometries as these minimize the unfavourable π-π overlap and allows interaction between the partially positive ring and an adjacent π cloud. In an electron deficient aromatic, the quadrupole moment is inverse meaning electron density is not concentrated above and below the ring. In these examples offset confirmations are observed due to allow overlap of the electron poor ring and electron rich periphery.

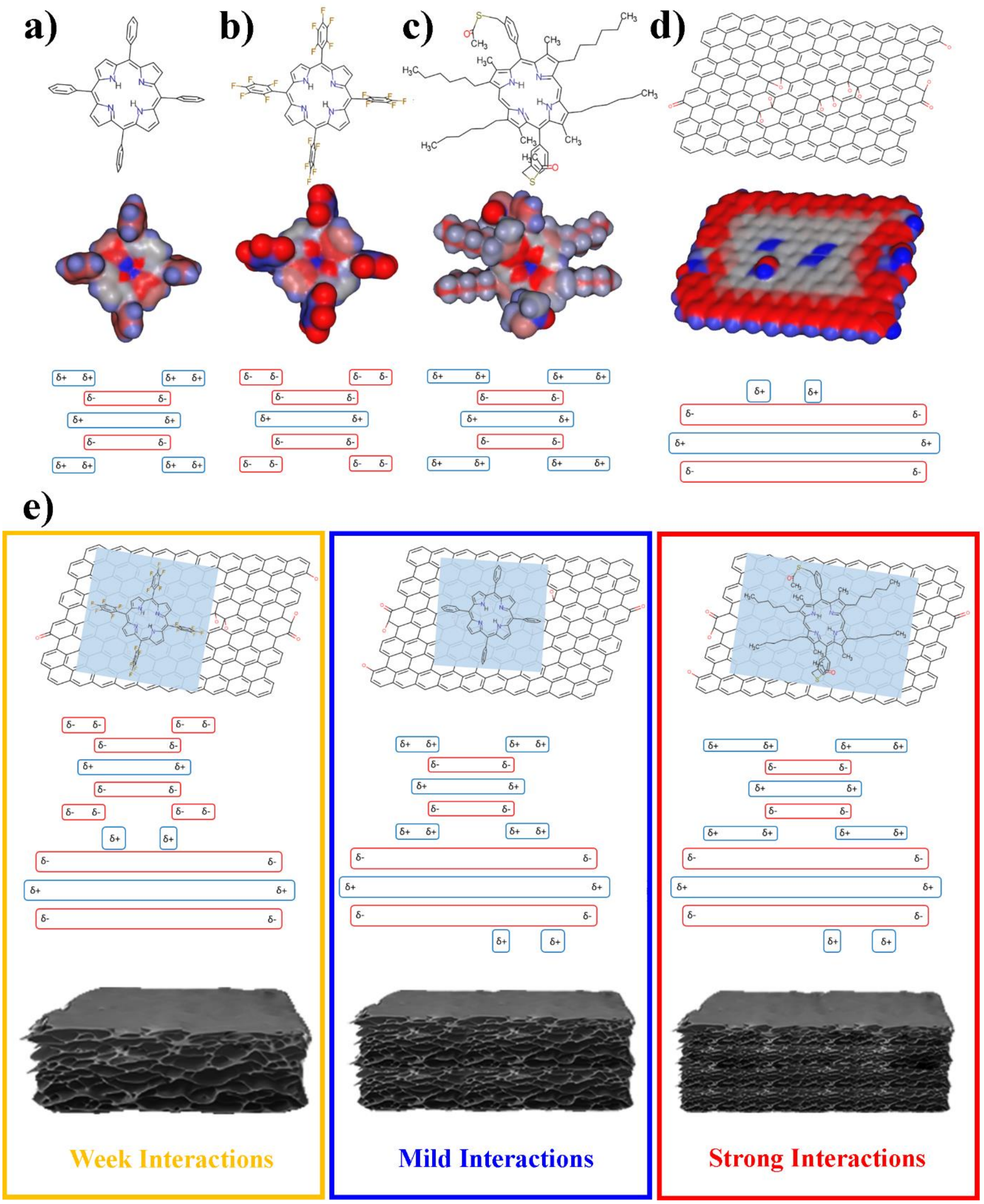

Figure S68. Semi-empirical level optimised geometries of: **(a)** compound **1**; **(b)** compound **2**; **(c)** compound **3** and **(d)** GO (red denotes areas of relatively high electron density, blue denotes electron deficient areas); **(e)** proposed stacking arrangements between compounds and GO and schemes show the formed 3D structures.

References

[1] W. S. Hummers, R. E. Offeman, *J. Am. Chem. Soc.* **1958**, 80, 1339.

[2] Y. Xu, K. Sheng, C. Li, G. Shi, *ACS nano* **2010**, 4, 4324.

[3] V. Chabot, D. Higgins, A. Yu, X. Xiao, Z. Chen, J. Zhang, *Energy Environ. Sci.* **2014**, 7, 1564.

[4] R. Gago, A. Redondo-Cubero, M. Vinnichenko, J. Lehmann, F. Munnik, F. J. Palomares, *Mater. Chem. Phys.* **2012**, 136, 729.

[5] A. Redondo-Cubero, R. Gago, F. J. Palomares, A. Mücklich, M. Vinnichenko, L. Vázquez, *Phys. Rev. B* **2012**, 86, 085436.

[6] G. Sheldrick, *Acta Crystallogr. Sect. C* **2015**, 71, 3.

[7] C. B. Hubschle, G. M. Sheldrick, B. Dittrich, *J. Appl. Crystallogr.* **2011**, 44, 1281.

[8] L. Farrugia, *J. Appl. Crystallogr.* **1997**, 30, 565.

[9] L. Farrugia, *J. Appl. Crystallogr.* **2012**, 45, 849.

[10] C. F. Macrae, P. R. Edgington, P. McCabe, E. Pidcock, G. P. Shields, R. Taylor, M. Towler, J. van de Streek, *J. Appl. Crystallogr.* **2006**, 39, 453.

[11] B. Mao, G. Calatayud David, V. Mirabello, N. Kuganathan, H. Ge, M. J. Jacobs Robert, M. Shepherd Ashley, A. Ribeiro Martins José, J. Bernardino De La Serna, J. Hodges Benjamin, W. Botchway Stanley, I. Pascu Sofia, *Chem. Eur. J.* **2017**, 23, 9772.

[12] B. Mao, G. Calatayud David, V. Mirabello, J. Hodges Benjamin, R. Martins José Alberto, W. Botchway Stanley, M. Mitchels John, I. Pascu Sofia, *Adv. Funct. Mater.* **2016**, 26, 687.

[13] H. Ge, F. Cortezon-Tamarit, H.-C. Wang, A. C. Sedgwick, R. L. Arrowsmith, V. Mirabello, S. W. Botchway, T. D. James, S. I. Pascu, *Nanoscale* **2019**, 11, 9498.

[14] P. Thordarson, *Chem. Soc. Rev.* **2011**, 40, 1305.

[15] C. Mattevi, G. Eda, S. Agnoli, S. Miller, K. A. Mkhoyan, O. Celik, D. Mastrogiovanni, G. Granozzi, E. Garfunkel, M. Chhowalla, *Adv. Funct. Mater.* **2009**, 19, 2577.

[16] B. Mao, D. G. Calatayud, V. Mirabello, N. Kuganathan, H. Ge, R. M. J. Jacobs, A. M. Shepherd, J. A. Ribeiro Martins, J. Bernardino De La Serna, B. J. Hodges, S. W. Botchway, S. I. Pascu, *Chem. Eur. J.* **2017**, 23, 9772.

[17] D. G. Calatayud, T. Jardiel, M. Peiteado, C. F. Rodríguez, M. R. Espino Estévez, J. M. Doña Rodríguez, F. J. Palomares, F. Rubio, D. Fernández-Hevia, A. C. Caballero, *J. Mat. Chem. A* **2013**, 1, 14358.

[18] M. Iwane, T. Tada, T. Osuga, T. Murase, M. Fujita, T. Nishino, M. Kiguchi, S. Fujii, *Chem. Commun.* **2018**, 54, 12443.

[19] P. Simon, Y. Gogotsi, *Acc. Chem. Res.* **2012**, 46, 1094.

[20] B. Mao, D. G. Calatayud, V. Mirabello, B. J. Hodges, J. A. R. Martins, S. W. Botchway, J. M. Mitchels, S. I. Pascu, *Adv. Funct. Mater.* **2016**, 26, 634.

[21] ChemAxon, 2016.

[22] G. Kresse, D. Joubert, *Phys. Rev. B* **1999**, 59, 1758.

[23] G. Kresse, J. Hurthmüller, *Phys. Rev. B* **1996**, 54, 11169.

[24] H. J. Monkhorst, J. D. Pack, *Phys. Rev. B* **1976**, 13, 5188.

[25] J. P. Perdew, K. Burke, M. Ernzerhof, *Phys. Rev. Lett.* **1996**, 77, 3865.

[26] S. Grimme, J. Antony, S. Ehrlich, H. Krieg, *J. Chem. Phys.* **2010**, 132, 154104.

[27] R. F. W. Bader, *Chem. Rev.* **1991**, 91, 893.
